# On the Utility of Chemical Strategies to Improve Peptide Gut Stability

**DOI:** 10.1021/acs.jmedchem.2c00094

**Published:** 2022-04-14

**Authors:** Thomas Kremsmayr, Aws Aljnabi, Juan B. Blanco-Canosa, Hue N. T. Tran, Nayara Braga Emidio, Markus Muttenthaler

**Affiliations:** † Faculty of Chemistry, Institute of Biological Chemistry, University of Vienna, Währinger Straße 38, Vienna 1090, Austria; ‡ Department of Biological Chemistry, Institute for Advanced Chemistry of Catalonia (IQAC-CSIC), Jordi Girona 18-26, Barcelona 08034, Spain; § Institute for Molecular Bioscience, The University of Queensland, St Lucia, Queensland 4072, Australia

## Abstract

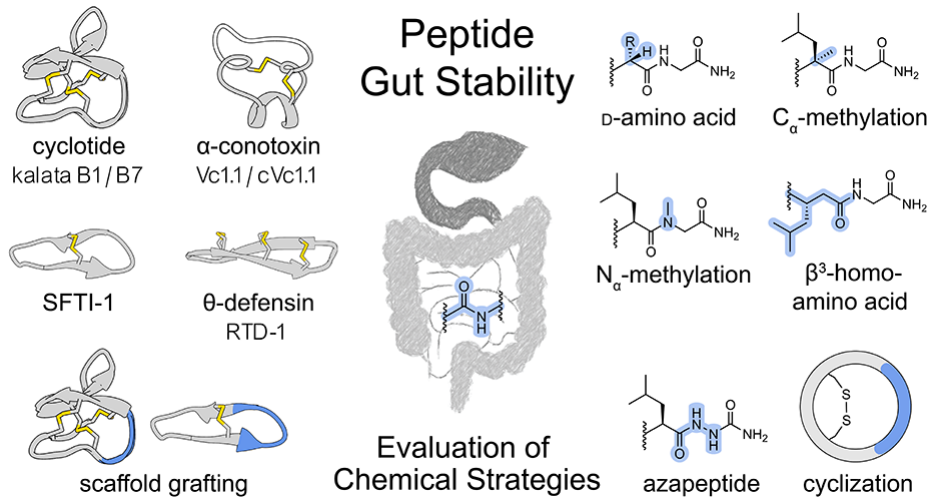

Inherent susceptibility of peptides to enzymatic degradation in the gastrointestinal tract is a key bottleneck in oral peptide drug development. Here, we present a systematic analysis of (i) the gut stability of disulfide-rich peptide scaffolds, orally administered peptide therapeutics, and well-known neuropeptides and (ii) medicinal chemistry strategies to improve peptide gut stability. Among a broad range of studied peptides, cyclotides were the only scaffold class to resist gastrointestinal degradation, even when grafted with non-native sequences. Backbone cyclization, a frequently applied strategy, failed to improve stability in intestinal fluid, but several site-specific alterations proved efficient. This work furthermore highlights the importance of standardized gut stability test conditions and suggests defined protocols to facilitate cross-study comparison. Together, our results provide a comparative overview and framework for the chemical engineering of gut-stable peptides, which should be valuable for the development of orally administered peptide therapeutics and molecular probes targeting receptors within the gastrointestinal tract.

## Introduction

Oral delivery presents one of the greatest challenges in peptide drug development.^1,2^ Although preferred by pharmaceutical manufacturers and particularly by patients, less than 10% of current peptide drugs are given orally.^3,4^ The unique physiology and physicochemical environment of the gastrointestinal tract render oral administration of peptide and protein therapeutics inherently difficult.^5^ The lining of the gut imposes three major barriers on orally ingested peptide drugs: an enzymatic barrier,^6^ a mucosal diffusion barrier,^7^ and an absorption barrier.^8^ In the stomach, parietal cells create a highly acidic environment by secreting hydrochloric acid, which sets the stage for the digestive enzyme pepsin. This endopeptidase initiates the main digestion process and preferentially cleaves peptide bonds at the site of aromatic and hydrophobic amino acids.^9^ The major digestive machinery of the gut is, however, located in the intestine, where a mixture of highly functional peptidases, lipases, and amylases (pancreatic enzymes) degrades nutrients and presents a serious stability hurdle for peptide drugs. Pancreatic peptidases that are secreted into the lumen of the intestine span a broad substrate specificity and include the endopeptidases trypsin (cleavage sites: Arg and Lys), chymotrypsin (cleavage sites: aromatic and hydrophobic residues), and elastase (cleavage sites: small hydrophobic residues) as well as the exopeptidases carboxypeptidase A (cleavage sites: aromatic, neutral, and acidic amino acids) and B (cleavage sites: Arg and Lys).^10^ In addition, a series of brush border peptidases, which are located at the surface of the intestinal epithelial lining, add up to the digestive strength of the intestine.^6^ Limited permeation through the mucus-covered gut-barrier further contributes to the recognized low oral bioavailability of peptides. The molecular size (typically >700 Da) and hydrophilic nature (H-bonding capacity) of peptides hinder the diffusion and uptake process into the bloodstream and often require the implementation of specific delivery formulations and technologies.^2,11−13^ Recent examples include the permeation enhancer-based oral formulations of the GLP-1 (glucagon-like peptide-1) receptor agonist semaglutide^14^ and an engineered insulin analogue (Novo Nordisk’s OI338 and related OI320)^15,16^ for the treatment of diabetes. Despite these advances, it remains highly challenging to develop peptide drugs with good systemic oral bioavailability (>20%).

By contrast, druggable receptors accessible within the gut lumen are increasingly recognized targets for orally administered peptide therapeutics because this strategy removes the necessity of crossing the absorption barrier.^3,17−21^ Compounds that remain peripherally restricted to the luminal side with no or negligible oral bioavailability are also often safer because of reduced risks of variable absorption and systemic side effects. Conditions that are successfully targeted *via* local luminal peptide delivery include infections, inflammatory bowel diseases (IBD, including ulcerative colitis and Crohn’s disease), celiac disease, and constipation, with about 10 compounds either on the market or in clinical development.^4,22,23^ Potential luminal accessible targets also exist for diabetes, obesity, and abdominal pain.^17,24−26^ Most successfully, orally administered peptides that activate luminal gut GC-C (guanylyl cyclase-C) receptors for the treatment of gastrointestinal disorders have emerged as a novel drug class.^18,23,27−29^ Linaclotide, a synthetic 14-mer and three disulfide bond containing GC-C agonist, is available as an oral drug in chronic idiopathic constipation (CIC) and irritable bowel syndrome with constipation (IBS-C).^30−32^ More recently, close structural analogues of the endogenous GC-C agonist uroguanylin are being considered for the treatment of the same conditions.^33^ Plecanatide, a 16-mer with two disulfide bonds, has been approved by the Food and Drug Administration (FDA) for the oral treatment of CIC and IBS-C.^34,35^ In view of the gut-specific activity of such compounds, improving peptide stability to maintain sufficient bioactivity in the hostile environment of the gut has become a central aspect in peptide drug development.

Efforts to improve the metabolic stability of peptides have been driven by considerable advances in chemical methodologies available for synthetic peptide modifications.^36−42^ To prevent proteolytic cleavage of specific amide bonds, site-directed engineering of the l-α-peptide backbone with unnatural amino acids (e.g., D-α, N_α_-alkylated, C_α_-substituted, β- and γ-amino acids)^43−47^ and amide bond mimetics (e.g., thioamides,^48^ azapeptides,^49^ 1,4-disubstituted 1,2,3-triazoles^50^) has been developed.^51^ More general strategies for molecular peptide stabilization involve polymer conjugation^52,53^ and the use of cyclization to engineer rigid structures,^42,54−58^ both of which can prevent hydrolysis by hindering protease access to cleavable bonds. N-to-C-terminal backbone cyclization and side-chain stapling *via* disulfide bonds are also key structural motifs in several natural peptide scaffolds that are proposed as stable templates to engineer drug leads *via* grafting small epitopes into their framework.^59−63^ High thermal, enzymatic, and/or serum stability has been indicated for natural and engineered versions of cyclotides,^64,65^ θ-defensins,^66,67^ sunflower trypsin inhibitor (SFTI-1),^68,69^ conotoxins,^70,71^ and chlorotoxin.^72^ However, little is known about the utility of these scaffolds and modification strategies to specifically improve gut stability.

Considering the wide implications that gut-stable oral peptide therapeutics would have on peptide drug development and patients with gastrointestinal disorders, we investigated commonly used approaches to study and enhance peptide gut stability. In particular, we (i) evaluated the gut stability of well-known disulfide-rich peptide scaffolds, approved orally administered peptide drugs, and neuropeptides and (ii) assessed several medicinal chemistry strategies to improve gut stability (independent of impact on bioactivity). We also highlighted the importance of using standardized stability test conditions to ensure reliable consistency and comparability across studies.

## Results

### Simulated Gastric Fluid (SGF) and Simulated Intestinal Fluid (SIF) Peptide Stability Assays

We used simulated gastrointestinal fluids to assess peptide stabilities under gastric and intestinal conditions. The U.S. Pharmacopeia (USP; published as U.S. Pharmacopeia-National Formulary, USP-NF, by the U.S. Pharmacopeial Convention), a major standard reference compendium in the drug development field, provides simple guidelines for SGF and SIF.^73^ In accordance with these test solution recommendations, USP-SGF and SIF are prepared as salt-containing aqueous enzyme solutions at a defined pH. The gastric environment in USP-SGF is reflected by acid-activated porcine pepsin (pH ∼1.2). USP-SIF contains pancreatin, a crude porcine mixture of peptidases, amylases, and lipases to mimic intestinal digestion at neutral pH (∼6.8). These test media are widely accepted in the field to probe stabilities of drug candidates on a preclinical R&D level, mostly because of fast turn-around data, low assay variability, easy access, absence of ethical restrictions, and solid estimation of potential metabolic cleavage sites and stabilities.^21,74,75^ However, despite the given guidelines and wide usage of USP-SGF and SIF, exact protocols and conditions are often poorly defined and vary considerably between studies in the field. As a consequence, it is difficult to compare stabilities across studies and to assess the utility of medicinal chemistry strategies to improve the gut stability of probes and therapeutic leads.^76^ One key assay variability is caused by the fluid enzyme content and activity: while the suggested (weight) content of digestive enzymes in SGF and SIF is clear according to USP guidelines, little attention is often given to the activity profiles of the employed commercial enzyme preparations (pepsin in SGF and pancreatin in SIF). We used two test peptides (somatostatin for SGF due to its known instability to pepsin^74^ and oxytocin (OT) for SIF due to its instability to pancreatic chymotrypsin^74,77,78^) to establish the impact of various commercial enzyme preparations with different activity profiles (including previously used ones in the literature) on SGF and SIF stability results (pepsin in SGF: 400 up to ≥3200 U/mg; pancreatin in SIF: 1× up to 8× USP and varying weight contents). Obtained data indicated substantial variances in peptide half-lives for different enzyme preparations in both test systems, ranging from stable within the observed time period (24 h) to almost instant degradation (Figures S1 and S2). We then defined our assay conditions in close accordance with USP guidelines and obtained highly reproducible half-lives for the test compounds in both fluids: *t*_1/2_^SGF^ [somatostatin] = 13 ± 2 min (Figure S1) and *t*_1/2_^SIF^ [OT] = 8 ± 1 min (Figure S2), which we used for further stability assessments in this study. Extensive data on the impact of different enzyme preparations on stability results and the importance of choosing the enzyme content based on activity rather than weight to ensure consistent, reproducible, and comparable SGF and SIF stability results are outlined in the Supporting Information (SI).

### Gut Stability of Disulfide-Rich Peptide Scaffolds, Oral Peptide Drugs, and Neuropeptides

**Figure 1:**
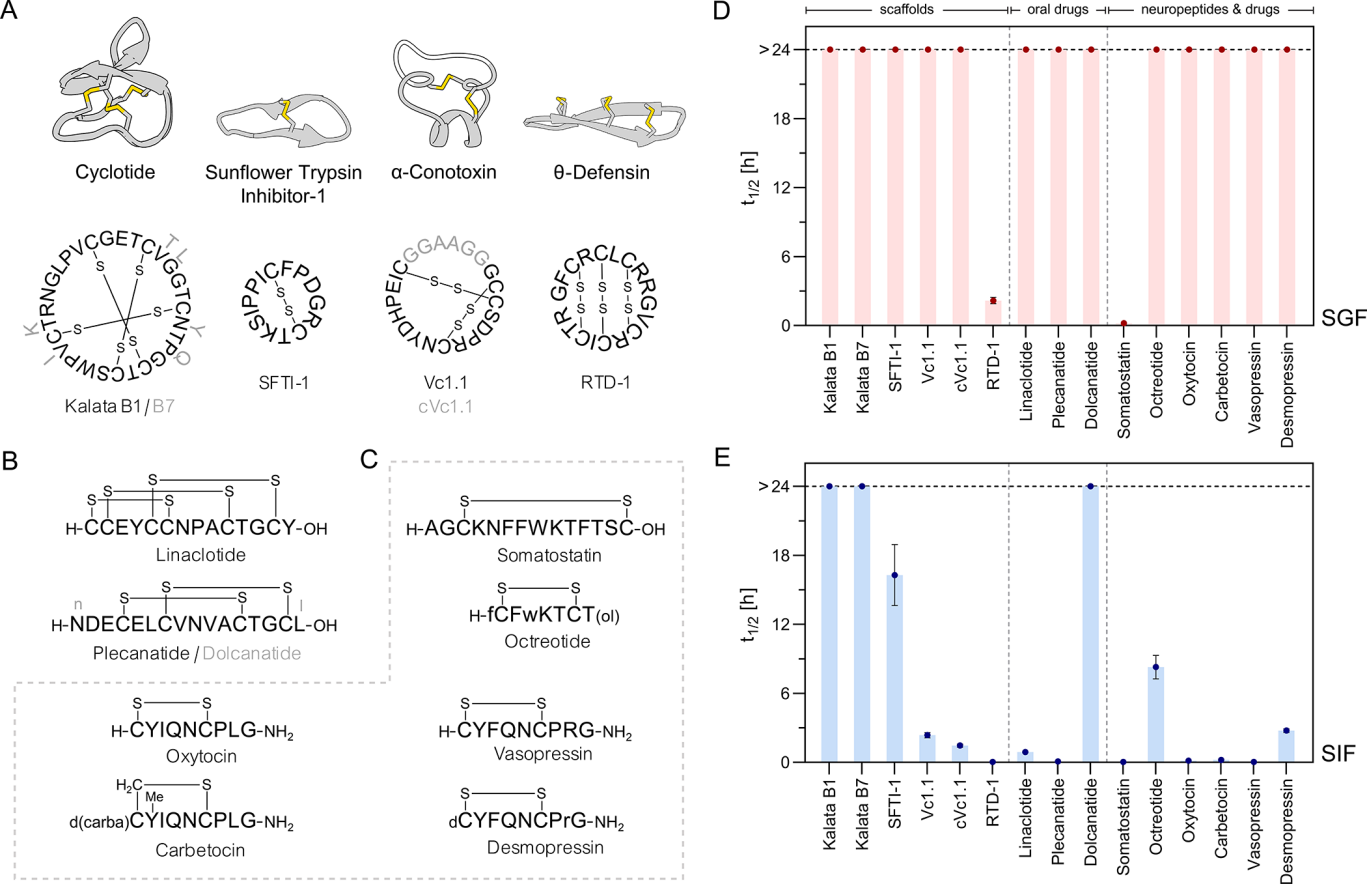
Stability of disulfide-rich peptide scaffolds, oral peptide drugs, and neuropeptides in SGF and SIF. (A) Structures and sequences of cyclic disulfide-rich peptide scaffolds that were characterized for SGF and SIF stability. Structures were accessed from RCSB PDB and visualized *via* UCSF Chimera (version 1.15):^84^ kalata B1 (1NB1), SFTI-1 (1JBL), cVc1.1(4TTL), and RTD-1 (2LYF). Cysteine connectivities (disulfide bonds) are explicitly highlighted. (B) Structures and sequences of stability-probed disulfide-rich orally administered peptide drugs. (C) Structures and sequences of neuropeptides and their chemically engineered drug analogues that were characterized for SGF and SIF stability. dC: desamino cysteine; d(carba)C: butyric acid forming a thioether with Cys; (ol): C-terminal alcohol (threonol; (2*R*,3*R*)-2-aminobutane-1,3-diol); Y(Me): *O*-methyl-tyrosine; small letters indicate d-amino acids. (D) SGF stabilities. SGF was prepared in accordance with USP recommendations using pepsin with 1200–2400 U/mg activity. (E) SIF stabilities. SIF was prepared in accordance with USP recommendations using pancreatin with 1× USP activity. Half-lives (*t*_1/2_) were calculated from one-phase exponential decay functions which were fitted to individual digestion time points (up to 10) in GraphPad Prism (mean ± standard error of mean (SEM), *n* ≥ 3). The horizontal black-dashed line indicates the latest sampling time at 24 h (stable compounds: *t*_1/2_ > 24 h). Note that some error bars are smaller than the symbols and no error could be calculated for compounds with *t*_1/2_ > 24 h. Detailed *t*_1/2_ values are presented in Table 1; for full degradation curves of all compounds, please refer to the Supporting Information.

We systematically assessed the stability of cyclic disulfide-rich peptide scaffolds from different structural classes, orally administered peptide drugs, and short cyclic neuropeptides along with their chemically engineered drug analogues in SGF and SIF (Figure 1, Table 1). We used reversed phase high-performance liquid chromatography with ultraviolet detection (RP-HPLC-UV) analysis to extract stability profiles and RP-HPLC-mass spectrometry (RP-HPLC-MS) analysis to gain insights into cleavage sites and metabolites. Samples were drawn until no intact compound was detectable or longest up to 24 h. Time points beyond 24 h (e.g., 48 h) provided inconsistent data, most likely because of enzyme inactivation and self-digestion, and were therefore not considered (data not shown). To calculate peptide half-lives (*t*_1/2_), peak areas (214 nm) at selected time points were normalized to *t*_0_ (100%) and fitted to a one-phase exponential decay function (GraphPad Prism, see the Supporting Information for complete degradation curves).

**Table 1:**
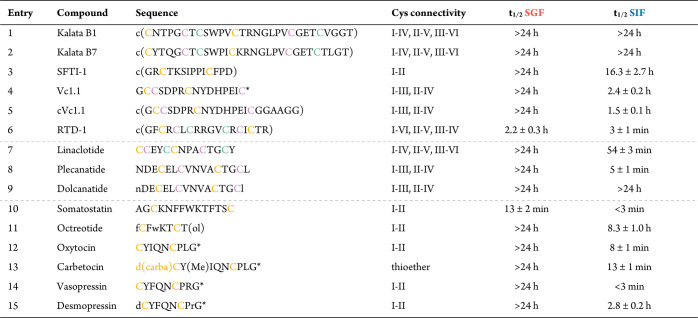
Stability Half-Lives (*t*_1/2_) of Disulfide-Rich Peptide Scaffolds, Oral Drugs, and Neuropeptides in SGF and SIF^a^

a c(), N-to-C-terminal backbone cyclic; *C-terminal amide; (ol), C-terminal alcohol (threonol; (2*R*,3*R*)-2-aminobutane-1,3-diol); d(carba)C, butyric acid forming a thioether with Cys; dC, desamino cysteine; Y(Me), *O*-methyl-tyrosine; small letters indicate d-amino acids; cysteine connectivities are color coded. Half-lives (*t*_1/2_) were calculated from one-phase exponential decay curves of *n* ≥ 3 independent experiments and presented as mean ± SEM.

Cyclotides are plant-derived N-to-C-terminal backbone cyclic peptides featuring a characteristic and evolutionary well-conserved inhibitory cystine-knot (ICK) motif (Figure 1A).^79,80^ The prototypic cyclotide kalata B1^81,82^ was stable under both SGF and SIF conditions (kalata B1: *t*_1/2_^SGF^ and *t*_1/2_^SIF^ > 24 h). The superior stability of this cyclic ICK framework was confirmed with closely related kalata B7 (*t*_1/2_^SGF^ and *t*_1/2_^SIF^ > 24 h).^83^

SFTI-1 is a natural trypsin inhibitor produced in sunflower seeds.^85,86^ This small and simple scaffold consists of a substrate-binding trypsin inhibitory loop and a cyclization loop in an overall backbone cyclic arrangement with a single disulfide bond (Figure 1A).^87^ SFTI-1 was stable in SGF with no pepsin generated metabolites detectable (*t*_1/2_^SGF^ > 24 h). Owing to its inhibitory effect on trypsin, one of the major intestinal digestive enzymes, it also had a considerable long SIF half-life of 16.3 ± 2.7 h.

The α-conotoxin Vc1.1 from the venom of *Conus victoriae* has an α-helical structure stabilized by two disulfide bonds, typical of this natural peptide class (Figure 1A, gray structure, black letters represent Vc1.1).^71,88−90^ Vc1.1 has potent analgesic properties,^25,91^ and chemical backbone cyclization of this toxin *via* a linker sequence bridging N- and C-terminus afforded compound cVc.1.1 (Figure 1A, cVc1.1 linker -GGAAGG- highlighted) with oral activity in a rat pain model (target receptor/location: GABA_B_/not fully elucidated; peripheral mechanism *via* colonic nociceptors described).^92,93^ Both peptides were resistant to pepsin degradation (Vc1.1 and cVc1.1: *t*_1/2_^SGF^ > 24 h). Interestingly, Vc1.1 displayed a slightly higher stability in SIF (*t*_1/2_^SIF^ = 2.4 ± 0.2 h) compared to the engineered backbone cyclic variant cVc1.1 (*t*_1/2_^SIF^ = 1.5 ± 0.1 h, *p* < 0.05).

θ-Defensins are cyclic peptides of mammalian origin that are produced in bone marrow and intestinal cells as part of the innate antimicrobial defense mechanism in certain species.^94,95^ Their characteristic scaffold comprises a cyclic cystine-ladder motif, consisting of two antiparallel β-strands in an N-to-C-terminal backbone cyclic arrangement with three parallel disulfide bonds (Figure 1A).^95^ We evaluated the gut stability of the prototypic rhesus θ-defensin 1 (RTD-1) scaffold from rhesus macaque.^96^ RTD-1 was not fully stable in SGF and displayed a half-life of *t*_1/2_^SGF^ = 2.2 ± 0.3 h. A large number of positively charged Arg residues in this scaffold are primary targets for the intestinal peptidase trypsin; thus, RTD-1 was even more rapidly degraded in SIF (*t*_1/2_^SIF^ = 3 ± 1 min).

We also assessed the stability of orally administered GC-C agonists (target location: gut lumen) developed for the treatment of conditions associated with chronic gastrointestinal disorders (Figure 1B). Blockbuster peptide drug linaclotide (three disulfide bonds) was stable toward pepsin degradation in SGF (*t*_1/2_^SGF^ > 24 h) and had a half-life of *t*_1/2_^SIF^ = 54 ± 3 min in SIF. In agreement with the intestinal metabolism of linaclotide, we observed an initial cleavage of C-terminal Tyr^14^ giving rise to an equally potent metabolite (desTyr^14^-linaclotide, MM-419447) which revealed high stability under SIF conditions (Figure S3).^32,75^ The recently FDA-approved GC-C agonist plecanatide (two disulfide bonds)^34,35,97,98^ also displayed high stability in SGF (*t*_1/2_^SGF^ > 24 h) but rapid degradation in SIF (*t*_1/2_^SIF^ = 5 ± 1 min). Metabolism was initiated *via* cleavage of C-terminal Leu^16^, affording an active metabolite (desLeu^16^-plecanatide, SP-338),^99^ which was quickly further degraded *via* ring opening and excision of Leu^6^ (Figure S4). The lower stability to intestinal degradation compared to linaclotide might be practically compensated by the fact that plecanatide acts (pH sensitively) in the first part of the small intestine (duodenum, pH 5–6) and is dosed higher (∼10–40-fold) than its competitor linaclotide (pH-independent activity throughout the gut).^27^ Dolcanatide is a clinical candidate for the treatment of IBD and structural analogue of plecanatide in which the terminal amino acids Asn^1^ and Leu^16^ were replaced by their D-versions to hinder exopeptidase access and improve gastrointestinal stability (Figure 1B).^97,98^ This modified variant displayed high stability in SGF (dolcanatide: *t*_1/2_^SGF^ > 24 h) and was significantly more stable under SIF conditions compared to linaclotide (dolcanatide: *t*_1/2_^SIF^ > 24 h, *p* < 0.0001), supporting this strategy.

Disulfide bond-containing neuropeptides are essential signaling molecules, and their receptors recognized drug targets under various conditions, including gastrointestinal disorders.^3,24,100−103^ We therefore probed the gastrointestinal stability of important examples along with their approved drug variants (Figure 1C). The presence of multiple sequential aromatic amino acids renders the endocrine hormone somatostatin a model substrate for pepsin activity and resulted in a short half-life of *t*_1/2_^SGF^ = 13 ± 2 min in SGF (Figures 1D and S1). We observed ring opening between Phe^7^ and Trp^8^ as the first step in somatostatin-SGF metabolism (Figure S5). Because of even swifter degradation in SIF, we were not able to determine its half-life toward intestinal peptidases (somatostatin: *t*_1/2_^SIF^ < 3 min). By contrast, octreotide, a clinically used synthetic somatostatin analogue,^104^ was fully stable in SGF (*t*_1/2_^SGF^ > 24 h). Pepsin recognition sites are here masked with d-amino acids (d-Phe^1^, d-Trp^4^), successfully hindering peptidase access. Octreotide also displayed an improved SIF stability compared to somatostatin, with *t*_1/2_^SIF^ = 8.3 ± 1.0 h (*p* < 0.01). OT and its long-acting drug analogue carbetocin were both stable in SGF (*t*_1/2_^SGF^ > 24 h). Rapid metabolism of OT in SIF proceeded *via* cleavage between Leu^8^ and Gly^9^ at the C-terminal tail (Figures S2 and S6), resulting in a short half-life of *t*_1/2_^SIF^ = 8 ± 1 min. Considering the structural identity of the C-terminal tail moiety in OT and carbetocin (Figure 1C), it was not surprising that the latter was also rapidly degraded in SIF (carbetocin: *t*_1/2_^SIF^ = 13 ± 1 min) (Figure S7). Pancreatic chymotrypsin is a key enzyme in this degradation mechanism.^77,78,105^ Vasopressin (VP), a closely related analogue to OT that only differs in two positions (Phe^3^/Ile^3^ and Arg^8^/Leu^8^), was also stable in SGF (*t*_1/2_^SGF^ > 24 h) but rapidly metabolized in SIF (*t*_1/2_^SIF^ < 3 min). The immediate degradation of VP in SIF also proceeded *via* cleavage between positions 8 and 9 (Arg^8^-Gly^9^), likely initiated by pancreatic trypsin (Figure S8).^77^ This was not the case for desmopressin, a clinically used and orally administered *V*_2_ receptor agonist (target location: kidney):^106,107^ desmopressin was stable in SGF (*t*_1/2_^SGF^ > 24 h) and the presence of d-Arg in position 8 prevented rapid metabolism in SIF (*t*_1/2_^SIF^ = 2.8 ± 0.2 h, *p* < 0.0001 compared to VP). Instead, slower metabolism occurred *via* ring opening between Tyr^2^ and Phe^3^ followed by rapid excision of Tyr^2^ (Figure S9).^78,108^

### Chemical Strategies to Improve Peptide Gut Stability Exemplified on OT

**Figure 2:**
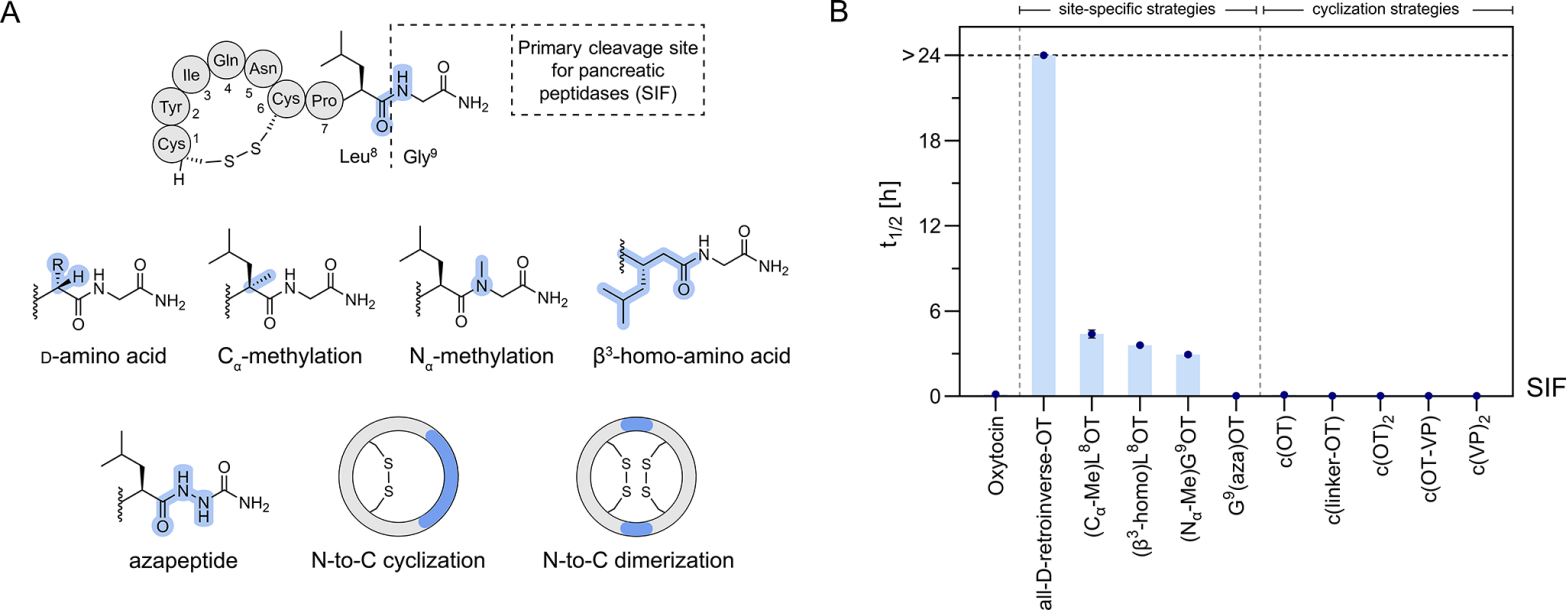
Chemical strategies to improve peptide gut stability exemplified on OT. (A) Schematic structure of OT highlighting the primary cleavage site observed in SIF (see Figure S6 for analytical details). Specific chemical modification approaches to stabilize this primary cleavage site between Leu^8^ and Gly^9^ were tested. (B) Stabilities (half-lives, *t*_1/2_) of engineered OT variants in SIF (mean ± SEM, *n* ≥ 3). The horizontal black-dashed line indicates the latest sampling time at 24 h (stable compounds: *t*_1/2_ > 24). Note that some error bars are smaller than the symbols. Detailed *t*_1/2_ values are presented in Table 2; for full degradation curves of all compounds please refer to the Supporting Information. c(), N-to-C-terminal backbone cyclic; (linker): AGAGAG; c(OT)_2_, N-to-C-terminal backbone cyclic OT dimer; c(OT-VP)_2_, N-to-C-terminal backbone cyclic OT-VP dimer; c(VP)_2_, N-to-C-terminal backbone cyclic VP dimer.

**Figure 3:**
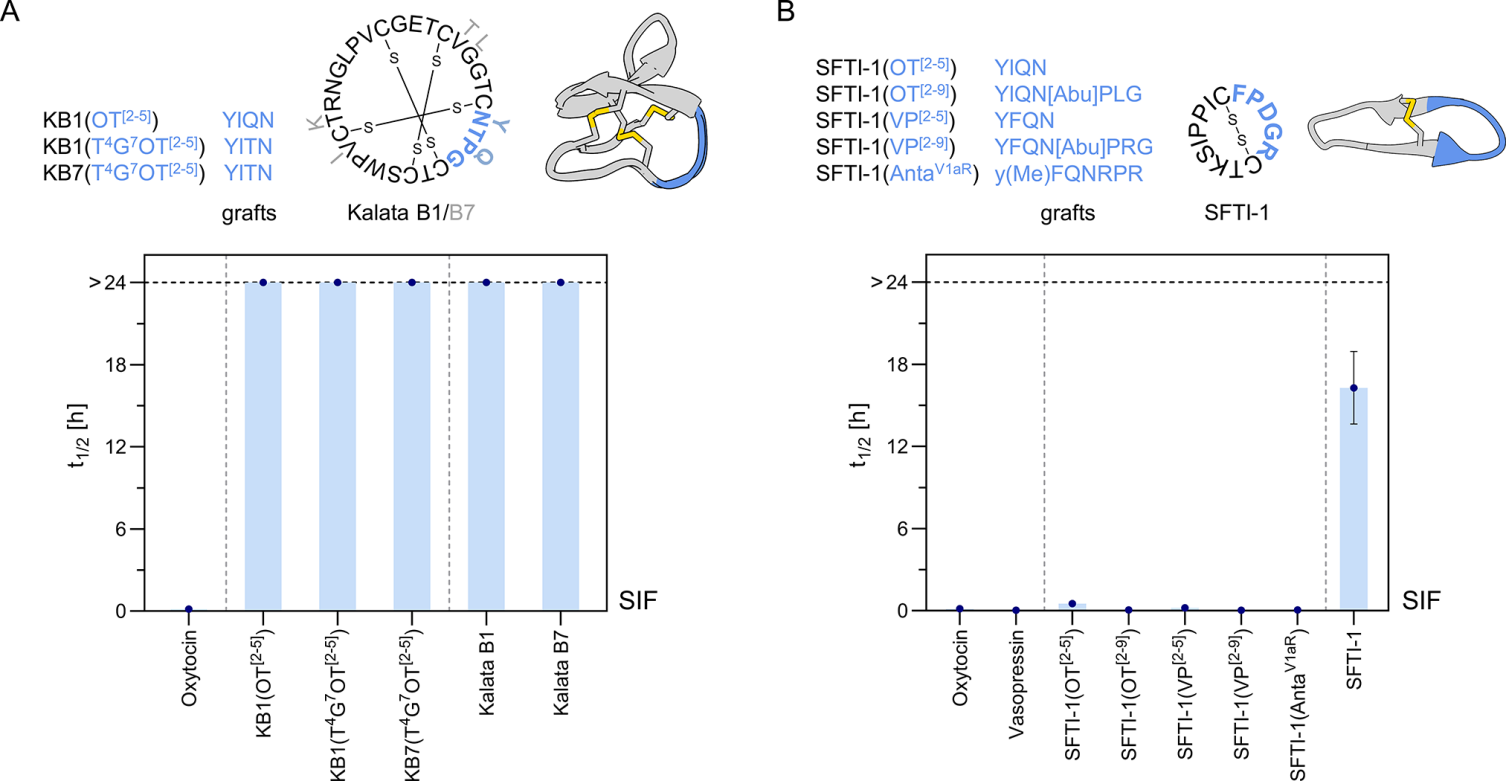
Scaffold grafting as a strategy to improve pharmacophore gut stability – exemplified with OT as a model sequence. (A) SIF stabilities of kalata B1 and B7 analogues, designed by grafting OT-like sequences into loop 3 of the scaffold. (B) SIF stabilities of SFTI-1 analogues which were designed by grafting OT/VP-like sequences into the cyclization loop, while preserving the trypsin inhibitory loop of the scaffold. Mean half-lives (*t*_1/2_) ± SEM (*n* ≥ 3) are illustrated. The horizontal black-dashed line indicates the latest sampling time at 24 h (stable compounds: *t*_1/2_ > 24). Note that some error bars are smaller than the symbols. Detailed *t*_1/2_ values are presented in Table 2; for full degradation curves of all compounds please refer to the Supporting Information. OT, oxytocin; VP, Vasopressin; Anta^V1aR^, linear VP receptor 1a antagonist sequence;^119^ Abu, aminobutyric acid, used as a Cys surrogate; superscript numbers in brackets indicate grafted residues of the OT/VP sequence: OT C^1^YIQNCPLG^9^; VP C^1^YFQNCPRG^9^.

We then systematically evaluated the utility of site-specific synthetic modifications, cyclization, and scaffold grafting strategies to improve peptide gut stability using OT as a model (Figures 2 and 3, Table 2). OT was stable in SGF but underwent rapid degradation by pancreatic peptidases in SIF (Figures 1 and S2). Based on the observed degradation path (proceeding *via* cleavage between Leu^8^ and Gly^9^) (Figure S6), we prepared a series of analogous specifically incorporating common peptide bond mimetics at the site of Leu^8^-Gly^9^ (Figure 2A). The variants were synthesized using standard Fmoc-SPPS and commercial building blocks.^109^ In the case of the aza-peptide analogue, we adopted a patent procedure.^110^ C_α_-methylation of Leu^8^ ((C_α_-Me)Leu^8^OT), the introduction of a β^3^-homo amino acid ((β^3^-homo)Leu^8^OT), and backbone N_α_-methylation ((N_α_-Me)Gly^9^OT)^111^ all prevented peptide bond cleavage in SIF and increased the half-life of OT from a few minutes to several hours ((C_α_-Me)Leu^8^OT: *t*_1/2_^SIF^ = 4.4 ± 0.3 h, (β^3^-homo)Leu^8^OT: *t*_1/2_^SIF^ = 3.6 ± 0.1 h, (N_α_-Me)Gly^9^OT: *t*_1/2_^SIF^ = 2.9 ± 0.1 h) (Figure 2B and Table 2). These results were similar to the improved metabolic stability of desmopressin in SIF (*t*_1/2_^SIF^ = 2.8 ± 0.2 h), emphasizing that these stabilization strategies were similarly effective as the use of d-amino acids and could lead to oral activity as observed with desmopressin.^106^ Masking the C-terminal peptide bond with either one of these modifications prevented peptidase access and changed the metabolic path in SIF toward the slower chymotryptic cleavage site at Tyr^2^-Ile^3^ of OT (Tyr^2^-Phe^3^ in desmopressin/VP) (Figures S9 and S10). In line with that, we used the all-d-amino acid-containing analogue, all-d-retroinverse-OT, as a stable control that as expected resisted cleavage at any targeted site and was considerably more stable in SIF (*t*_1/2_^SIF^ > 24 h). In contrast, introduction of an aza-peptide bond between Leu^8^ and Gly^9^ (G^9^(aza)OT)^112^ resulted in a variant that was metabolized even faster *via* cleavage of the C-terminal residue (*t*_1/2_^SIF^ < 3 min) (Figure S11).

**Table 2:** SIF Half-Lives (*t*_1/2_) of Chemically Engineered OT Variants in SIF^a^

entry	compound	*t*_1/2_ SIF	
1	oxytocin	8 ± 1 min	
2	all-d-retroinverse-OT	**>24 h**	****
3	(C_α_-Me)L^8^OT	4.4 ± 0.3 h	****
4	(β^3^-homo)L^8^OT	3.6 ± 0.1 h	****
5	(N_α_-Me)G^9^OT	2.9 ± 0.1 h	****
6	G^9^(aza)OT	<3 min	ns
7	c(OT)	7 ± 1 min	ns
8	c(linker-OT)	<3 min	ns
9	c(OT)_2_	<3 min	ns
10	c(OT-VP)	<3 min	ns
11	c(VP)_2_	<3 min	ns
12	KB1(OT^[2-5]^)	**>24 h**	****
13	KB1(T^4^G^7^OT^[2-5]^)	**>24 h**	****
14	KB7(T^4^G^7^OT^[2-5]^)	**>24 h**	****
15	SFTI-1(OT^[2-5]^)	31 ± 3 min	****
16	SFTI-1(OT^[2-9]^)	3 ± 1 min	ns
17	SFTI-1(VP^[2-5]^)	12 ± 1 min	ns
18	SFTI-1(VP^[2-9]^)	<3 min	ns
19	SFTI-1(Anta^V1aR^)	3 ± 1 min	ns

a Half-lives (*t*_1/2_) were calculated from one-phase exponential decay curves of *n* ≥ 3 independent experiments and presented as mean ± SEM. OT, oxytocin; VP, vasopressin; Anta^V1aR^, linear V_1a_ receptor antagonist;^119^ c(), N-to-C-terminal backbone cyclic; (linker), -AGAGAG-. Superscript numbers in brackets indicate grafted residues of the OT/VP sequence: OT C^1^YIQNCPLG^9^; VP C^1^YFQNCPRG^9^. Statistical significance was compared to OT and calculated by one-way analysis of variance (ANOVA) and multiple comparison tests (Dunnett): **** (*p* < 0.0001), ns, not significant.

We also prepared N-to-C-terminal backbone cyclic constructs of OT and VP to probe whether cyclization as a general approach is similarly effective in improving peptide gut stability as previously indicated for plasma stability.^113,114^ Fmoc-SPPS in combination with intramolecular native chemical backbone ligation was used to access these analogues.^115,116^ Two bicyclic compounds, containing one disulfide bond and an N-to-C-terminal backbone cyclization between Cys^1^-Gly^9^ (c(OT)) or *via* a linker sequence (−AGAGAG−) (c(linker-OT)), were produced and tested in SIF (*t*_1/2_^SIF^ < 3 min) (Figure 2).^113,117^ HPLC-MS analysis indicated Tyr^2^ excision from the ring structure as the starting point for digestion of these cyclic variants. In addition, tricyclic OT/VP homo- and hetero-dimers^118^ were also unstable, undergoing a cleavage at the newly introduced Gly-Cys sites (c(OT)_2_, c(OT-VP), c(VP)_2_: *t*_1/2_^SIF^ < 3 min).

Although not the primary focus of this study, we also evaluated the activity of new OT variants at the oxytocin receptor (OTR) *via* a well-established Ca^2+^-mobilization assay platform (Table S1). Most notably, C_α_-methylation of Leu^8^ did not only prevent enzymatic degradation in SIF but was also well tolerated in terms of bioactivity ((C_α_-Me)Leu^8^OT ∼ equipotent to native OT, Table S1). In addition, recent reports already indicated an ∼10-fold reduced potency of (N_α_-Me)Gly^9^OT compared to native OT,^111^ whereas all-d-retroinverse-OT was as expected inactive.^113^ In addition, N-to-C-terminal backbone cyclization and dimerization strategies applied to OT resulted in loss of activity at the OTR.^113,118^

Scaffold grafting is another chemical design approach to improve the metabolic resistance of peptide pharmacophores.^59−63^ It combines the intrinsic stability of cyclic, disulfide-rich scaffolds with the bioactivity of small epitopes to create stable and active probes. We evaluated the utility of this approach for improving the gut stability of OT-like sequences using two prototypic scaffolds: the cyclotides kalata B1/B7 (Figure 3A, Table 2) and the trypsin inhibitor SFTI-1 (Figure 3B, Table 2). We accessed all analogues employing Fmoc-SPPS and intramolecular native chemical backbone ligation followed by oxidative folding.^115,116,120−124^ Analysis in SGF and SIF indicated high stability of both natural scaffold variants (Figure 1D,E, Table 1). In the case of the cyclotide scaffold, we engineered the turn sequences of OT (OT^[2-5]^: -YIQN-) and a related variant T^4^G^7^OT (T^4^G^7^OT^[2-5]^: -YITN-)^125^ into loop 3 of kalata B1 and B7. We chose this site for grafting because loop 3 in native kalata B7 includes an OT-like type-II β-turn motif (-YTQG-), displaying weak inherent agonistic activity at the OTR.^83^ Stability analysis in SIF indicated that all the grafted variants retained the high stability of the native template scaffold (*t*_1/2_^SIF^ > 24 h), underpinning the superior and transferable stability and versatility of this ICK framework for grafting. With regard to bioactivity, however, none of the accessed OT-kalata B1/B7 grafts displayed any functional response at the OTR (Table S1), which is likely due to the larger size of the grafts not being able to sufficiently penetrate the binding pocket as described for kalata B7.^83^ In the case of SFTI-1, we designed analogues to preserve the substrate-binding trypsin inhibitory loop (-CTKSIPPIC-, black letters in Figure 3B), which is essential for the stability of the scaffold against this digestive enzyme,^68,126^ and exchanged the cyclization loop with OT- and VP-like sequences. None of the grafted SFTI-1 variants, however, retained the stability of the native template scaffold in SIF. Most of these analogues were equally or even less stable than OT/VP, suggesting strong sequence dependency on the stability of SFTI-1 grafts in SIF. Of note, OT/VP-SFTI-1 grafts also did not display any functional activity in Ca^2+^-mobilization assays (Table S1).

## Discussion

The digestive environment of the gut is a major hurdle for the development of oral peptide drugs. On the other hand, orally administered, gut-stable peptides with low oral bioavailability present a promising new drug class for gut-specific, nonsystemic interaction with luminally accessible gut receptors. Such candidates must resist an evolutionarily highly optimized machinery of endo- and exopeptidases that hydrolyze peptide bonds under strongly acidic (stomach) to neutral (intestine) pH conditions. Few studies of chemically developed or nature-derived peptides exist that fulfill these criteria, and the structural features conferring these exceptional stability properties are not well characterized. The latter aspect is also due to broadly varying stability assay types and conditions used to characterize peptide stabilities, which limits the comparability across studies.^76^ Moreover, human serum stability, the most widely used and reported stability assessment for peptides (relevant because the majority of peptide drugs are injected),^76,127^ is not a suitable measurement for the development of oral peptide drugs because it does not correlate well with gastrointestinal stability. We thus pursued a systematic and comparative gastrointestinal stability analysis of 33 peptides from different structural classes including nature-derived and chemically engineered variants (Figures 1−3; Tables 1 and 2) to advance our understanding and capabilities of developing gut-stable peptides. We also highlighted the importance of using well-defined simulated gastrointestinal fluid conditions to avoid broadly varying SGF/SIF peptide stabilities and improve interstudy comparability (Figures S1 and S2).

The main enzymatic barrier for orally ingested peptides is created by a cohort of luminally secreted pancreatic peptidases in the intestine. The stomach is typically less of a problem, because the transition time in this part of the gastrointestinal tract is short (∼3 h compared to ∼30 h of the whole gut transition time in healthy individuals),^128^ and delivery strategies such as acid-resistant capsules exist to bridge stomach transition.^11,129^ The digestive strength difference between the stomach and the intestine was also clearly observed in our stability screening, with SIF posing a greater stability hurdle than SGF (Figure 1, Table 1). All tested peptides revealed high stability in SGF, except for RTD-1 (*t*_1/2_^SGF^ = 2.2 ± 0.3 h) and somatostatin (*t*_1/2_^SGF^ = 13 ± 2 min), which is recognized by pepsin.

Our analysis revealed very different stability properties of tested natural peptide scaffolds in SIF (<3 min to >24 h, Figure 1, Table 1), even though they are all reportedly stable in human serum (*t*_1/2_^h.serum^ > 24 h).^67,92,126,130^ No obvious correlation between the type and number of cyclization motifs or number of disulfide bonds with SIF stability was observed. Cyclotides kalata B1 and B7 were the only natural scaffolds in our study that resisted degradation in SIF (Figure 1, Table 1). The remarkable stability of this ICK framework has been characterized and exploited in several proof-of-concept studies aimed at engineering more stable peptide drug leads^130−136^ and has already yielded a kalata B1 analogue that is in clinical development for oral treatment of multiple sclerosis.^137,138^ We also demonstrated high intestinal stability (*t*_1/2_^SIF^ > 24 h) of modified kalata B1/B7 analogues where OT-like sequences were grafted into loop 3 (Figure 3A), further supporting the utility of this scaffold. By contrast, incorporation of an OT/VP-like sequence into SFTI-1 resulted in a substantial drop in SIF stability compared to native SFTI-1 (Figure 3B), pointing to a strong sequence dependency of the stability of this scaffold. Surprisingly, also the well-structured θ-defensin RTD-1 (two antiparallel β-strands in a backbone cyclic cystine-ladder motif), which is expressed in the intestine of rhesus macaques,^94^ was rapidly degraded in SIF (*t*_1/2_^SIF^ < 3 min). Such a degradation might however not be a problem for physiological function, because such defense peptides are locally expressed by epithelial Paneth cells in crypts of the small intestine, right at the site of action.^94,95,139^

Our study further highlighted that commonly employed medicinal chemical strategies to improve the half-life of peptides in human serum or against single peptidases are not necessarily sufficient against the more complex and stronger intestinal digestion conditions.^140^ Cyclization, for example, was not effective in preventing intestinal degradation, with cVc1.1 not being more stable than Vc1.1 (Figure 1) and also various cyclization strategies for OT failing to improve stability (Figure 2). By contrast, incorporation of d-amino acids works efficiently as highlighted with several examples (e.g., plecanatide *vs* dolcanatide, somatostatin *vs* octreotide, VP *vs* desmopressin). The use of d-amino acids is a well-established approach in medicinal chemistry to mask specific cleavage sites^45,46^ and is often combined with other site-specific backbone modifications such as C_α_-methylation, N_α_-methylation, or β^3^-homo residues. The latter are often better tolerated than d-amino acids, particularly if the cleavage site sits within the pharmacophore where spatial orientation of the side chain often matters. Such site-specific insertion of a single carbon atom *via* either one of these strategies also efficiently improved the intestinal stability of OT against pancreatic peptidases (Figure 2, Table 2).

An important question is how these *in vitro* USP-SGF and SIF peptide gut stabilities translate to more complex *ex vivo* and *in vivo* settings and therefore could guide preclinical oral peptide drug development efforts. The native hormone somatostatin, for instance, is particularly unstable in the gastrointestinal environment, whereas its synthetic analogue octreotide was stable over several hours in SGF/SIF (Figure 1, Table 1), matching earlier reports.^74,141^ This considerable stability difference correlates well with *in vivo* data: oral administration of somatostatin has no biological effect,^142^ whereas octreotide is sufficiently stable to undergo absorption as an intact peptide and elicit oral activity.^55,143,144^ Similarly, SGF/SIF stability results are predicting the stability improvement and resulting oral activity of the drug desmopressin compared to endogenous and unstable VP (*t*_1/2_^SIF^ = 2.8 ± 0.2 h *vs* <3 min). Also for Vc1.1, our results demonstrated no substantial differences in SIF half-life between the cyclic engineered conotoxin cVc1.1 and the ‘linear’ version Vc1.1 (*t*_1/2_^SIF^ [Vc1.1] = 2.4 ± 0.2 h; *t*_1/2_^SIF^ [cVc1.1] = 1.5 ± 0.1 h), aligning well with recent *in vivo* pharmacokinetic data showing no significant difference in half-life and bioavailability upon oral administration of Vc1.1 and cVc1.1.^145^ Digestion experiments in SIF also accurately predicted the formation of the active metabolites from the oral drugs linaclotide (desTyr^14^-linaclotide, MM-419447)^32^ and plecanatide (desLeu^16^-plecanatide, SP-338),^99^ further supporting translational relevance of these results. It is however important to note that the environment and physiological conditions in the gut differ between species and individuals, depending on age, gender, health condition, and day time,^146−148^ and that such *in vitro* assays cannot fully reflect more complex, but also more variable human *ex vivo* and *in vivo* conditions.^149^ Our results, however, indicate that well-defined and reproducible USP-simulated gastrointestinal fluids provide translationally relevant and highly comparable peptide stability results that can foster preclinical drug development by identifying metabolic cleavage sites and offering valuable guidance for ligand improvements *via* medicinal chemistry strategies.^75,150^ Optimized leads can then be moved on to more complex yet elaborate and ethically restricted models, such as *ex vivo* stability assays using freshly collected human/animal gut fluids^15,16,74,78,149,151^ or *in vivo* studies in surgically ligated rat intestine or phase-I clinical trials.^32^

## Conclusions

In conclusion, this comprehensive gut stability analysis of a series of representative disulfide-rich peptide classes including natural scaffolds, therapeutic leads, neuropeptides, and approved peptide drugs provided several new insights and guidance for the development of gut-stable peptides. We demonstrated that only few native scaffolds and chemical modifications resisted degradation in the intestinal environment, including those that previously demonstrated high stability in other media (i.e., serum, single digestive enzymes). For instance, backbone cyclization, a frequently proposed medicinal chemistry approach to improve peptide stability, provided no tangible metabolic protection against intestinal degradation. By contrast site-specific peptide backbone modifications *via* C_α_- or N_α_-methylation, β^3^-homo amino acid and d-amino acids effectively prevented intestinal metabolism. Most natural cyclic and disulfide-rich scaffolds were not stable in intestinal fluid, particularly once modified with non-native sequences. The exception was the ICK class of plant-based cyclotides, likely because of their defense role in deterring animals eating their plant hosts,^152^ which retained evolutionarily optimized high gut stability even with non-native sequences incorporated. This renders cyclotides highly promising as gut-stable vectors to deliver therapeutic sequences inside the gut lumen. Taken together, our results provide a comparative framework and novel insights to support the development of gut-stable peptides, a highly important undertaking given the vast therapeutic potential of orally administered peptides for gut-specific action.

## Experimental Section

### Materials

Reagents and solvents were commercially obtained in analytical grade (or peptide synthesis grade) purity and used without further purification.

#### Peptide Synthesis

Standard 9-fluorenylmethoxycarbonyl (Fmoc) l- and d-amino acids and N-[(1H-benzotriazol-1-yl)(dimethylamino)methylene]-*N*-methylmethanaminium hexafluorophosphate N-oxide (HBTU) were purchased from Iris Biotech GmbH (Marktredwitz, Germany). 1-[Bis(dimethylamino)methylene]-1H-1,2,3-triazolo[4,5-*b*]pyridinium 3-oxide hexafluorophosphate (HATU), Fmoc-*N*-methyl-glycine (Fmoc-(N_α_-Me)-Gly-OH, CAS: 77128-70-2), and Fmoc-alpha-methyl-leucine (Fmoc-(C_α_-Me)-Leu-OH, CAS: 312624-65-0) were purchased from Fluorochem Ltd. (Derbyshire, UK). Fmoc-β^3^-homoleucine (Fmoc-β^3^-homo-Leu-OH, CAS: 193887-44-4) was purchased from Alfa Aesar, Thermo Fisher Scientific (Kandel, Germany). 2-Chlorotrityl chloride (2-CTC) resin (1.5 mmol/g, 100–200 mesh) was purchased from Chem-Impex International (Wood Dale, USA), Fmoc-Rink amide AM resin (0.74 mmol/g, 100–200 mesh) and Fmoc-D-Leu-Wang resin (0.70 mmol/g, 100–200 mesh) from Iris Biotech GmbH (Marktredwitz, Germany), Fmoc-Cys(Trt)-Wang resin (0.60 mmol/g, 100–200 mesh, Novabiochem) from Sigma-Aldrich, Merck (Darmstadt, Germany), Fmoc-Leu-Wang LL resin (0.32 mmol/g, 100–200 mesh) from Rapp Polymere (Tübingen, Germany), and H_2_N-Rink-ChemMatrix (0.47 mmol/g) from PCAS Biomatrix (Quebec, Canada). Dichloromethane (DCM), *N*,*N*-dimethylformamide (DMF), diethyl ether (Et_2_O), acetic acid, and trifluoroacetic acid (TFA) were purchased from VWR International (Darmstadt, Germany). Piperidine, N,N-diisopropylethylamine (DIPEA), triisopropylsilane (TIPS), NaNO_2_, NaH_2_PO_4_, NH_4_HCO_3_, guanidine hydrochloride (GdnHCl), sodium 2-mercaptoethanesulfonate (MesNa), 4-mercaptophenol, tris(2-carboxyethyl)phosphine hydrochloride (TCEP.HCl), l-glutathione reduced, 3,6-dioxa-1,8-octane-dithiol (DODT), 2-propanol (^i^PrOH), acetic anhydride (Ac_2_O), 1,1′-carbonyldiimidazole (CDI), iodine, ascorbic acid, and hydrazine monohydrate (N_2_H_4_ 64–65%) were purchased from Sigma-Aldrich, Merck (Darmstadt, Germany). Fmoc-hydrazine (9-Fluorenylmethyl Carbazate, CAS: 35661-51-9) was purchased from TCI Germany (Eschborn, Germany). Fmoc-MeDbz was prepared in house following published protocols.^120^

#### RP-HPLC(-MS) Analysis and Purification

Acetonitrile (ACN) and formic acid were purchased from VWR International (Darmstadt, Germany).

#### Peptides

Linaclotide (p.c. FL138962), octreotide (p.c. FO26520), carbetocin (p.c. FB19694), vasopressin (p.c. FV40959), and desmopressin (p.c. FD21366) were purchased from Carbosynth Ltd., (Compton, Berkshire, UK).

#### Gut *Stability Assays*

Pancreatin from porcine pancreas (4 × USP: p.c. P1750, 8 × USP: p.c. P7545) was purchased from Sigma-Aldrich, Merck (Darmstadt, Germany) and from VWR International (Darmstadt, Germany) (1 × USP: p.c. ICNA0210255720, brand MP Biomedicals). Pepsin from porcine gastric mucosa preparations were purchased from Sigma-Aldrich, Merck (Darmstadt, Germany): ≥400 U/mg, (p.c. P7125), 1200–2400 U/mg, (p.c. 77,151) (batch 1 activity: 2169 U/mg, batch 2 activity: 1584 U/mg), ≥3200 U/mg (p.c. P6887, batch activity 3200–4500 U/mg, average of 3850 U/mg used for calculations); USP reference standard (batch activity: 6.54 USP U/mg, p.c. 1,510,051)). KH_2_PO_4_, NaCl, NaOH pellets, and HCl (6 M) were purchased from VWR International (Darmstadt, Germany). Double-distilled Milli-Q water (ddH_2_O) was used for all buffer preparations.

#### Pharmacology

A FLIPR Calcium 4 Assay Kit was purchased from Molecular Devices (Sunnyvale, CA, USA), FuGENE HD transfection reagent was from Promega Corporation (Madison, WI, USA), and COS-1 cells were from American Type Culture Collection (ATCC, Manassas, VA, USA).

### General Peptide Synthesis and Purification

Peptides were assembled on a 0.1 mmol scale (1.0 mmol scale for cyclotides), using Fmoc-SPPS chemistry as previously described.^109^ Linear peptide synthesis was performed either manually (5 equiv excess of amino acids, 10 min HATU-mediated coupling cycles, 2 × 1 min Fmoc deprotection with 50% piperidine in DMF) or on a PTI Tribute Automatic Peptide Synthesizer (5 equiv excess of amino acids, 30 min HBTU-mediated coupling cycles, 2 × 5 min Fmoc deprotection with 20% piperidine in DMF). Unless otherwise stated, standard orthogonal protected Fmoc amino acids were used as follows: Fmoc-Arg(Pbf)-OH, Fmoc-Asn(Trt)-OH, Fmoc-Asp(OtBu)-OH, Fmoc-Cys(Trt)-OH, Fmoc-Gln(Trt)-OH, Fmoc-Glu(OtBu)-OH, Fmoc-Lys(Boc)-OH, Fmoc-Ser(tBu)-OH, Fmoc-Thr(tBu)-OH, Fmoc-Trp(Boc)-OH, and Fmoc-Tyr(tBu)-OH. Upon linear sequence assembly, dried peptide resins were treated with the standard cleavage cocktail TFA:TIPS:ddH_2_O = 90:5:5 for 90 min (unless otherwise specified) to deprotect side-chain groups and cleave the peptides from the solid support. TFA was removed under continuous nitrogen stream, and ice-cold Et_2_O was added to precipitate the peptides. Crude peptides were washed twice with fresh ice-cold Et_2_O (resuspended and centrifuged), dissolved in 1:1 ddH_2_O/ACN containing 0.1% TFA, and lyophilized. Crude linear peptides with purities >80% (as determined by analytical RP-HPLC analysis at 214 nm) were further processed without an intermediate purification step. Peptides were purified *via* RP-HPLC (Kromasil Classic C_4_ or C_18_ column, 21.2 × 250 mm, 300 Å, 10 μm; Phenomenex Jupiter Proteo C_12_ column, 21.2 × 100 mm, 90 Å, 10 μm for cyclotides) on a Waters Auto Purification HPLC-UV system using a flow rate of 20 mL/min, linear gradient elution of 5–55% solvent B in 50 min, and UV detection at 214 nm. Solvent A: 0.1% TFA in ddH_2_O, solvent B: 0.08% TFA in ACN. All synthesized final compounds were purified to >95% as determined by analytical RP-HPLC-UV and relative peak quantification at 214 nm (see the Supporting Information for analytical data and peptide quality control section for experimental details on final analysis).

### Peptide Analysis, Quality Control, and Concentration Determination *via* HPLC and High-Resolution (HR)-MS Analysis

*Routine reaction control and peptide analysis* was performed *via* RP-HPLC-UV-MS analysis on a Thermo Scientific Dionex Ultimate 3000 system equipped with a UV detector (214 and 280 nm) and a Thermo Scientific MSQ Plus electrospray ionization (ESI)-MS unit (positive ion mode). The following chromatographic parameters were used on a Waters XSelect CSH UPLC C_18_ XP column (3.0 × 75 mm, 130 Å, 2.5 μm): linear gradient elution (1–61% solvent B in 6 min) and a flow rate of 1 mL/min at 30 °C. Solvent A: 0.1% formic acid in ddH_2_O, solvent B: 0.08% formic acid in ACN. *Final analytical HPLC* chromatograms were recorded on a Thermo Scientific Vanquish Horizon UHPLC system with UV detection at 214 and 280 nm. The analysis was performed on a Kromasil Classic C_18_ column (4.6 × 150 mm, 300 Å, 5 μm) using the following chromatographic parameters: linear gradient elution (5–65% solvent B in 30 min) and a flow rate of 1 mL/min at 30 °C. Solvent A: 0.1% TFA in ddH_2_O, solvent B: 0.08% TFA in ACN. *Final HR-MS analysis* was performed on a Thermo Scientific LTQ Orbitrap Velos mass spectrometer coupled to a Thermo Scientific Vanquish Horizon UHPLC system. Samples were analyzed in LC–MS mode using an Acclaim C_18_ HPLC column (2.1 × 150 mm, 120 Å, 3 μm, Thermo Fisher Scientific) and the following chromatographic parameters: linear gradient elution (10–65% solvent B in 14 min) and a flow rate of 0.45 mL/min at 30 °C. Solvent A: 0.1% formic acid in ddH_2_O, solvent B: 0.1% formic acid in ACN. HR-ESI-MS spectra were recorded in positive ion mode in the range of *m/z* 300–2000 with an FT resolution of 60,000. The sum formulas of the detected ions were confirmed using Xcalibur 4.2.47 based on the mass accuracy (Δ*m/z* ≤ 5 ppm) and isotopic pattern.

#### Peptide Concertation Determination

Before use in any biological assay, peptides (stock solutions in ddH_2_O, 3 mg/mL) were quantified using HPLC-UV analysis at 214 nm and standards of known concentration (established *via* amino acid analysis). In brief, samples and standards were analyzed on a Kromasil Classic C_18_ column (2.1 × 100 mm, 100 Å, 5 μm) using the following chromatographic parameters: linear gradient elution of 5–65% solvent B in 10 min at 30 °C and a flow rate of 1 mL/min. Solvent A: 0.1% TFA in ddH_2_O; solvent B: 0.08% TFA in ACN. Peak areas, determined by manual peak integration (mean of three injections), were used to calculate unknown concentrations of samples based on known standards using Lambert–Beer’s law and calculated extinction coefficients derived from well-established formulas.^109,153^

### Preparation of Hydrazine-Loaded 2-Chlorotrityl Resin for the Synthesis of N-to-C-Terminal Backbone Cyclic Peptides

In a peptide synthesis vessel, 2-CTC resin (1.5 mmol/g, washed with DMF and swelled in 1:1 DMF:DCM for 30 min) was treated with 10 vol % hydrazine monohydrate in DMF for 30 min (twice). The solution was drained, and the resin was washed with DMF. A 5 vol % solution of MeOH in DMF was added for 15 min to cap unreacted functional groups, and the resin was thoroughly washed with DMF.^116^ The first Fmoc amino acid (5 equiv) was immediately coupled using HATU (5 equiv) mediated activation (15 min, twice), and the loading was determined *via* photometric Fmoc quantification.

### Determination of Resin Loading *via* Photometric Fmoc Quantification

Ten milligrams of dried amino acid loaded resin was treated with 10 mL 20% piperidine in DMF for 30 min. The solution was diluted 1:10 with 20% piperidine in DMF, and the UV absorbance was measured at 301 nm (*A*_301_) against a reagent blank (20% piperidine in DMF) to quantify the formed dibenzofulvene-piperidine adduct (ε = 7800 cm^–1^ M^–1^). The resin loading was calculated based on Lambert–Beer’s law using the simplified formula: loading (mmol/g) = *A*_301_/78 × 100.^154,155^

### Synthesis of SFTI-1 and Grafted Analogues

An intramolecular version of native chemical ligation (NCL) using peptide hydrazides as thioester precursors was employed for N-to-C-terminal peptide backbone cyclization.^115,116,156,157^ Subsequent oxidative folding occurred in one-pot in air and neutral to slightly basic pH. All linear sequences were assembled to give an N-terminal Cys and a C-terminal peptide hydrazide upon cleavage from the solid support (junction site for ligation: Cys-Arg for SFTI-1, Cys-Ile for all analogues).

#### Resin Loading

2-CTC resin was loaded with hydrazine; the first amino acid was coupled, and the resin loading was determined *via* photometric Fmoc quantification as described above (typical loading ∼0.5 mmol/g).

#### Peptide Synthesis

Linear sequences were further assembled on a PTI Tribute Automatic Peptide Synthesizer, cleaved from the solid support (TFA:ddH_2_O:TIPS:DODT = 90:5:2.5:2.5), and crude peptides were isolated using standard conditions described in the General Peptide Synthesis and Purification Section.

#### Cyclization and Consecutive One-Pot Folding

Crude peptide hydrazides (>80% purity) were dissolved in aqueous (aq.) 0.2 M NaH_2_PO_4_ containing 6 M GndHCl at pH 3 (peptide concentration: 2 mM) and cooled to −20 °C in ice/NaCl. NaNO_2_ (10 equiv, 0.5 M aq. solution) was added, and the mixture was stirred at that temperature for 15 min to convert the peptide hydrazides to the corresponding peptide azides. In a separate flask, MesNa (20 equiv) was dissolved in aq. 0.2 M NaH_2_PO_4_ containing 6 M GdnHCl at pH 7.5 (pH adjusted with aq. NaOH, 3 M), and the solution was added to the activated peptide azide to initiate thiolysis (final peptide concentration: 200 μM). The pH was carefully adjusted to 7.3–7.5 with aq. NaOH (1 M), and the reaction mixture was stirred in air (25 °C) until complete cyclization, and subsequent one-pot folding was achieved as indicated by HPLC-MS analysis (1–3 days).

#### Purification

The cyclization/folding mixtures were acidified to pH 2 by addition of aq. HCl (6 M), and the products were isolated *via* preparative RP-HPLC under standard conditions described above.

### Synthesis of Cyclotides Kalata B1/B7 and Grafted Analogues

Peptides were manually assembled on a H_2_N-Rink-ChemMatrix resin (1.0 mmol scale, 7-fold excess of amino acid, HBTU:DIPEA 7:10.5-fold activation, 30 min coupling time). The linear sequences were cyclized using an intramolecular version of NCL^157^*viaN-*acylurea (Nbz) thioester surrogates.^120,158,159^ Upon cyclization, the cyclotides were folded under oxidative conditions.^120−122^

#### Fmoc-SPPS of the Linear Sequences

Fmoc-Gly was coupled to the H_2_N-Rink-ChemMatrix resin followed by Fmoc-MeDbz.^120^ Sequences were elongated starting at Thr and the OT fragment grafted into loop 3 (Figure 3A) after splitting the resin (500 mg for each cyclotide analogue) followed by an N-terminal Cys (junction site for ligation: Cys-Thr for all cyclotides). Following on-resin N*-*acylurea formation, the assembled peptides were cleaved (TFA:TIPS:ddH_2_O:DODT = 92.5:2.5:2.5:2.5, 90 min) and then precipitated over cold Et_2_O. The peptide-containing pellets obtained after centrifugation were then dissolved in HPLC buffer (H_2_O:ACN = 1:1, 0.05% TFA) and lyophilized.

#### Cyclization

The crude linear peptides (final concentration ∼1 mM) were N-to-C-terminal cyclized under denaturing conditions (6 M GdnHCl, 0.2 M sodium phosphate, 0.1 M 4-mercaptophenol, 0.02 M TCEP.HCl, pH 7.0). Following cyclization, the crude reaction mixtures were purified *via* RP-HPLC, and the desired peptides were isolated and lyophilized.

#### Folding

The reduced cyclotides were diluted (50 μM final concentration) in NH_4_HCO_3_ (0.1 M)/^*i*^PrOH = 1:1, pH 8.4. To the resulting solution was added reduced glutathione (2 mM), and the mixture was stirred in air. Folding was monitored by HPLC-MS.^120−122^ After 20 h, the solution was acidified with aq. HCl (6 M) until pH 2–3, and ^*i*^PrOH was removed under vacuum. The folded cyclotides were isolated *via* preparative RP-HPLC using the general purification conditions described above and lyophilized.

### Synthesis of θ-Defensin RTD-1

An intramolecular version of NCL using a peptide hydrazide as the thioester precursor was employed for N-to-C-terminal peptide backbone cyclization.^115,116,157^ Subsequent folding occurred in one pot in air and neutral to slightly basic pH. The linear sequence was assembled to give an N-terminal Cys and a C-terminal peptide hydrazide upon cleavage from the solid support (junction site for ligation: Cys-Leu).

#### Resin Loading

2-CTC resin was loaded with hydrazine, the first amino acid (Fmoc-Leu) was coupled, and the resin loading was determined *via* photometric Fmoc quantification as described above (loading ∼0.5 mmol/g).

#### Peptide Synthesis

The linear sequence was further assembled on a PTI Tribute Automatic Peptide Synthesizer and cleaved from the solid support (TFA:TIPS:DODT = 90:5:5, 120 min), and the crude linear peptide was isolated and purified using standard conditions described in the General Peptide Synthesis and Purification Section.

#### Cyclization and Consecutive One-Pot Folding

The purified peptide hydrazide was dissolved in aq. 0.2 M NaH_2_PO_4_ containing 6 M GdnHCl at pH 3 (peptide concentration: 2 mM) and cooled to −20 °C in ice/NaCl. NaNO_2_ (10 equiv, 0.5 M aq. solution) was added, and the mixture was stirred at that temperature for 15 min to convert the peptide hydrazide to the corresponding peptide azide. In a separate flask, MesNa (20 equiv) was dissolved in aq. 0.2 M NaH_2_PO_4_ containing 6 M GdnHCl at pH 7.5 (pH adjusted with aq. NaOH, 3 M), and the solution was added to the activated peptide azide to initiate thiolysis (final peptide concentration: 200 μM). The pH was carefully adjusted to 7.3–7.5 with aq. NaOH (1 M), and the reaction mixture was stirred in air (25 °C) until complete cyclization, and subsequent one-pot folding was achieved as indicated by HPLC-MS analysis (1 h).

#### Purification

The cyclization/folding mixture was acidified to pH 2 by addition of aq. HCl (6 M), and RTD-1 was isolated *via* preparative RP-HPLC under standard conditions described above.

### Synthesis of Plecanatide and Dolcanatide

An orthogonal Cys(Trt)/Cys(Acm) protecting group strategy was used to access plecanatide and dolcanatide: Cys^4^(Trt)/Cys^12^(Trt) and Cys^7^(Acm)/Cys^15^(Acm). Note that the change of the folding order (i.e., using Cys^4^(Acm)/Cys^12^(Acm) and Cys^7^(Trt)/Cys^15^(Trt)) resulted in wrong topoisomers (reduced activity, data not shown) with slightly different retention times.

#### Peptide Synthesis

Linear sequences were assembled on a PTI Tribute Automatic Peptide Synthesizer and cleaved from the solid support, and the crude peptides isolated using standard conditions described under the General Peptide Synthesis and Purification Section. Preloaded Fmoc-Leu-Wang LL resin (0.32 mmol/g) was used for plecanatide and an Fmoc-d-Leu-Wang resin (0.70 mmol/g) for dolcanatide (resins were swelled in DMF for 2 h).

#### Formation of the First Disulfide Bond *via* Oxidative Folding

The crude linear peptides were dissolved in aq. 0.1 M NH_4_HCO_3_ at pH 8.2 (peptide concentration: 200 μM) and stirred in air (25 °C) until complete formation of the first disulfide bond was indicated by HPLC-MS analysis (∼24 h). The bis-Cys(Acm) containing peptides with one disulfide bond were isolated *via* preparative RP-HPLC under standard conditions described above.

#### Formation of the Second Disulfide Bond *via* Oxidative Folding

Intermediates with one disulfide bond were dissolved in 40 vol % aq. acetic acid (peptide concentration: 200 μM). Iodine (20 equiv) was dissolved in a minimum amount of MeOH and added to the peptide solution to induce simultaneous deprotection of Cys(Acm) and formation of the second disulfide bond. Upon completion (30 min, as indicated by HPLC-MS analysis), the reaction was quenched by addition of ascorbic acid (complete decolorization).

#### Purification

The oxidation solutions were diluted with ddH_2_O to 5 vol % acetic acid content and final plecanatide and dolcanatide were isolated *via* preparative RP-HPLC using the general purification conditions described above.

### Synthesis of Somatostatin

#### Peptide Synthesis

The linear sequence was manually assembled on a preloaded Fmoc-Cys(Trt)-Wang resin (0.60 mmol/g, swelled in DMF for 2 h) and cleaved from the solid support, and the crude peptide was isolated using standard conditions described above under the General Peptide Synthesis and Purification Section.

#### Oxidative Folding

The linear precursor was folded in aq. 0.1 M NH_4_HCO_3_ at pH 8.2, in air, and at 25 °C (3 days as indicated by analytical HPLC-MS analysis, peptide concentration: 200 μM).

#### Purification

Somatostatin was isolated *via* preparative RP-HPLC using the general purification conditions described above.

### Synthesis of OT, all-d-retroinverse-OT, (C_α_-Me)L^8^OT, (β^3^-homo)L^8^OT, and (N_α_-Me)G^9^OT

#### Peptide Synthesis

All OT variants were manually assembled on an Fmoc-Rink amide AM resin (0.74 mmol/g, swelled in DMF for 2 h) using commercial Fmoc-building blocks. Linear sequences were cleaved from the solid support, and crude peptides were isolated following standard procedures described under the General Peptide Synthesis and Purification Section.

#### Oxidative Folding

The linear precursors were folded in aq. 0.1 M NH_4_HCO_3_ at pH 8.2, in air, and at 25 °C (overnight, as indicated by analytical HPLC-MS analysis, peptide concentration: 200 μM).

#### Purification

Folded products were isolated *via* preparative RP-HPLC using the general purification conditions described above.

### Synthesis of G^9^(aza)OT

The procedure followed an adapted version of a patented synthetic route for the aza-peptide drug goserelin.^110^

#### Resin Loading

Fmoc-Rink amide AM resin (0.74 mmol/g) was swelled in DMF for 2 h. Upon Fmoc removal (50% piperidine, 2 × 1 min), the resin was washed with DMF and treated with a solution of CDI in DMF (5 equiv, 0.25 M, 2 × 20 min). The resin was washed with DMF, and a solution of Fmoc-hydrazine in DMF (2.5 equiv, 0.25 M) was added (2 × 30 min). Unreacted functional groups were capped *via* acetylation (6 vol % Ac_2_O and 3 vol % DIPEA in DMF, 2 × 10 min). The resin loading was determined *via* photometric Fmoc quantification as described above (loading: 0.25 mmol/g).

#### Peptide Synthesis

The remaining sequence was manually assembled and cleaved from the resin using standard conditions described under the General Peptide Synthesis and Purification Section.

#### Oxidative Folding

The linear precursor was folded in aq. 0.1 M NH_4_HCO_3_ at pH 8.2, in air, and at 25 °C (overnight, as indicated by analytical HPLC-MS analysis, peptide concentration: 200 μM).

#### Purification

The folded product was isolated *via* preparative RP-HPLC using the general purification conditions described above.

### Synthesis of N-to-C-Terminal Backbone Cyclic OT/VP Analogues

Backbone cyclic OT/VP analogues were accessed as previously described,^118^ employing an intramolecular NCL strategy with peptide hydrazides as thioester precursors and orthogonal Cys(Trt)/Cys(Acm) protection for cyclic OT/VP dimers.^115,116,157^

### USP-Simulated Gastric Fluid

The SGF composition met test solution criteria specified by the USP (USP 42 - NF 37, 2019).^73^ Preparation of 10 mL SGF, pH 1.2: NaCl (20 mg, 2 mg/mL) was dissolved in ddH_2_O, and the final pH (±0.1) was adjusted with aq. HCl (3 M) to give 10 mL solution at pH 1.2. Pepsin (32 mg, 1200–2400 U/mg) was added, and the mixture was vortexed for 1 min and sonicated for 15 min at 25 °C. The solution was centrifuged and syringe-filtered before use. See the Supporting Information for further technical details of SGF preparation and the impact of different pepsin products with various activities on peptide stability in SGF.

### USP-Simulated Intestinal Fluid

The SIF composition met test solution criteria specified by the USP (USP 42 - NF 37, 2019).^73^ Preparation of 10 mL fluid, pH 6.8: KH_2_PO_4_ (68 mg, 6.8 mg/mL) was dissolved in ddH_2_O, and the final pH (±0.1) was adjusted with aq. NaOH (3 M) to give 10 mL solution at pH 6.8. Pancreatin (100 mg, 1× USP activity) was added, and the mixture was vortexed for 1 min and sonicated for 15 min at 25 °C (the enzyme mixture does not completely dissolve). The suspension was centrifuged and syringe-filtered before use. See the Supporting Information for further technical details of SIF preparation and the impact of different pancreatin products with various activities on peptide stability in SIF.

### Stability Assay Procedure

Stock solutions (1 mM) of test peptides were prepared in ddH_2_O. SIF and SGF were prepared freshly (for each experimental replicate). At least three independent experiments (*n* ≥ 3) per compound were performed. The following sampling procedures represent a single independent experiment (*n* = 1).

#### Sampling

SGF/SIF (570 μL) was preincubated in a thermo shaker at 37 °C for 15 min. The peptide stock solution (30 μL) was added to the fluid, and the mixture was vortexed and incubated at 37 °C (600 μL total volume; 50 μM final peptide concentration). Samples (30 μL, single sample) were drawn at time points 0, 2.5, 5, 15, 30, and 60 min for all compounds and additionally at 2, 4, 6, and 24 h for compounds with *t*_1/2_ > 60 min and quenched by adding to ice-cold stop solution (30 μL, for SIF: 5 vol % aq. TFA and 5 vol % aq. TFA in 8 M GdnHCl for RTD-1; for SGF: aq. 0.4 M NaHCO_3_ and MeOH for RTD-1). To avoid inaccurate stability assessment of rapidly degrading compounds, time zero samples (*t*_0_, two samples) were additionally prepared separate from later time points.

#### Sampling Time Zero t_0_

28.5 μL SGF/SIF was added to 30 μL of ice-cold stop solution to inactivate the digestive enzymes. The mixture was vortexed, and the test peptide stock solution (1.5 μL) was added. All samples were centrifuged (5 min, 16,000 × *g*) and stored at 4 °C before analysis.

### RP-HPLC-UV(-MS) Analysis of Gut Stability Samples

Analysis was performed on a Dionex Ultimate 3000 system equipped with a UV–vis detector (214 and 280 nm). From at least three independent experiments (*n* ≥ 3), at least one experiment was analyzed on a Dionex Ultimate 3000 system equipped with a UV–vis detector (214 and 280 nm) and an additional Thermo Scientific MSQ Plus ESI-MS detector to confirm the mass of the compounds during the assay and identify metabolic cleavage sites (positive ion mode); 30 μL samples were injected on a Kromasil Classic C_18_ HPLC column (2.1 × 150 mm, 100 Å, 5 μm) equipped with a guard column (C_18_, 100 Å, 5 μm, 2.1 mm). Gradient elution (5–65% solvent B in 6 min, 10% B/min) and a flow rate of 1 mL/min at 30 °C were used. Solvent A: 0.1% TFA in ddH_2_O. Solvent B: 0.08% TFA in ACN. Formic acid was used for solvent A and B as an additive for the mass coupled system instead of TFA.

### Data Analysis of Gut Stability Samples

Data were analyzed by manual peak integration at 214 nm. Peak areas (mAU × min) at individual time points were normalized to the mean value of time point zero (average of the *t*_0_ samples; prepared and drawn samples): *y*(*t*_0_) = 100%. To calculate compound half-lives (*t*_1/2_), a one-phase exponential decay function was fitted to normalized data points *via* a nonlinear regression in GraphPad Prism (Version 9). The following constraints were applied: (i) *y*(*t*_0_) constant equal to 100, (ii) plateau constant equal to 0. The data were presented as mean ± SEM of *n* ≥ 3 independent experiments. Statistical significance of stability differences (*t*_1/2_) was calculated by a two-tailed, unpaired *t*-test. For all OT-analogues, the stability (*t*_1/2_) was compared to OT, and statistical significance calculated by one-way analysis of variance (ANOVA) and multiple comparison tests (Dunnett) in GraphPad Prism (Version 9).

### Determination of Functional Activity of OT Variants

Agonist functional activity of OT-cyclotide grafts (KB1(OT^[2-5]^), KB1(T^4^G^7^OT^[2-5]^), and KB7(T^4^G^7^OT^[2-5]^)) at hOTR was evaluated *via* Ca^2+^-mobilization assay using a fluorescent imaging plate reader (FLIPR, Molecular Devices, Sunnyvale, CA) and exact experimental protocols as previously described.^118^ In brief, COS-1 cells were cultured at 37 °C and 5% CO_2_, transiently transfected with hOTR DNA as per manufacturer’s protocol (FuGENE HD, Roche), and used for measuring Ca^2+^ responses. Two days post-transfection, seeded cells (18,000 cells/well) were loaded with calcium-4 dye, and the fluorescence intensity (excitation: 470–495 nm, emission: 515–575 nm) was measured against the baseline signal (*F*_0_) upon peptide addition at various concentrations (100 pM–10 μM). Δ*F*/*F*_0_ ratios (Δ*F*: change in fluorescence from the baseline) were calculated, and the data were analyzed as %OT in GraphPad Prism (Version 9). For the remaining compounds, agonist functional activity data were generously provided by the National Institute of Mental Health’s Psychoactive Drug Screening Program (NIMH PDSP).^160^ For experimental details (FLIPR^TETRA^ Ca^2+^-mobilization assay), please refer to the PDSP website (https://pdsp.unc.edu).

## Supporting Information Available

Additional data for SGF and SIF stability assay parameters, characterization data of observed metabolites, final analytical characterization data (HPLC traces, HR mass spectra), complete SGF and SIF degradation curves for all analyzed compounds, and pharmacological data of synthesized OT variants (PDF)
Molecular formula strings (CSV)

## Abbreviations

ACN: acetonitrile
Ac_2_O: acetic anhydride
CDI: 1,1′-carbonyldiimidazole
CIC: chronic idiopathic constipation
2-CTC: 2-chlorotrityl chloride
DIPEA: N,N-diisopropylethylamine
DODT: 3,6-Dioxa-1,8-octane-dithiol
Et_2_O: diethyl ether
FDA: Food and Drug Administration
Fmoc-SPPS: 9-fluorenylmethoxycarbonyl-solid phase peptide synthesis
GC-C: guanylate cyclase-C
GdnHCl: guanidine hydrochloride
HBTU: N-[(1H-benzotriazol-1-yl)(dimethylamino)methylene]-*N*-methylmethanaminium hexafluorophosphate N-oxide
HATU: 1-[Bis(dimethylamino)methylene]-1H-1,2,3-triazolo[4,5-*b*]pyridinium 3-oxide hexafluorophosphate
IBD: inflammatory bowel diseases
IBS: irritable bowel syndrome
ICK: inhibitory cystine-knot
NCL: native chemical ligation
OT: oxytocin
^i^PrOH: 2-propanol
RTD-1: rhesus θ-defensin 1
SFTI-1: sunflower trypsin inhibitor 1
SGF: simulated gastric fluid
SIF: simulated intestinal fluid
TCEP: tris(2-carboxyethyl)phosphine
TIPS: triisopropylsilane
USP: U.S. Pharmacopeia
VP: vasopressin
