# Supplementary material for: On the Utility of Chemical Strategies to Improve Peptide Gut Stability
Source: J Med Chem. 2022 Apr 14;65(8):6191–206. doi: 10.1021/acs.jmedchem.2c00094 (PMC9059125; doi:10.1021/acs.jmedchem.2c00094)
Supplement: Supplementary file 1 — jm2c00094_si_001.pdf [file jm2c00094_si_001.pdf]

Supporting Information

On the Utility of Chemical Strategies to Improve Peptide Gut Stability

Thomas Kremsmayr,[1] Aws Aljnabi,[1] Juan B. Blanco-Canosa,[2] Hue N. T. Tran,[3] Nayara Braga Emidio,[3] and Markus Muttenthaler[1,3]*

[1] Faculty of Chemistry, Institute of Biological Chemistry, University of Vienna, Währinger Straße 38, 1090 Vienna, Austria

[2] Department of Biological Chemistry, Institute for Advanced Chemistry of Catalonia (IQAC-CSIC), Jordi Girona 18-26, 08034 Barcelona, Spain

[3] Institute for Molecular Bioscience, The University of Queensland, St Lucia, Qld 4072, Australia.

* E-Mail: markus.muttenthaler@univie.ac.at

USP-simulated gastric fluid (SGF) and simulated intestinal fluid (SIF) to assess peptide gut stability S2

SGF/SIF key metabolites of representative compounds S7

Summary of pharmacological data: OTR activity of OT variants S17

Full gut stability curves and final analysis of tested compounds S18

References S52

USP-simulated gastric fluid (SGF) and simulated intestinal fluid (SIF) to assess peptide gut stability

Frequently observed varying SGF and SIF stability assay results are mainly a result of different fluid preparations with regards to enzyme content and activity (e.g. $t_{1/2}$ of desmopressin and linaclotide in SIF with 1×USP pancreatin activity: 2.8 ± 0.2 h and 54 ± 3 min respectively; reported $t_{1/2}$ in SIF with 4×USP pancreatin activity: 30 min for desmopressin and 16 min for linaclotide).[1] These conditions can be easily harmonized (Figure S1 and S2) to ensure comparability that would greatly benefit gut stability studies and the development of peptide scaffolds and drug candidates suitable for oral application. Our systematic analysis using well-defined gastrointestinal fluid conditions, provides such basis for consistency.

In the following, further technical details on SGF and SIF preparation and the impact of different enzyme products with various activities (pepsin in SGF, pancreatin in SIF) on peptide stability results are outlined.

USP-SGF comprises an aqueous solution of sodium chloride (NaCl) and purified porcine pepsin at pH 1.2. The enzyme content is specified as 3.2 mg/mL using pepsin with an activity range of 800-2500 U/mg of protein.[2] Activity units of commercially available pepsin mixtures span a wide range and are often not comparable nor mathematically interconvertible, depending on the procedures and substrates used for activity determination.[3, 4] The activity range specifying USP-SGF is based on an assay using hemoglobin as a protease substrate (described in the USP reagents/reagent specifications section and Food Chemicals Codex, FCC).[2] We used somatostatin as a model compound for SGF peptide digestion to establish the impact of different pepsin activity ranges on SGF stability results (Figure S1): ~400 U/mg – below USP guidelines; 6.54 USP U/mg – different unit definition; 1200-2400 U/mg (two batches of the same commercial product: batch 1 activity: 1584 U/mg, batch 2 activity: 2169 U/mg) – within the recommended USP guidelines; and 3850 U/mg (commercial product declaration ≥3200 U/mg) – above USP guidelines. Somatostatin was chosen as an SGF-test peptide since it contains multiple pepsin cleavage sites and is reportedly unstable in SGF.[5] An aqueous solution of $NaHCO_3$ (0.4 M) was used to inactivate pepsin at certain time points by raising the pH to 8 (note: higher pH should be avoided due instability of certain peptides to basic conditions; methanol can be used as an equally effective stop solution for SGF, but easily can give greater errors due to its volatility which can effect peptide quantification).[6] Pepsin from porcine gastric mucosa with 1200-2400 U/mg was closest meeting USP-SGF specifications (800-2500 U/mg) of available commercial preparations and yielded a half-life for somatostatin of 13 ± 2 min (Figure S1C). Noteworthy, two different batches of this commercial pepsin preparation, with slightly different indicated batch activities (1584 and 2169 U/mg) gave identical degradation kinetics for somatostatin. USP pepsin reference standard with an indicated activity of 6.54 USP U/mg also gave a nearly identical degradation profile ($t_{1/2}$ = 16 ± 1 min), emphasizing the lack of commercial pepsin activity unit harmonization. Somatostatin digestion was slightly faster when a pepsin preparation with a digestive strength of 3850 U/mg (commercial specification: ≥3200 U/mg, indicated batch activity: 3200-4500 U/mg, average of 3850 U/mg used for calculations) was used: $t_{1/2}$ = 6 ± 1 min. In contrast, pepsin with a lower activity range of ~400 U/mg resulted in a substantially prolonged half-life for somatostatin of > 2 h. We then adjusted the (weight) content of 3850 U/mg pepsin (1.8 mg/mL) to meet the SGF digestive strength of the 1200-2400 (2169) U/mg preparation. With this simple linear calculation, we indeed obtained a reproducible stability profile (Figure S1B). Further SGF stability assays were performed using fluid preparations with 1200-2400 U/mg pepsin (3.2 mg/mL), which is on the upper limit of USP-SGF criteria to avoid overestimation of gastric peptide stablities.[2]

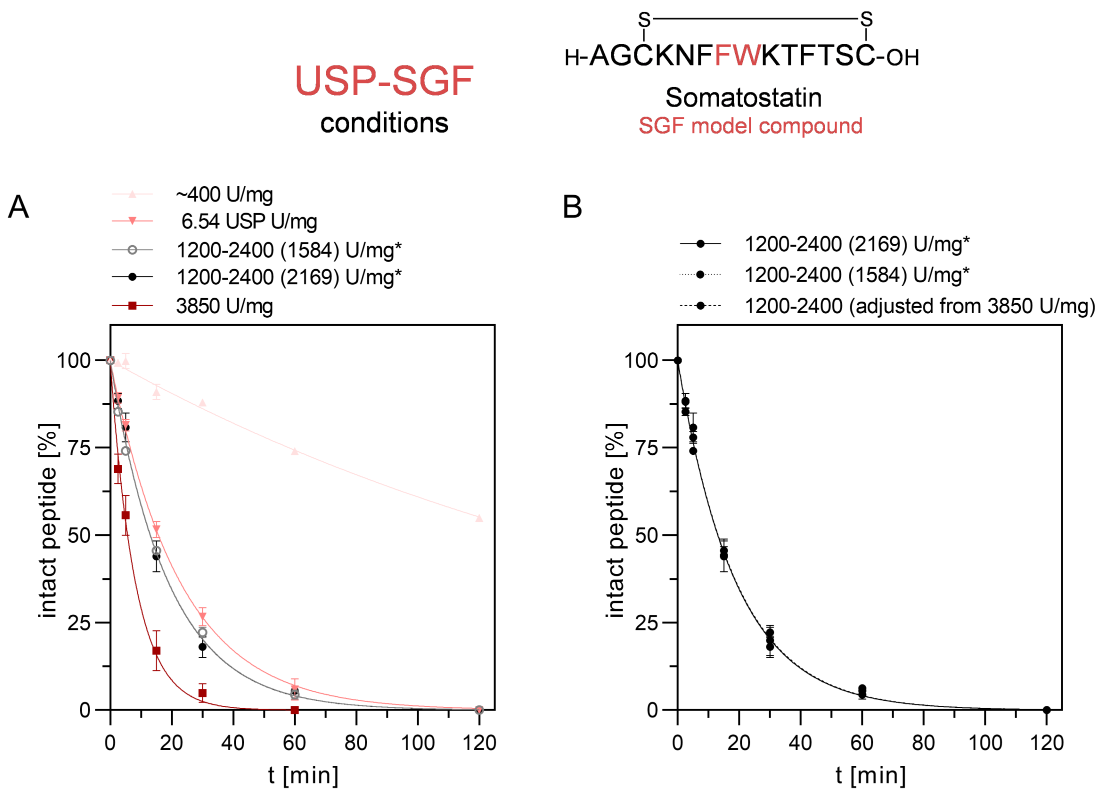

C

pepsin preparation		provider/brand	product code (p.c.)	SGF $t_{1/2}^{Somatostatin}$
~400 U/mg		Sigma-Aldrich	P7125	> 2 h
6.54 USP U/mg	USP reference standard	USP (via Sigma-Aldrich)	1510051	16 ± 1 min
1200-2400 U/mg	batch 1: 2169 U/mg<br>batch 2: 1584 U/mg	Sigma-Aldrich	77151	13 ± 2 min
≥3200 U/mg	batch: 3200-4500 (3850) U/mg	Sigma-Aldrich	P6887	6 ± 1 min

Figure S1. USP-simulated gastric fluid (SGF) peptide stability assays – revisited with somatostatin as a test peptide. A. Impact of pepsin activity on the stability of somatostatin in SGF at 37°C. SGF was prepared according to USP specifications: aq. solution of 3.2 mg/mL pepsin, 2.0 mg/mL NaCl, pH 1.2. USP-SGF pepsin activity is specified as 800-2500 U/mg. Different commercial pepsin preparations were tested to illustrate the impact of pepsin activity on SGF peptide stability. The 1200-2400 U/mg preparation met USP-SGF-criteria (upper limit) and was used for SGF peptide stability assessments in this work. **B.** Adjusting the weight content of different pepsin preparations in SGF to meet the recommended digestive strength of 1200-2400 U/mg pepsin. * two different batches of the same commercial pepsin preparation 1200-2400 U/mg, which gave the same degradation kinetics: batch 1 activity = 2160 U/mg, batch 2 activity = 1584 U/mg. Data points are presented as means ± SEM of n ≥ 3 independent experiments. Data points were fitted in GraphPad Prism via non-linear regression using a one-phase exponential decay function with a constrained Y_0 = 100 and a plateau at 0. **C.** List of commercial pepsin preparations (activity profile, provider and product code p.c.) used to prepare SGF and calculated half-lives for somatostatin in the different SGF media. The sequence of the test peptide somatostatin is illustrated with the primary cleavage site in SGF highlighted in colour.

USP-SIF constitutes an aqueous solution of monobasic potassium phosphate (KH_2PO_4) and crude porcine pancreatin at pH 6.8.[2] While the enzyme quantity in this medium is unambiguously specified as 10 mg/mL, the activity range of the pancreatic enzyme mixture used in the preparation is less clear, an issue that was often neglected in the past. According to the USP monograph for pancreatin, ≥ 25 USP Units/mg peptidase activity, ≥ 25 USP Units/mg amylase activity and ≥ 2 USP Units/mg lipase activity are required.[2] Mixtures meeting this activity profile are commonly labeled 1×USP pancreatin. Material with higher digestive strength is also commercially available and indicated accordingly as multiples of the activity ranges described for the peptidase, amylase and lipase content (e.g., 4×USP: ≥100 USP Units/mg protease activity, ≥100 USP Units/mg amylase activity and ≥8 USP Units/mg lipase activity). We used oxytocin (OT) as a model compound for SIF digestion to establish the impact of different fluid preparations with various porcine pancreatin mixtures from commercial sources on peptide stability (Figure S2). OT was considered a suitable SIF-test peptide since its C-terminal tail is a prime substrate for the pancreatic peptidase chymotrypsin (Figure S6).[7-9] An aqueous solution of TFA (5 vol%) was used to terminate the digestion process at selected time points. We initially prepared SIF using the USP specified enzyme quantity (10 mg/mL) but with pancreatin of 1×USP, 4×USP and 8×USP activity accordingly (Figure S2A). The differences in OT degradation kinetics were substantial, yielding half-lives of 8 ± 1 min (1×USP), <3 min (4×USP) and <3 min (8×USP) respectively (Figure S2C). The impact of the enzyme activity on SIF stability of OT was even more pronounced when we prepared diluted SIFs with less pancreatin content than the USP specified 10 mg/mL, as used in past studies:[10, 11] a tenth of the pancreatin quantity (0.10×USP) increased the half-life of OT to >1 h while using 0.01×USP resulted in no considerable degradation within 1 h. We then prepared SIFs by adjusting the enzyme quantities of 4×USP (2.50 mg/mL) and 8×USP (1.25 mg/mL) pancreatin to meet the proposed 1×USP activity content using the same simple linear calculations as demonstrated for pepsin in SGF. We again obtained degradation kinetics for OT consistent with 1×USP SIF activity (Figure S2B), emphasizing the importance to consider pancreatin activity over pancreatin weight content to obtain consistent digestive strengths of SIF preparations.

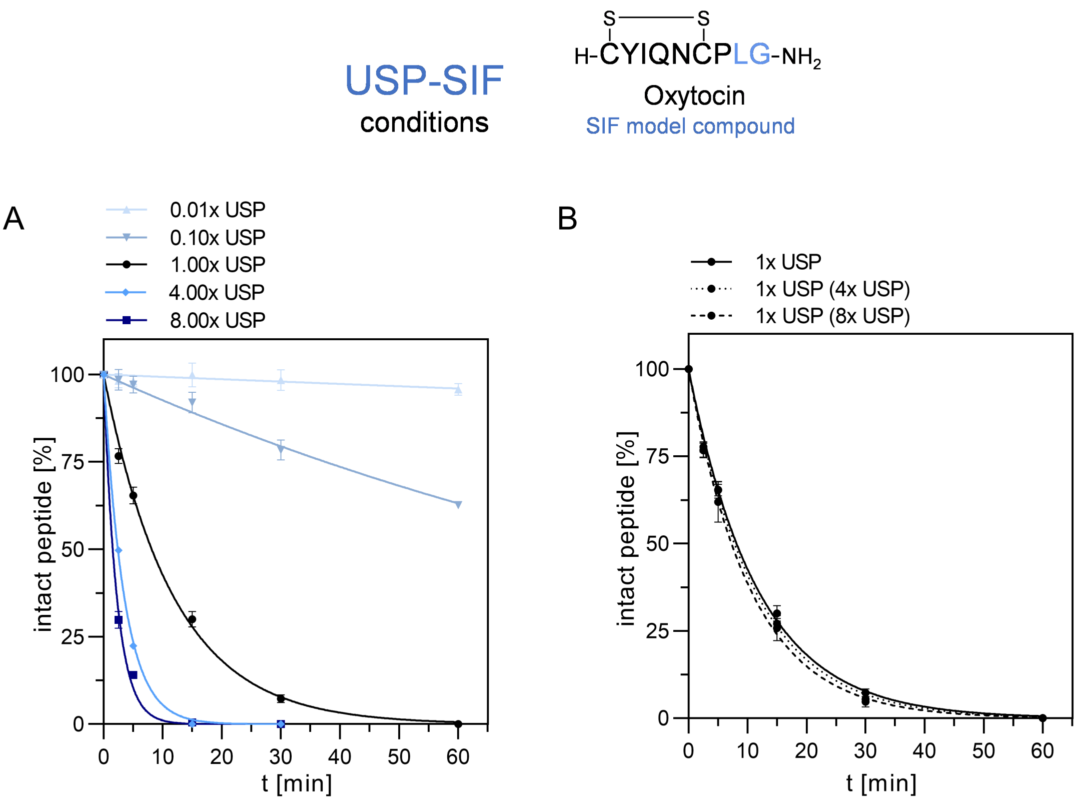

C

pancreatin preparation	provider/brand	product code (p.c.)	SIF $t_{1/2}^{Oxytocin}$
0.01x USP	not commercial, prepared by dilution		> 1 h
0.1x USP	not commercial, prepared by dilution		> 1 h
1x USP	MP Biomedicals (via VWR)	ICNA0210255720	8 ± 1 min
4x USP	Sigma-Aldrich	P1750	< 3 min
8x USP	Sigma-Aldrich	P7545	< 3 min

Figure S2. USP-simulated intestinal fluid (SIF) peptide stability assay – revisited with oxytocin (OT) as a test peptide. A. Impact of pancreatin content and activity on the stability of OT in SIF at 37°C. SIF was prepared according to USP specifications: aq. solution of 10 mg/mL pancreatin, 6.8 mg/mL KH_2PO_4, pH 6.8. According to the USP monograph for pancreatin, ≥25 USP Units/mg peptidase activity, ≥25 USP Units/mg amylase activity and ≥2 USP Units/mg lipase activity are required. Mixtures meeting this activity profile are commonly labeled 1×USP pancreatin. Different commercial pancreatin preparations were tested to establish the impact of pancreatin activity on SIF peptide stability. The 1×USP preparation was used for SIF peptide stability assessments in this work. **B.** 1×USP pancreatin content in SIF derived from various commercial pancreatin mixtures (1×USP, 4×USP, 8×USP) yielded consistent degradation kinetics for OT and is recommended to unify SIF assay protocols. Data points are presented as means ± SEM of n ≥ 3 independent experiments. Data points were fitted in GraphPad Prism via non-linear regression using a one-phase exponential decay function with a constrained Y_0 = 100 and a plateau at 0. **C.** List of commercial pancreatin preparations (activity profile, provider and product code p.c.) used to prepare SIF and calculated half-lives for OT in the different SIF media. The sequence of the test peptide OT is illustrated with the primary cleavage site in SIF highlighted in colour.

SGF/SIF key metabolites of representative compounds

Metabolism in SGF/SIF was followed by RP-HPLC-UV-ESI-MS analysis on a Thermo Scientific Dionex Ultimate 3000 system with a UV-VIS detection at 214 nm and 280 nm and coupled to a Thermo Scientific MSQ Plus ESI-MS detector (positive ion mode). Linear gradient elution (1-61% solvent B in 6 min) and a flow rate of 1 mL/min was used on a Kromasil Classic C_{18} column (4.6 × 150 mm, 300 Å, 5 µm) at 30 °C. Solvent A: 0.1% formic acid in ddH_2O, solvent B: 0.08% formic acid in ACN.

The formation of key metabolites for selected representative examples is shown *via* HPLC-UV trace at 214 nm and mass analysis of intact compounds and identified metabolites.

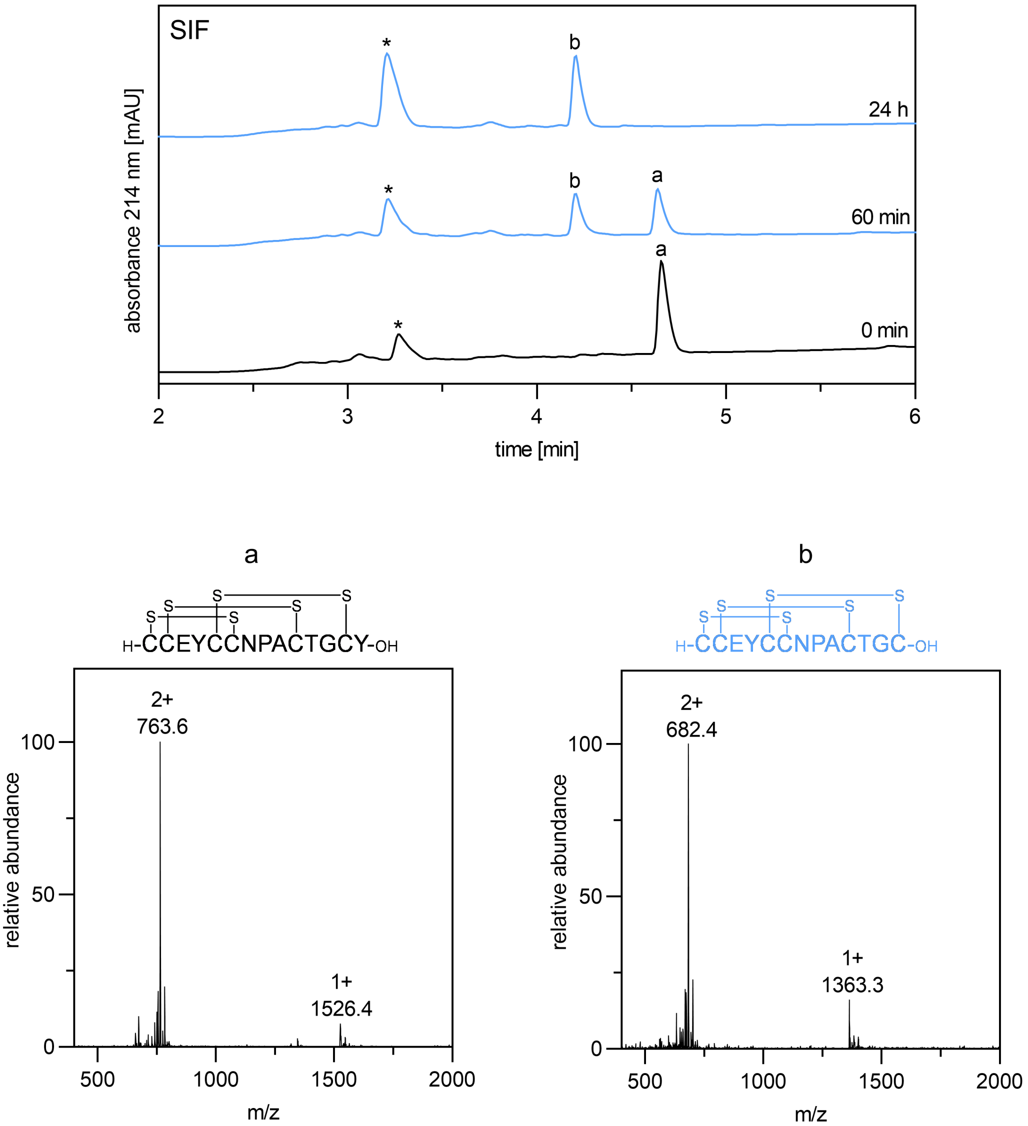

Figure S3: Primary metabolite of linaclotide in SIF. * depict SIF background peaks.

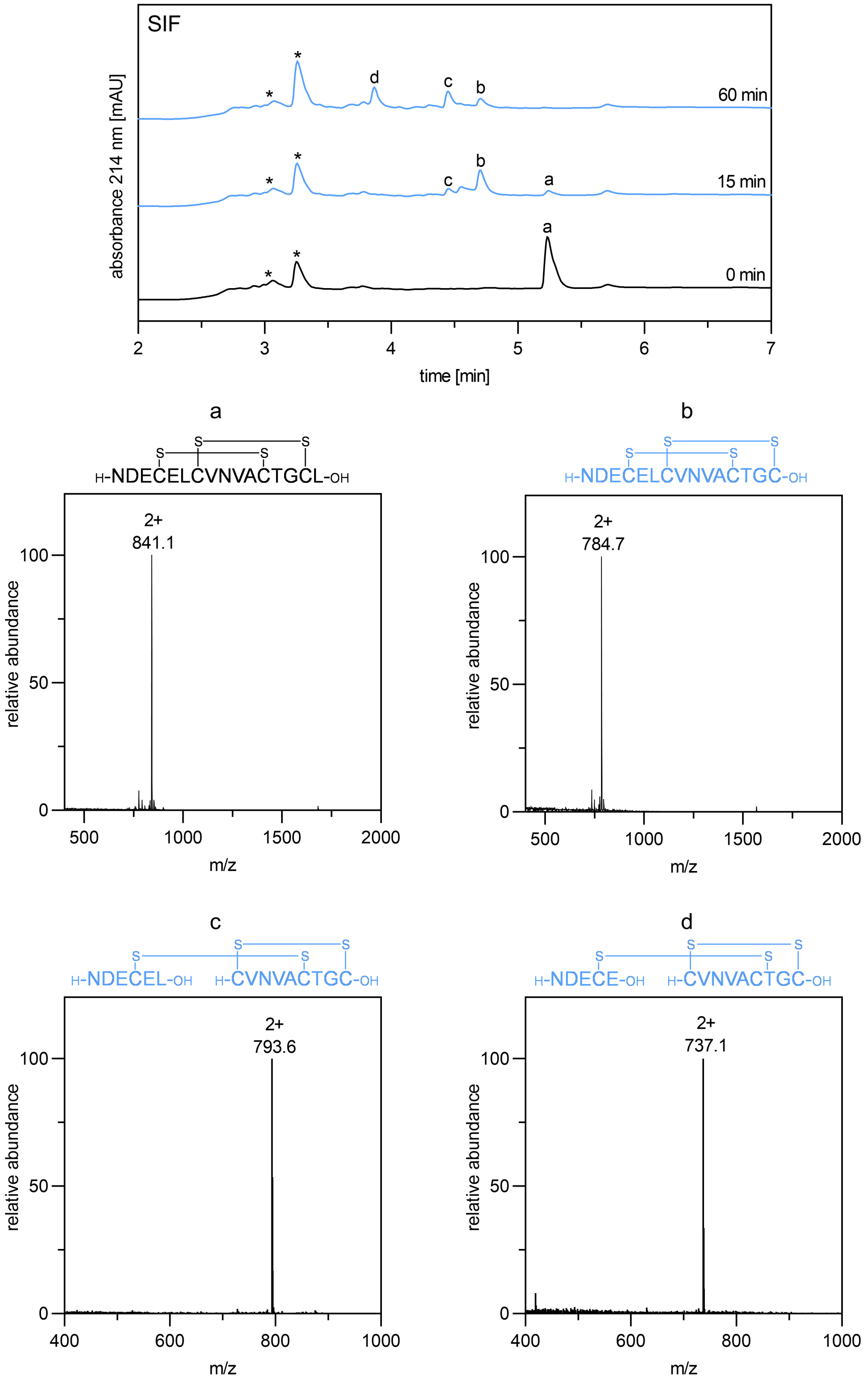

Figure S4: Primary metabolites of plecanatide in SIF. * depict SIF background peaks.

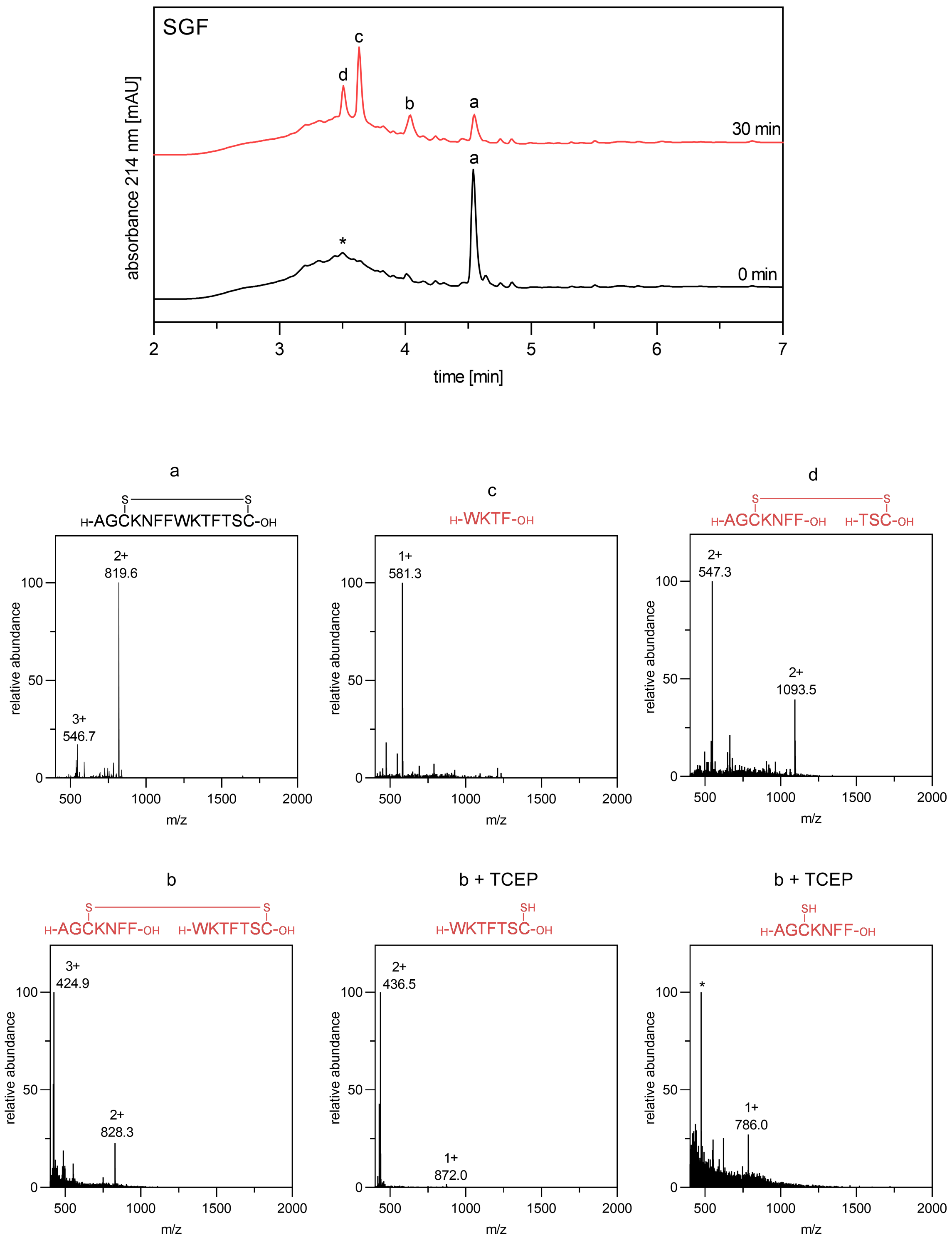

FigureS5: Primary metabolites of somatostatin in SGF. * depicts SGF background peak.

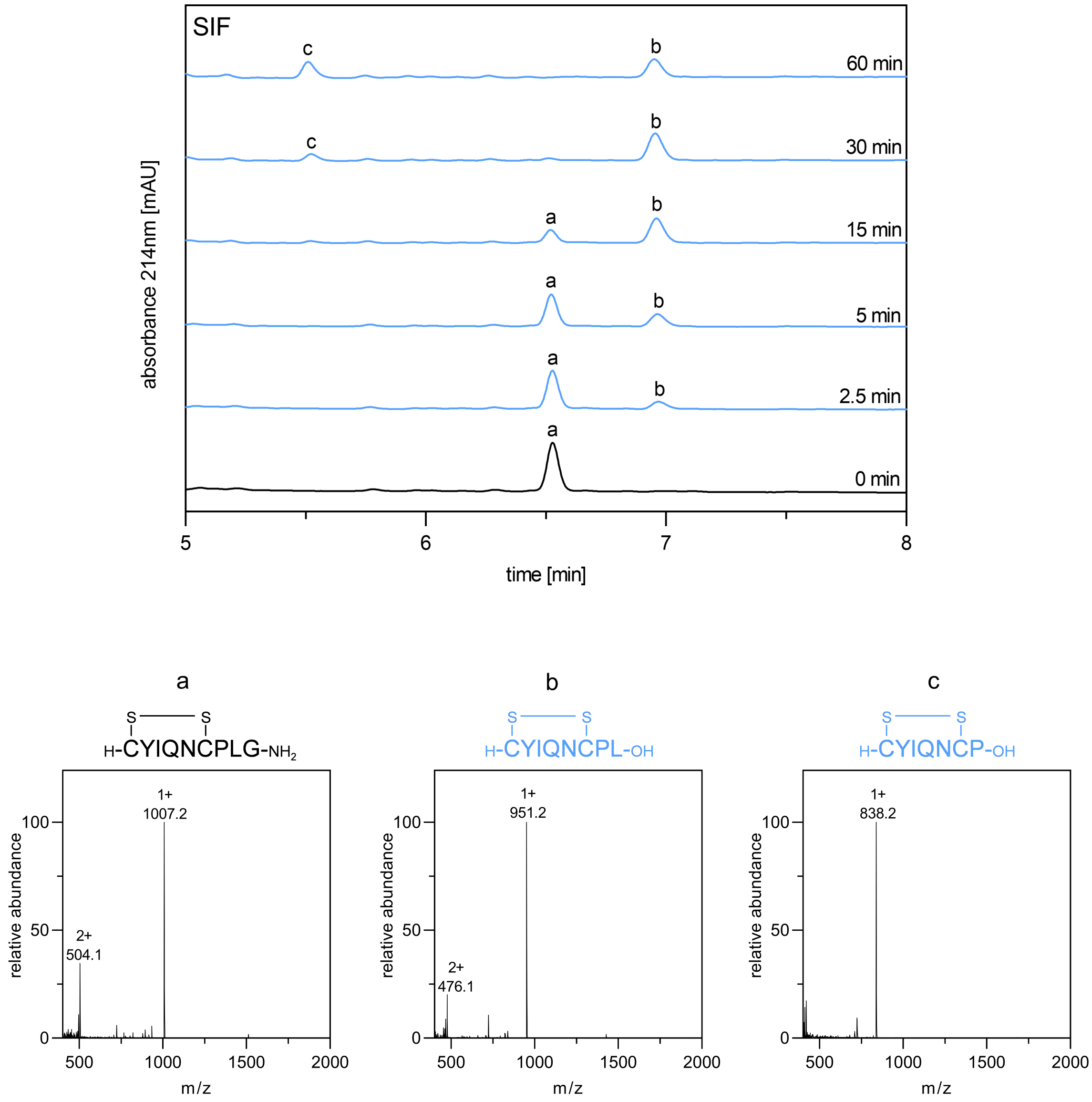

Figure S6: Metabolism of oxytocin in SIF over 60 min.

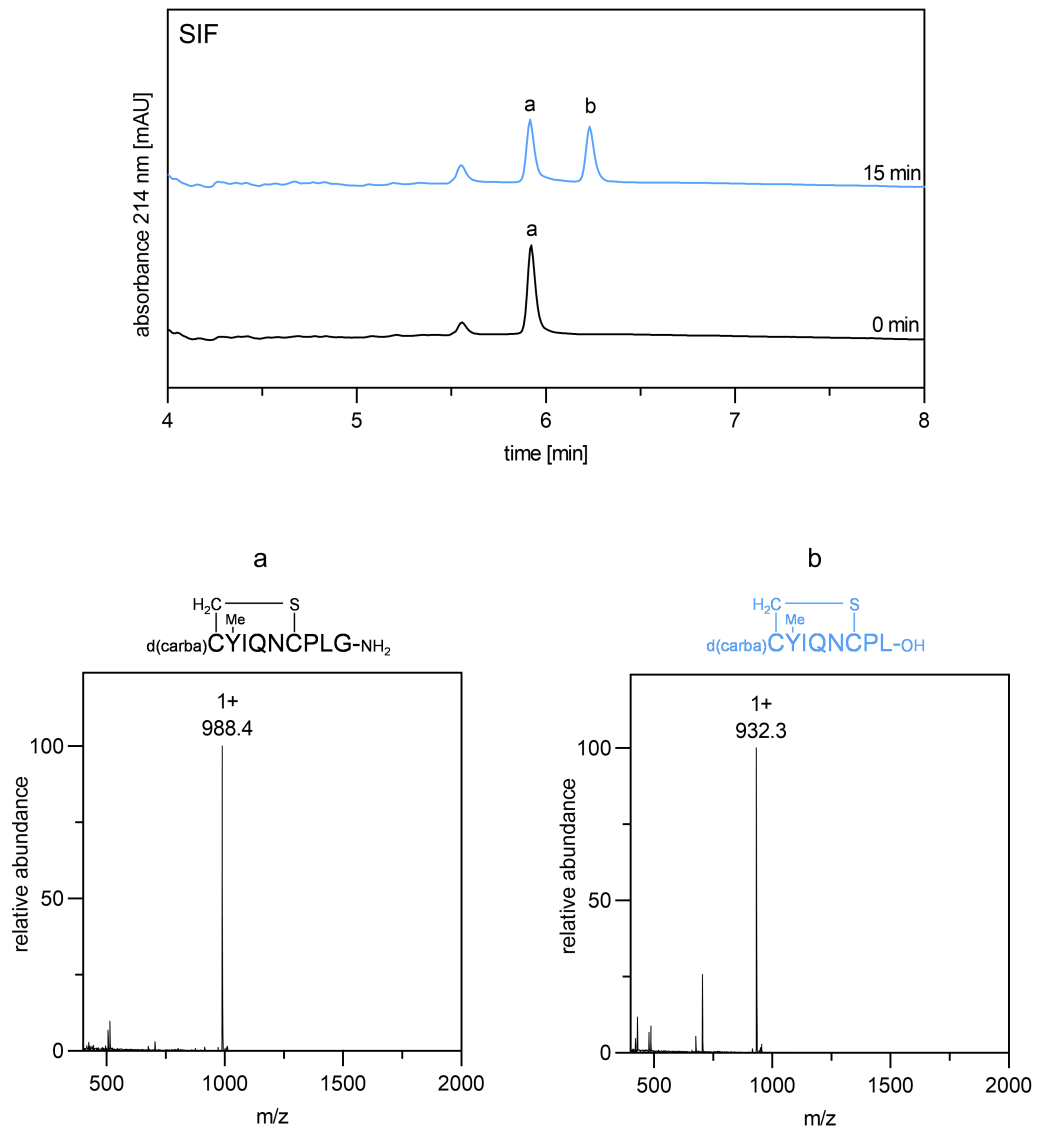

Figure S7: Primary metabolite of carbetocin in SIF.

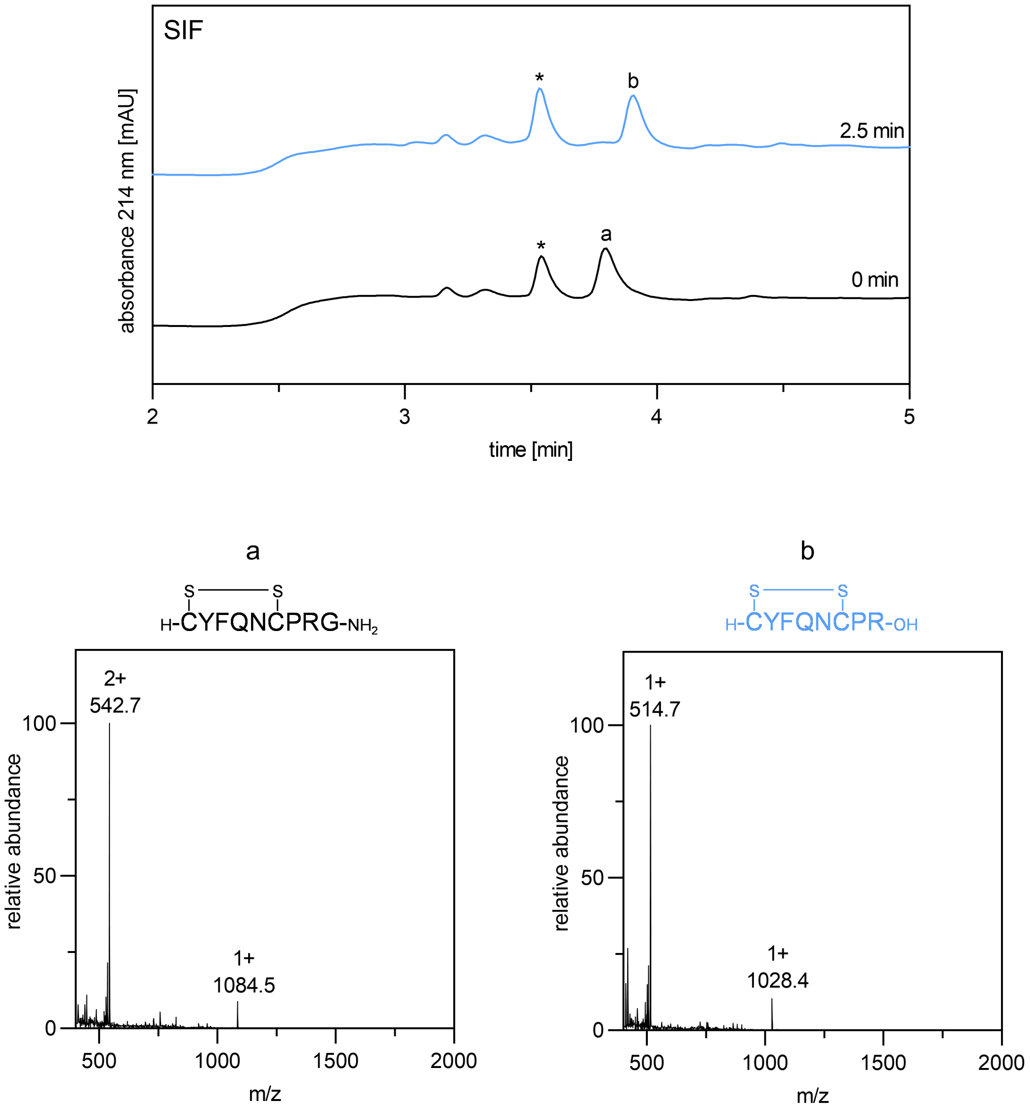

Figure S8: Primary metabolite of vasopressin in SIF. * depict SIF background peaks.

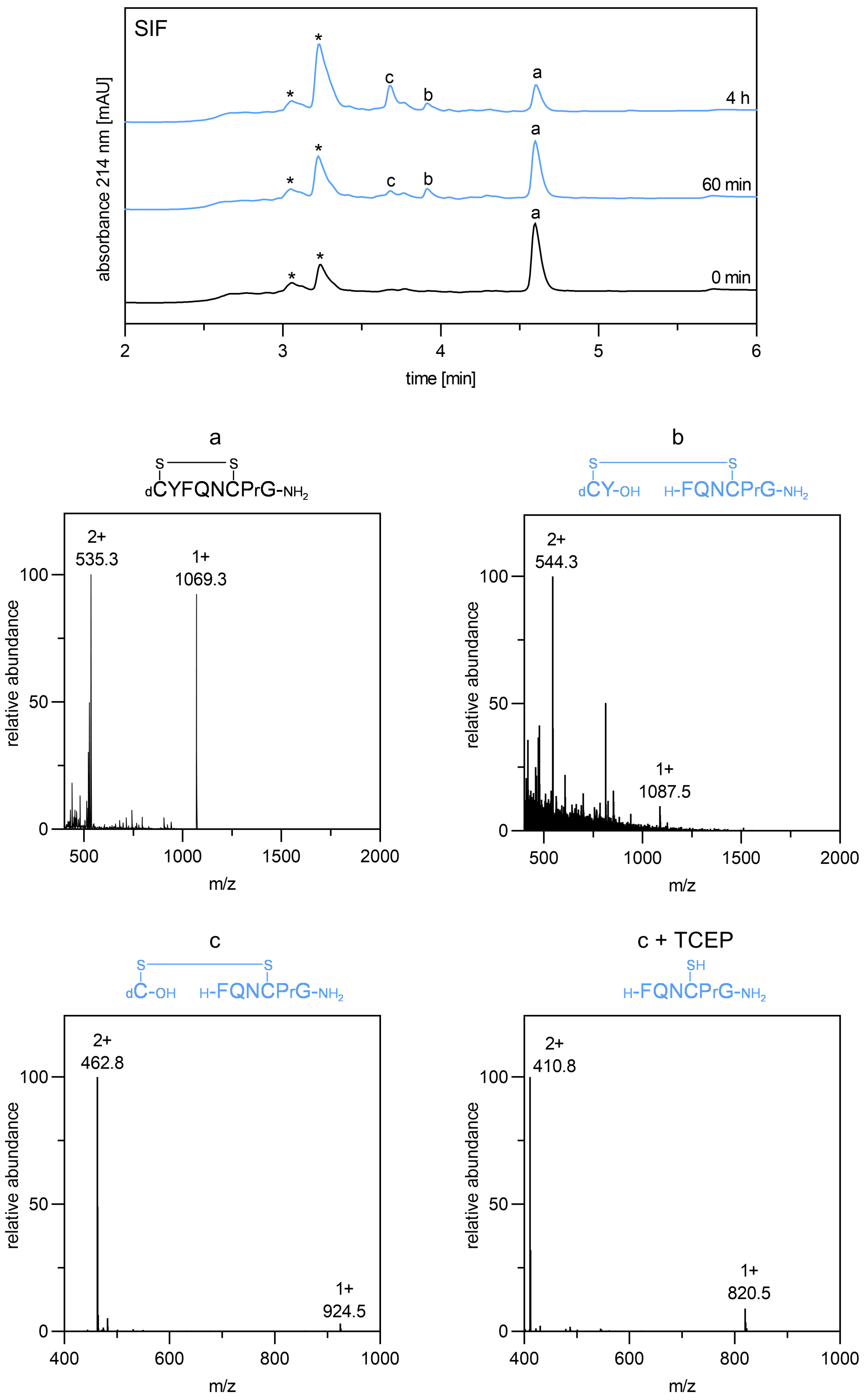

Figure S9: Primary metabolites of desmopressin in SIF. * depict SIF background peaks.

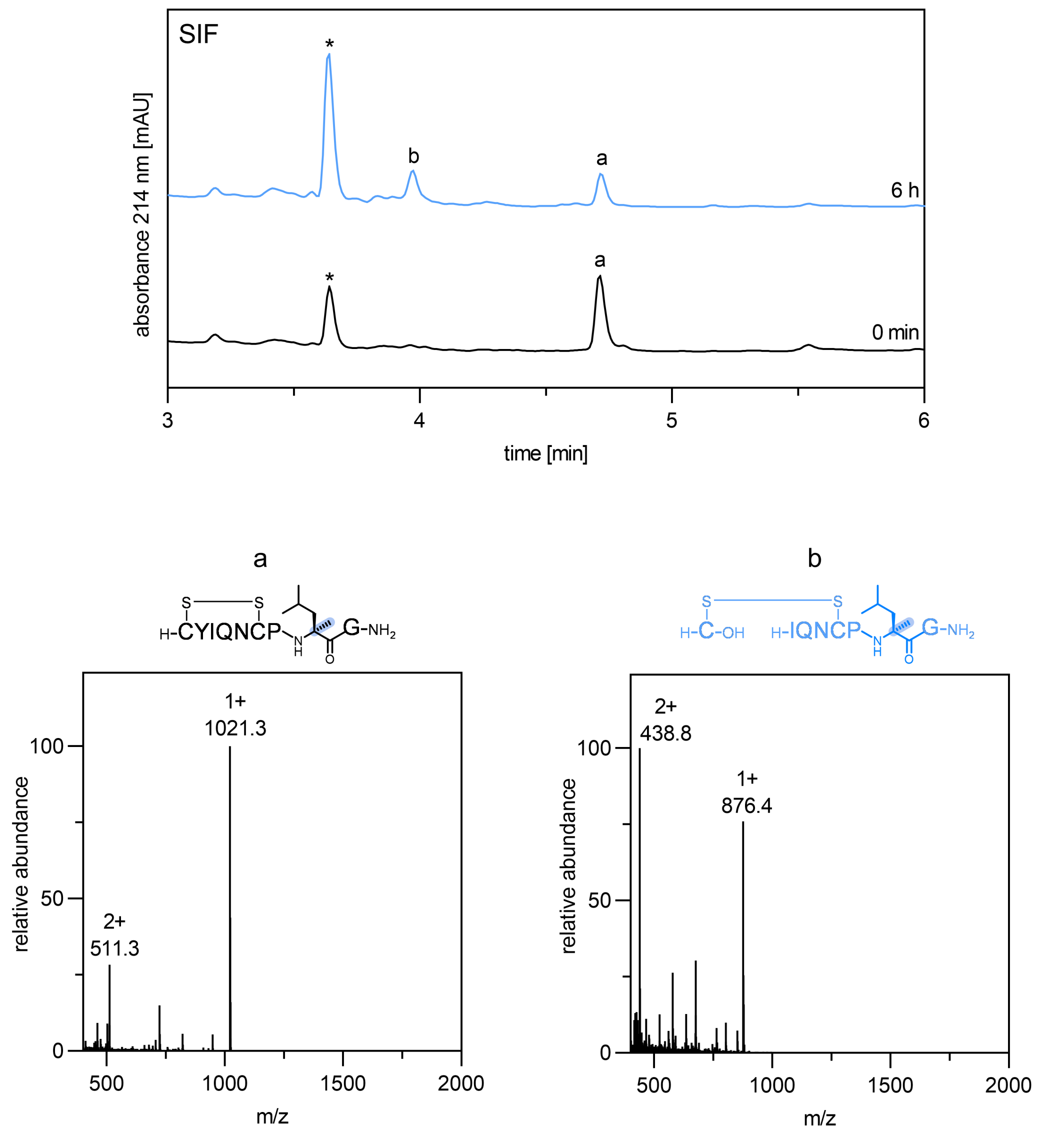

Figure S10: Primary metabolite of (Cα-Me)L⁸OT in SIF. * depict SIF background peaks.

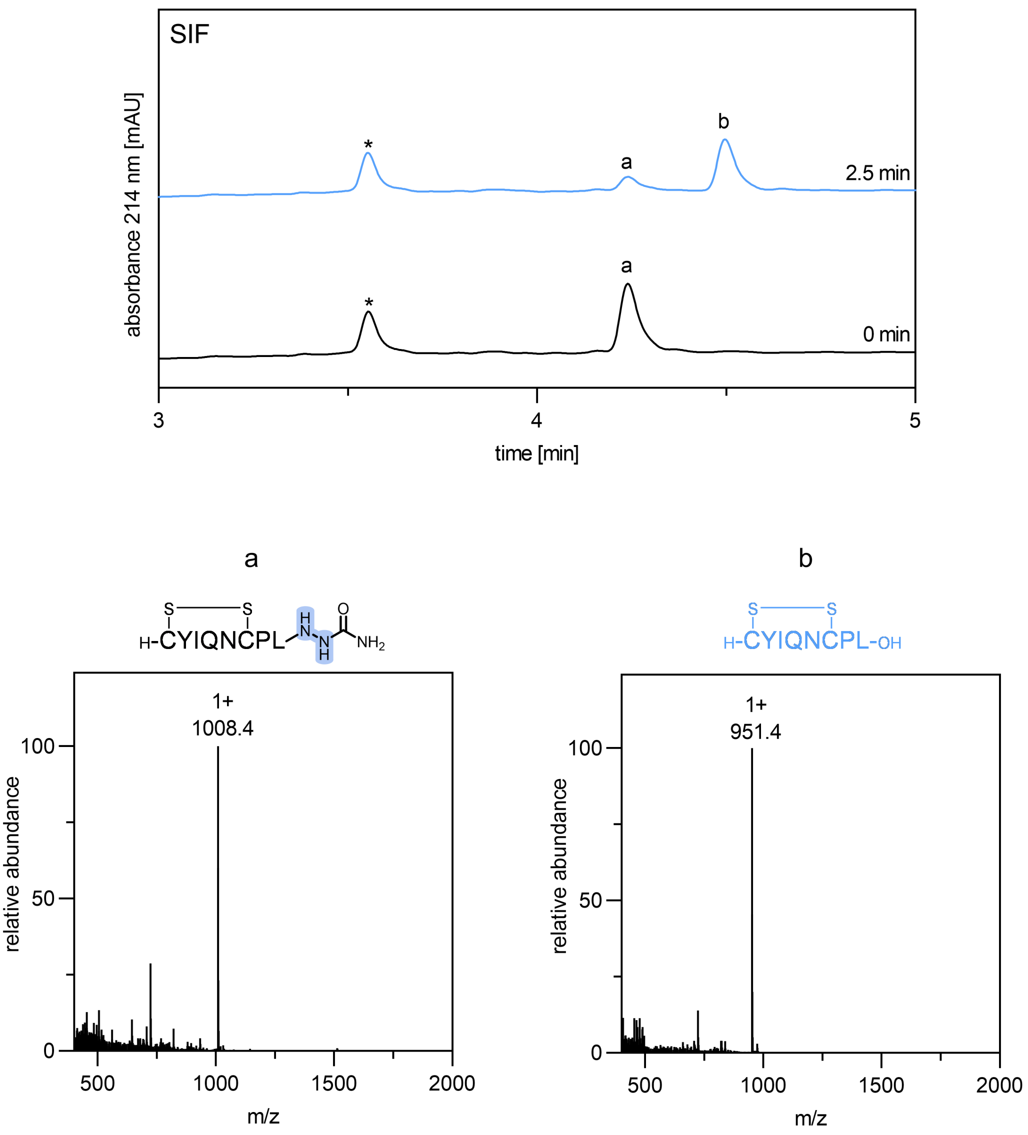

Figure S11: Primary metabolite of G^9(aza)OT in SIF. * depict SIF background peaks.

Summary of pharmacological data: OTR activity of OT variants

Activity at the human oxytocin receptor (OTR) was evaluated *via* fluorescent imaging plate reader (FLIPR) Ca^{2+}-mobilization assay (n ≥ 2) as recently desrcibed.[12] Activity data for (C_α-Me)L^8OT, G^9(aza)OT and all OT/VP-SFTI-1 -grafts was generously provided by the National Institute of Mental Health's Psychoactive Drug Screening Program (NIMH PDSP).[13] For analogues previously reported in the literature, activity data is cited for comparison. For better clarity of the activity data, the EC_{50} value of the control native OT is presented for each compound and assay in grey.

Table S1: Summarized OTR activity data of all synthesized OT variants.

Entry	Compound	EC_{50} ± SEM [nM]	E_{max} ± SEM [% OT]	OT EC_{50} ± SEM [nM]	Assay	Reference
1	Oxytocin	values individually given per assay/compound below				
2	all-D-retroinverse-OT	$>10^5$	-	15 ± 2.5	IP-1	14
3	(C_α-Me)L^8OT	3.80 ± 2.70	105 ± 11	3.91 ± 0.87	Ca^{2+}	
4	(β^3-homo)L^8OT	n.d.	n.d.			
5	(N_α-Me)G^9OT	0.47 ± 0.23	102 ± 2	0.04 ± 0.01	Ca^{2+}	15
6	G^9(aza)OT	2.24 ± 0.63	111 ± 6	3.91 ± 0.87	Ca^{2+}	
7	c(OT)	$>10^5$	-	15 ± 2.5	IP-1	14
8	c(linker-OT)	$>10^5$	-	15 ± 2.5	IP-1	14
9	$c(OT)_2$	$>10^4$	-	13.4 ± 6.4	IP-1	12
10	c(OT-VP)	$>10^4$	-	13.4 ± 6.4	IP-1	12
11	$c(VP)_2$	$>10^4$	-	13.4 ± 6.4	IP-1	12
12	KB1($OT^{[2-5]}$)	$>10^4$	-	5.7 ± 4.0	Ca^{2+}	
13	KB1($T^4G^7OT^{[2-5]}$)	$>10^4$	-	5.7 ± 4.0	Ca^{2+}	
14	KB7($T^4G^7OT^{[2-5]}$)	$>10^4$	-	5.7 ± 4.0	Ca^{2+}	
15	SFTI-1($OT^{[2-5]}$)	$>3x10^4$	-	12.6 ± 1.28	Ca^{2+}	
16	SFTI-1($OT^{[2-9]}$)	$>3x10^4$	-	12.6 ± 1.28	Ca^{2+}	
17*	SFTI-1($VP^{[2-5]}$)	$>3x10^4$	-	12.6 ± 1.28	Ca^{2+}	
18*	SFTI-1($VP^{[2-9]}$)	$>3x10^4$	-	12.6 ± 1.28	Ca^{2+}	
19*	SFTI-1($Anta^{V1aR}$)	$>3x10^4$	-	12.6 ± 1.28	Ca^{2+}	

IP-1: inositol 1 phosphate accumulation assay. Ca^{2+}: FLIPR Ca^{2+}-mobilization assay. n.d.: not determined.
**Vasopressin (VP) grafts of SFTI-1 were also evaluated on $V_{1a}R$, $V_{1b}R$ and V_2R functional activity under the same assay conditions but no agonistic (EC_{50} > 3 x 10^4 nM) or antagonist properties was detected.*

Full gut stability curves and final analysis of tested compounds

Gut stability results are presented as mean ± SEM of n ≥ 3 independent experiments. Note: some error bars are smaller than the symbols.

Final analytical HPLC chromatograms were recorded on a Thermo Scientific Vanquish Horizon UHPLC system with UV detection at 214 and 280 nm. The analysis was performed on a Kromasil Classic C_{18} column (4.6 × 150 mm, 300 Å, 5 µm) using a flow rate of 1 mL/min and linear gradient elution of 5-65% solvent B in 30 min at 30 °C. Solvent A: 0.1% TFA in ddH_2O, solvent B: 0.08% TFA in ACN.

Final high-resolution (HR)-MS analysis was performed on a Thermo Scientific LTQ Orbitrap Velos mass spectrometer coupled to a Thermo Scientific Vanquish Horizon UHPLC system. Samples were analyzed in LC-MS mode using an Acclaim C_{18} HPLC column (2.1 × 150 mm, 120 Å, 3 µm, Thermo Fisher Scientific) and the following chromatographic parameters: linear gradient elution of 10-65% solvent B in 14 min, flow rate of 0.45 mL/min at 30 °C. Solvent A: 0.1% formic acid in ddH_2O, solvent B: 0.1% formic acid in ACN. HR-ESI-MS spectra were recorded in positive ion mode in the range of m/z 300-2000 with an FT resolution of 60.000. The sum formulas of the detected ions were confirmed using Xcalibur 4.2.47 based on the mass accuracy (Δm/z ≤ 5 ppm) and isotopic pattern. Observed compound masses (M_{obs}) were manually calculated from the base peak of each spectrum and are stated as such for all compounds.

Kalata B1

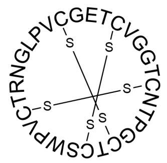
GLPVCGETCVGGTCNTPGCTCSWPVCTRN
S
S
S
S
S
S

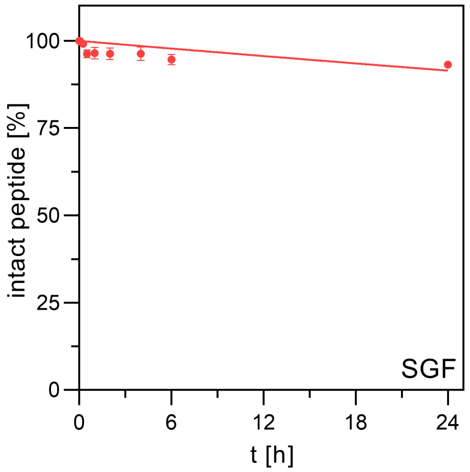
intact peptide [%]
100
75
50
25
0
0
6
12
18
24
t [h]
SGF

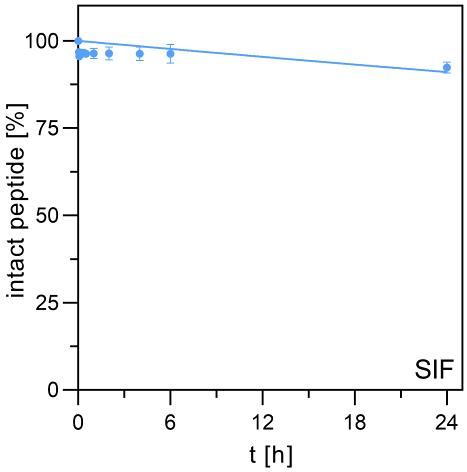
intact peptide [%]
100
75
50
25
0
0
6
12
18
24
t [h]
SIF

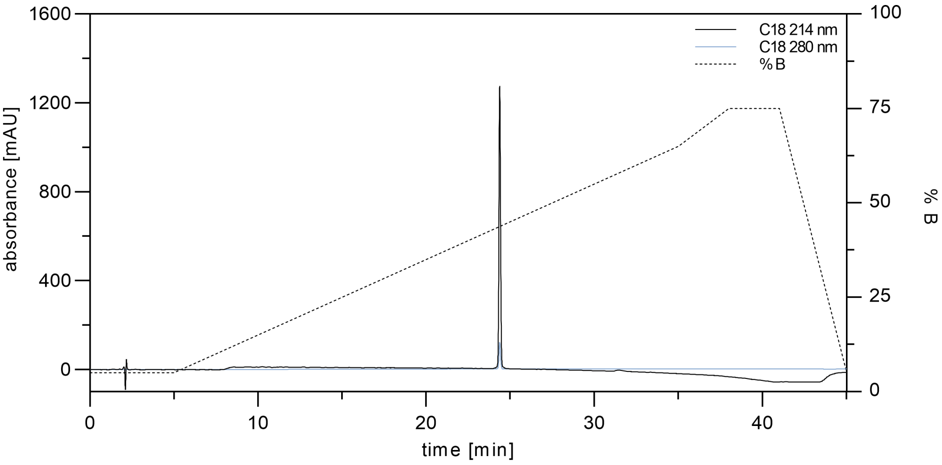
absorbance [mAU]
1600
1200
800
400
0
C18 214 nm
C18 280 nm
% B
100
75
50
25
0
% B
0
10
20
30
40
time [min]

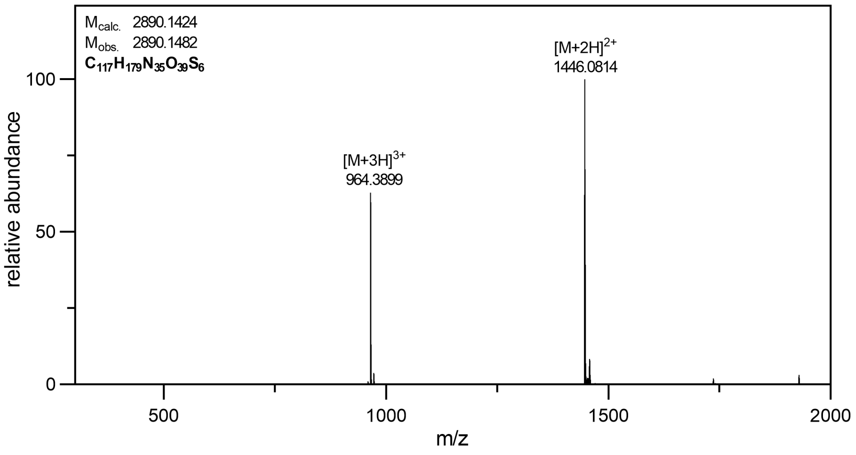
$M_{calc.}$ 2890.1424
$M_{obs.}$ 2890.1482
$C_{117}H_{179}N_{35}O_{39}S_6$
$[M+2H]^{2+}$
1446.0814
$[M+3H]^{3+}$
964.3899
relative abundance
100
50
0
500
1000
1500
2000
m/z

Kalata B7

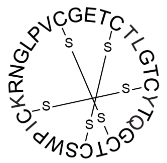
KRNGLPVCGETCTLGTCYTQGCTCSWPIC

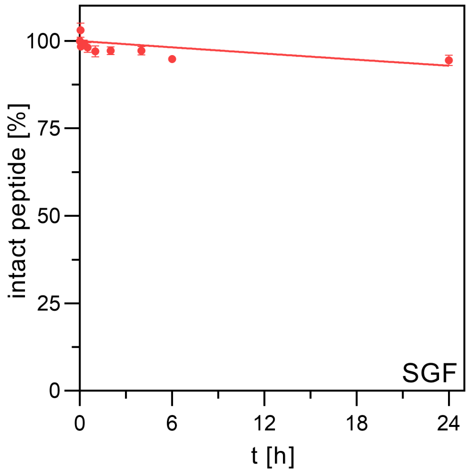
intact peptide [%]
t [h]
SGF

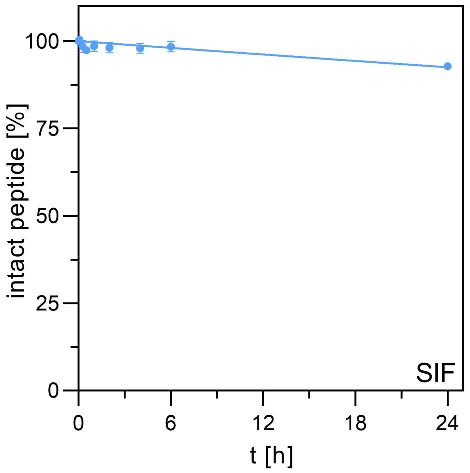
intact peptide [%]
t [h]
SIF

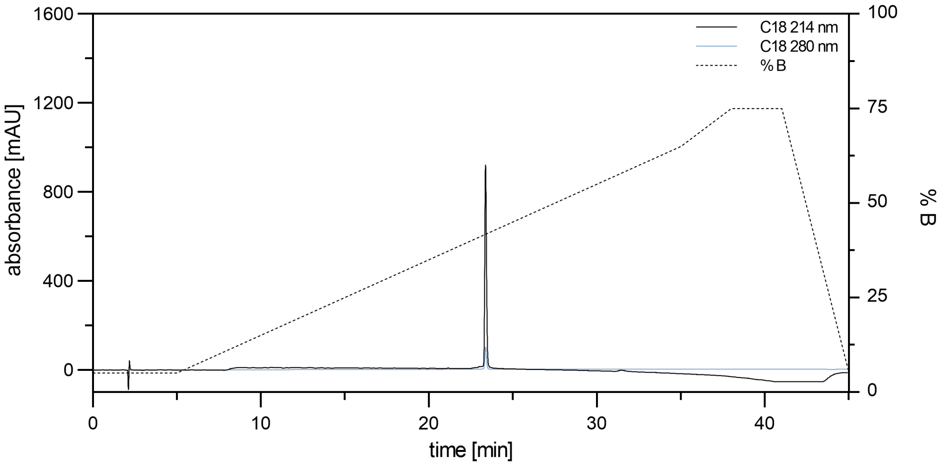
C18 214 nm
C18 280 nm
% B
absorbance [mAU]
time [min]
% B

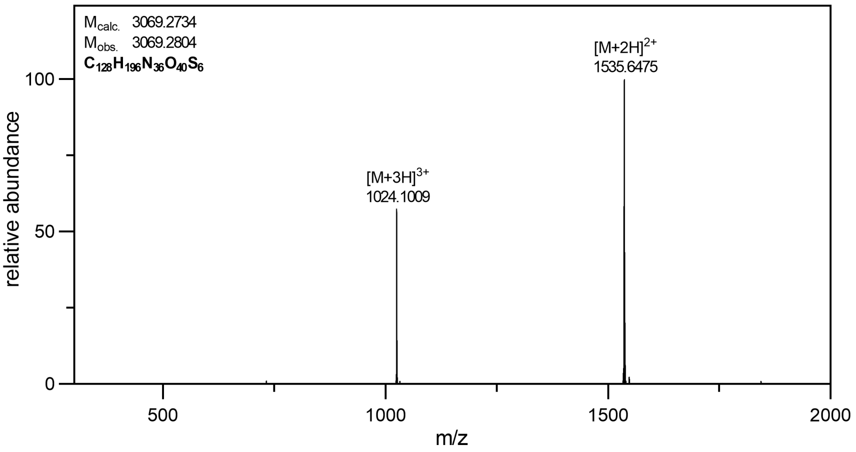
$M_{calc.}$ 3069.2734
$M_{obs.}$ 3069.2804
$C_{128}H_{196}N_{36}O_{40}S_6$
$[M+2H]^{2+}$
1535.6475
$[M+3H]^{3+}$
1024.1009
relative abundance
m/z

SFTI-1 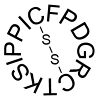

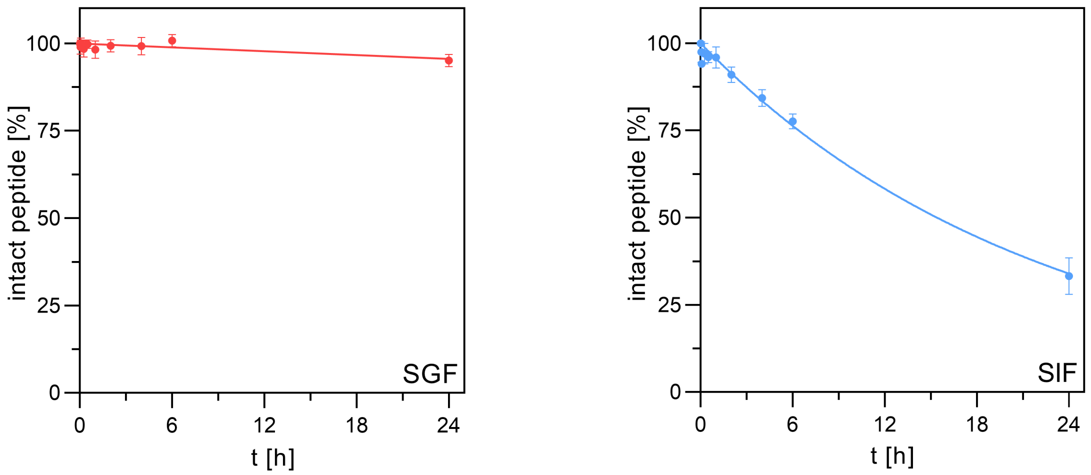
intact peptide [%]
t [h]
SGF
SIF

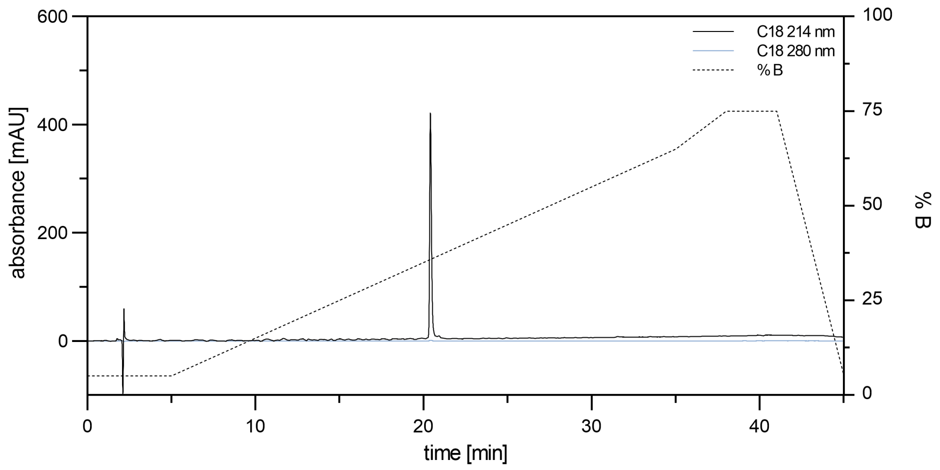
absorbance [mAU]
time [min]
% B
C18 214 nm
C18 280 nm
% B

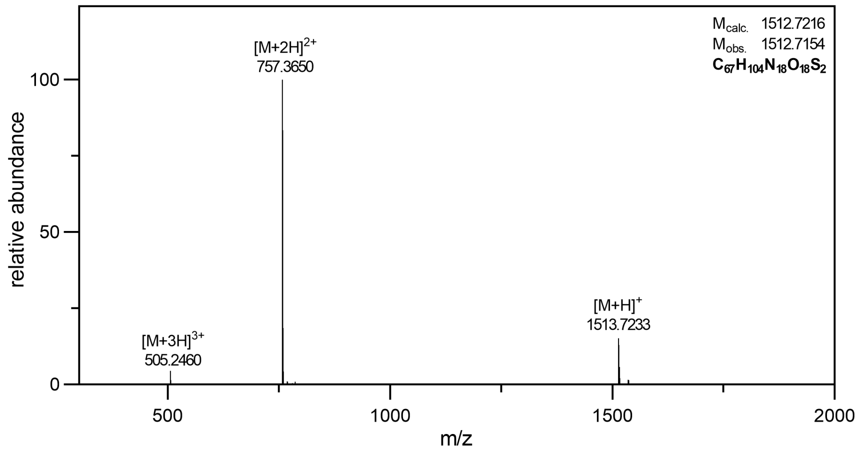
relative abundance
m/z
$M_{calc.}$ 1512.7216
$M_{obs.}$ 1512.7154
$C_{67}H_{104}N_{18}O_{18}S_2$
$[M+2H]^{2+}$ 757.3650
$[M+3H]^{3+}$ 505.2460
$[M+H]^{+}$ 1513.7233

Vc1.1

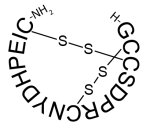
GCCSDPRCNYDHPEIC-NH2

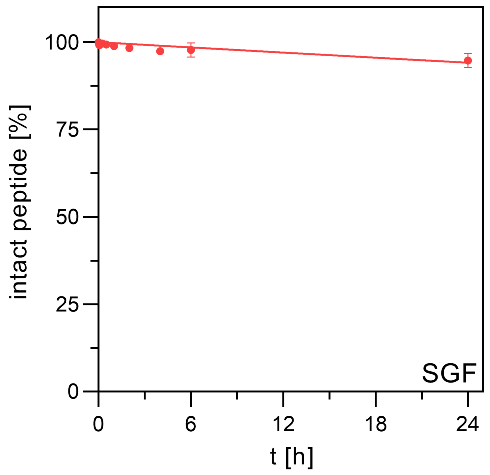
intact peptide [%]
t [h]
SGF

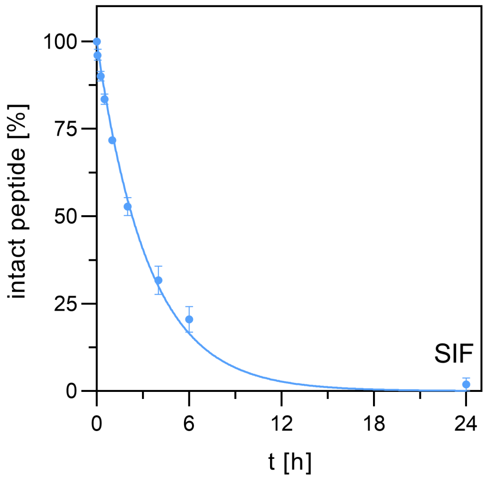
intact peptide [%]
t [h]
SIF

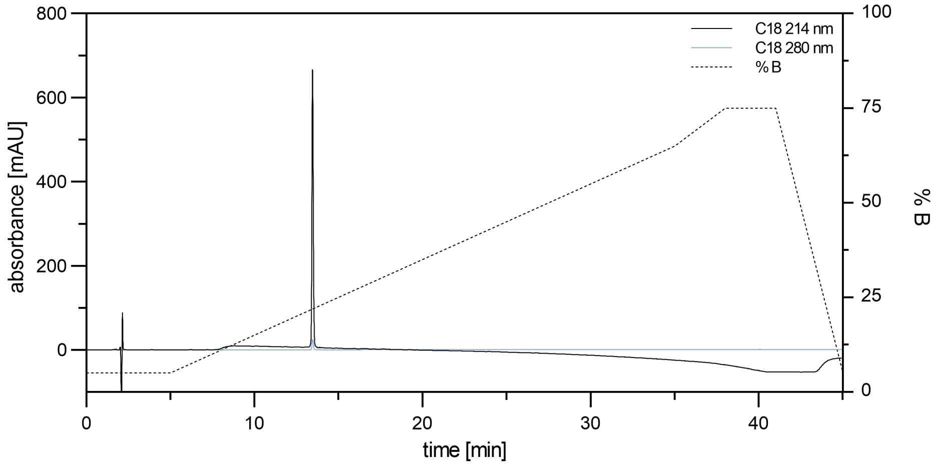
C18 214 nm
C18 280 nm
% B
absorbance [mAU]
time [min]
% B

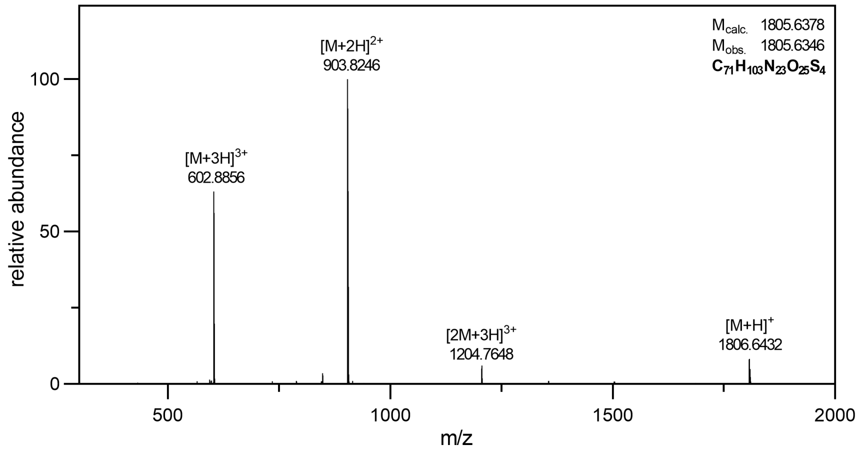
M calc. 1805.6378
M obs. 1805.6346
C71H103N23O25S4
[M+2H]2+
903.8246
[M+3H]3+
602.8856
[2M+3H]3+
1204.7648
[M+H]+
1806.6432
relative abundance
m/z

cVc1.1

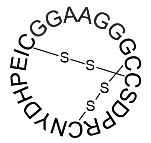
GGAAGGCCSDPRCNYDHPEIC
S–S
S–S

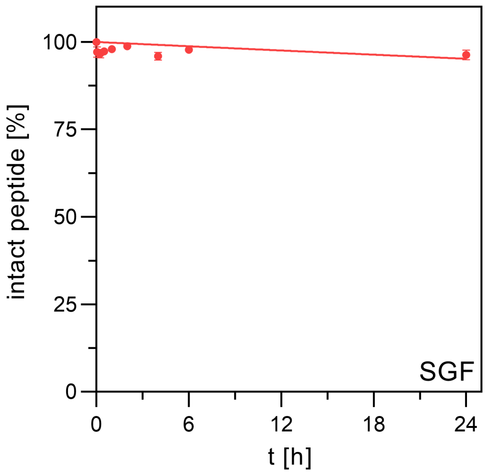
intact peptide [%]
100
75
50
25
0
0
6
12
18
24
t [h]
SGF

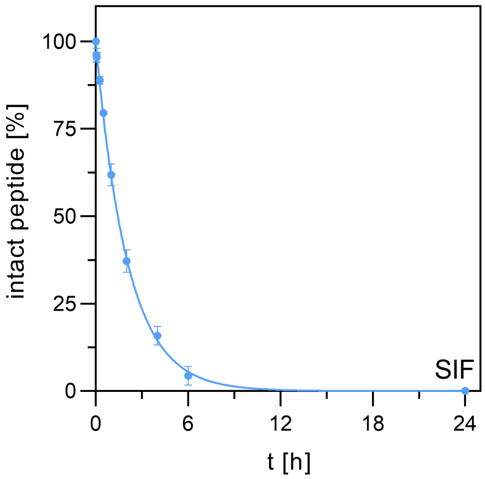
intact peptide [%]
100
75
50
25
0
0
6
12
18
24
t [h]
SIF

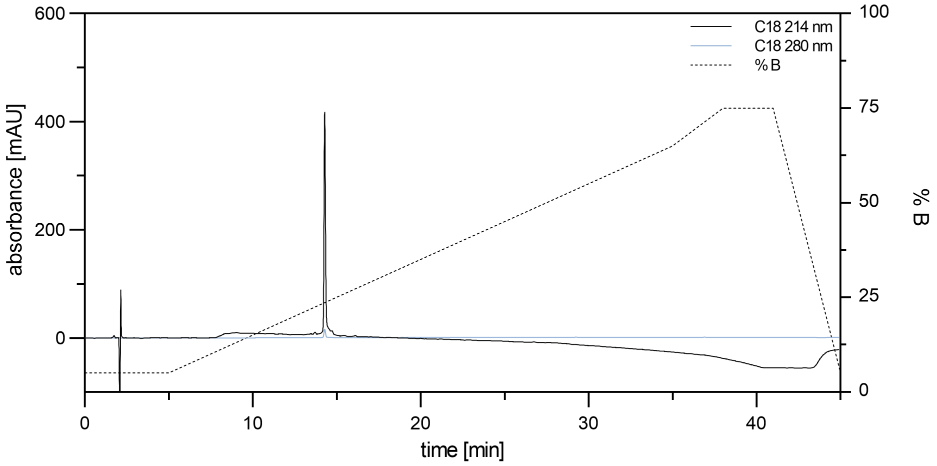
absorbance [mAU]
600
400
200
0
0
10
20
30
40
time [min]
100
75
50
25
0
% B
C18 214 nm
C18 280 nm
% B

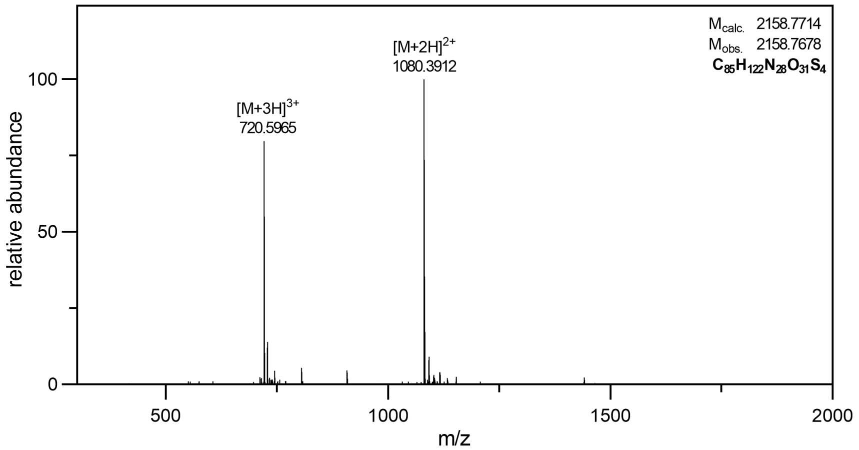
relative abundance
100
50
0
500
1000
1500
2000
m/z
[M+3H]3+
720.5965
[M+2H]2+
1080.3912
Mcalc. 2158.7714
Mobs. 2158.7678
C85H122N28O31S4

RTD-1

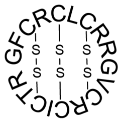
GFCRCLCRRGVCRCICTR
S S S S S S

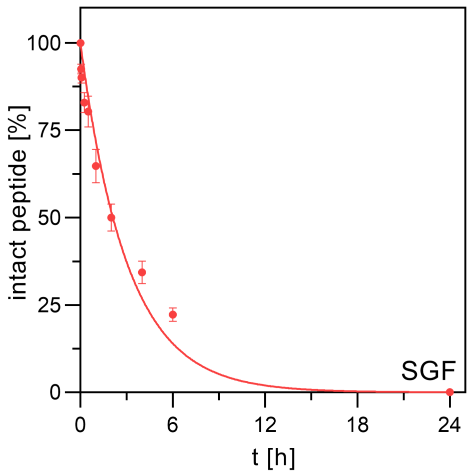
intact peptide [%]
100
75
50
25
0
0
6
12
18
24
t [h]
SGF

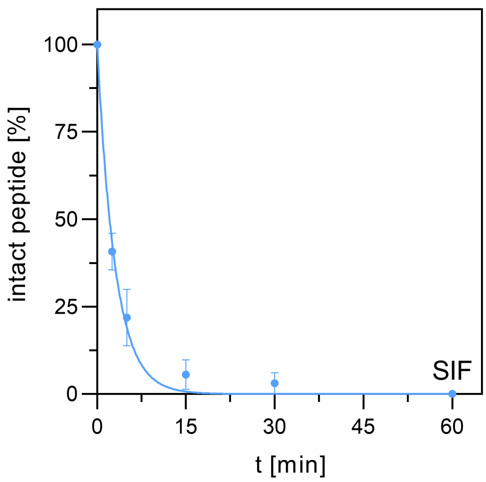
intact peptide [%]
100
75
50
25
0
0
15
30
45
60
t [min]
SIF

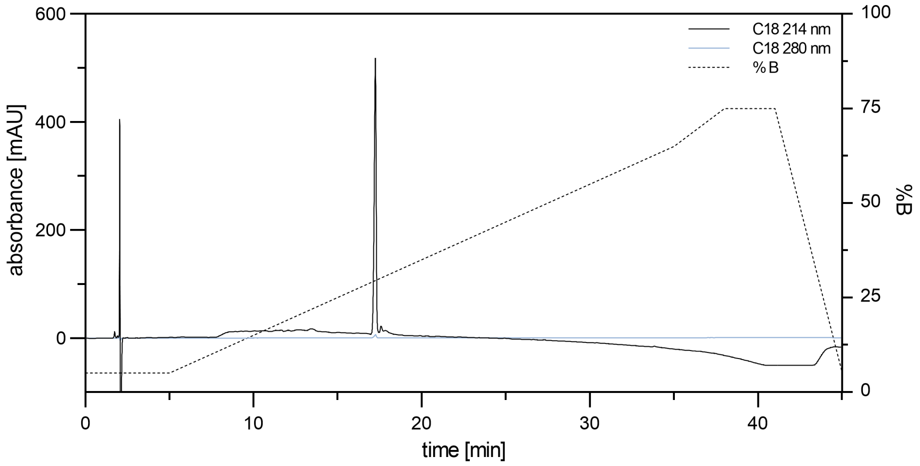
C18 214 nm
C18 280 nm
% B
absorbance [mAU]
600
400
200
0
0
10
20
30
40
time [min]
100
75
50
25
0
%B

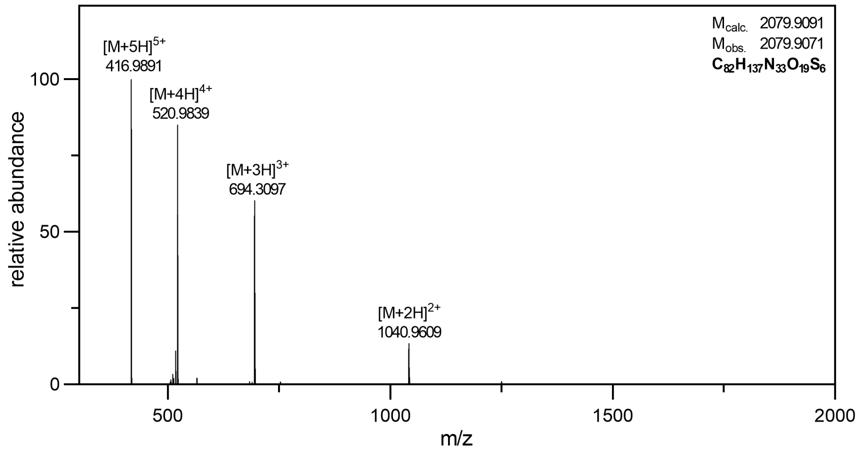
$M_{calc.}$ 2079.9091
$M_{obs.}$ 2079.9071
$C_{82}H_{137}N_{33}O_{19}S_6$
$[M+5H]^{5+}$
416.9891
$[M+4H]^{4+}$
520.9839
$[M+3H]^{3+}$
694.3097
$[M+2H]^{2+}$
1040.9609
relative abundance
100
50
0
500
1000
1500
2000
m/z

Linaclotide

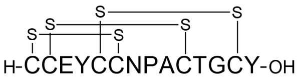

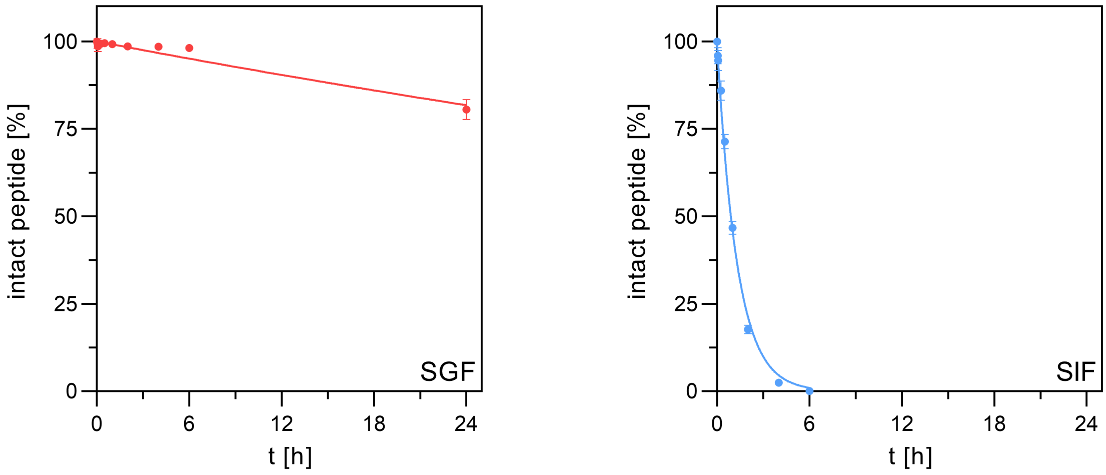

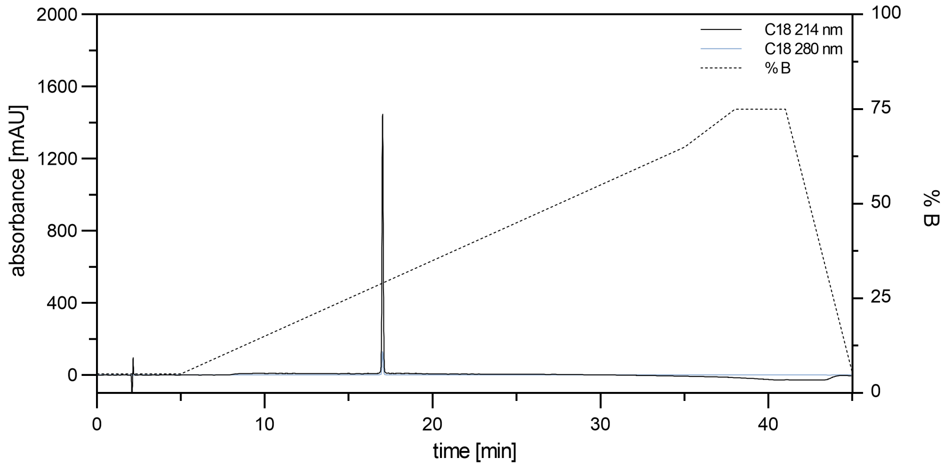

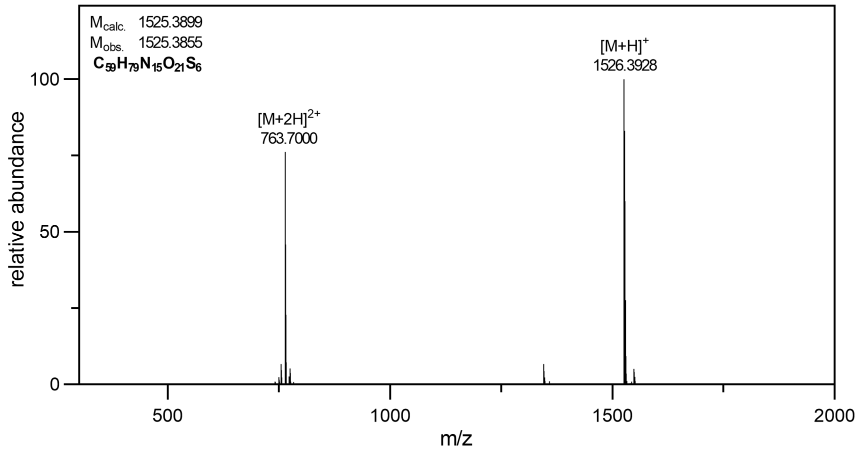

Plecanatide

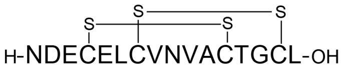

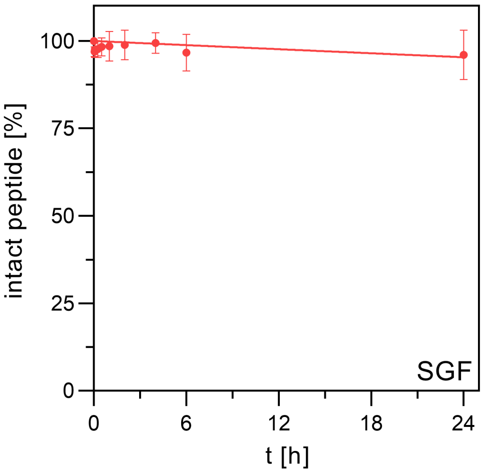

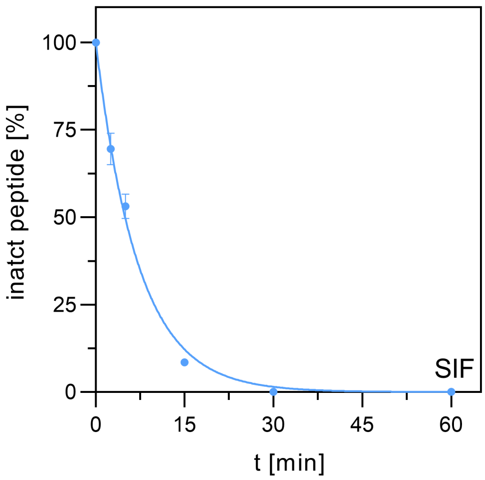

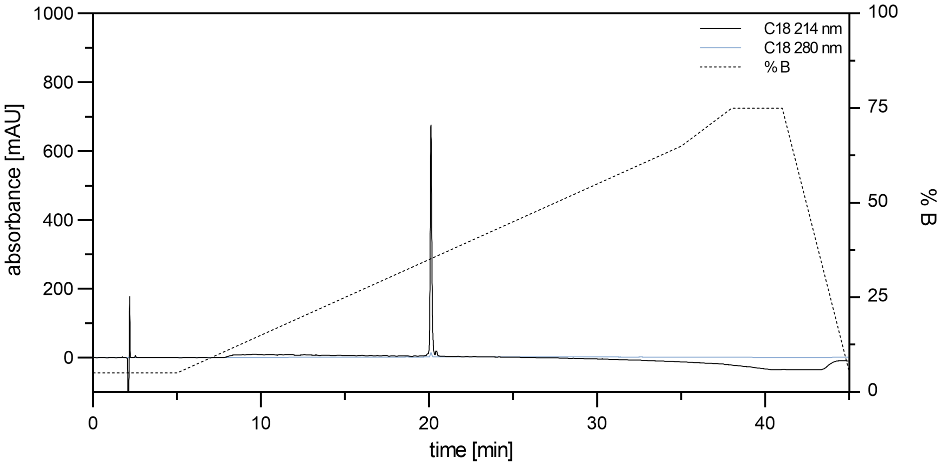

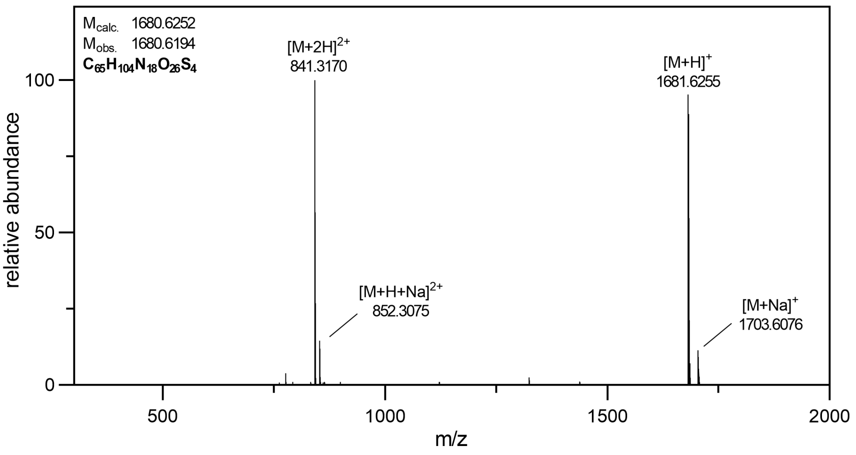

Dolcanatide

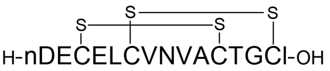

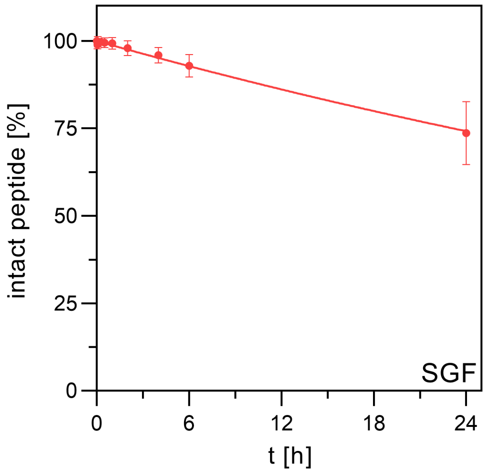

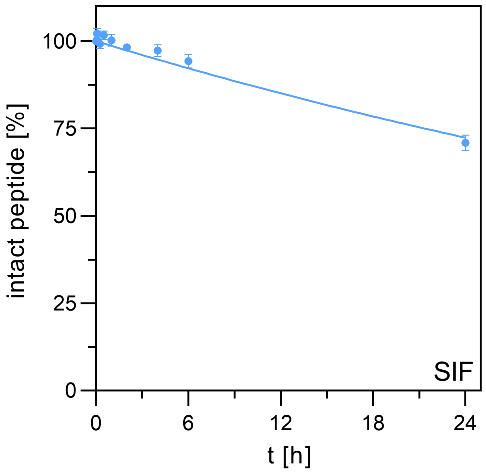

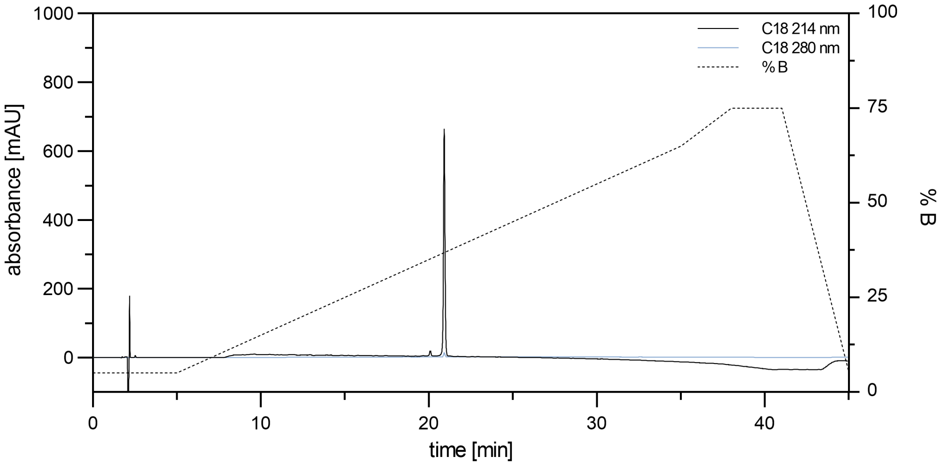

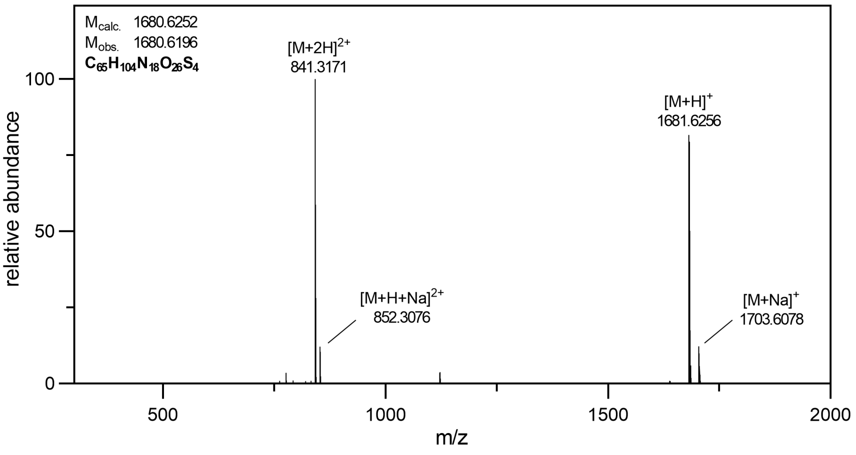

Somatostatin

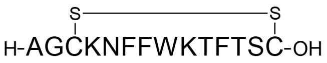

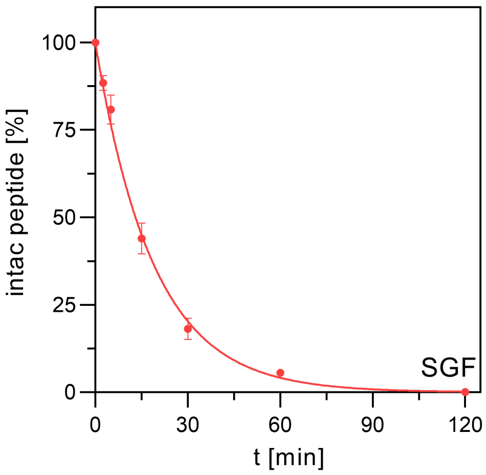

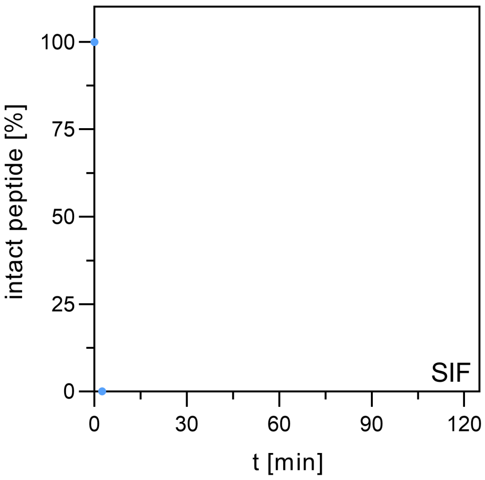

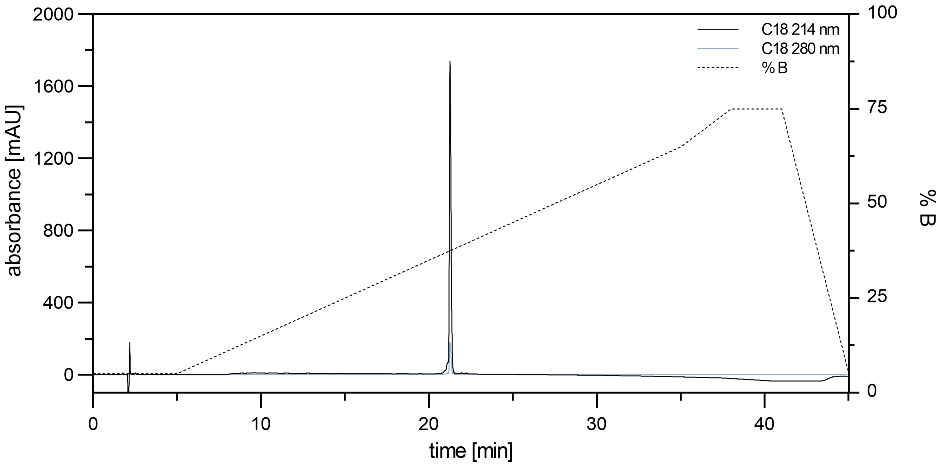

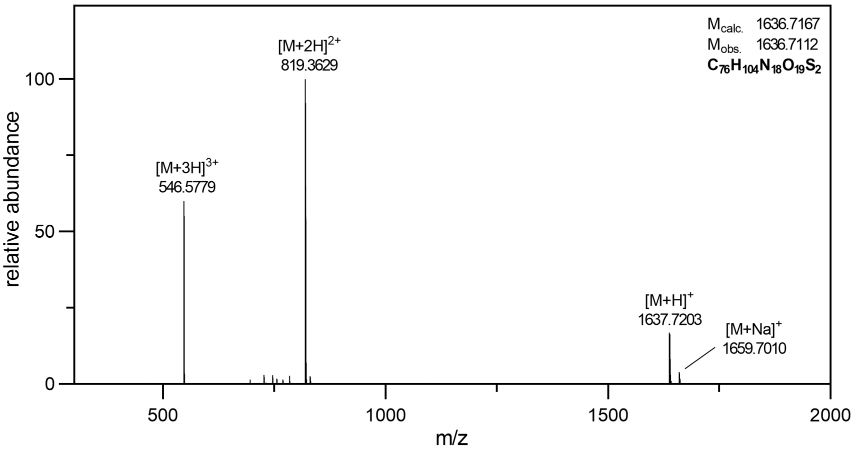

Note: Somatostatin was rapidly degraded in SIF with no intact compound detectable at the first sampling time point (2.5 min). The illustrated graph shows t_0 (100%) and $t_{2.5min}$ (0%) without any degradation curve fit.

Octreotide

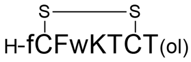

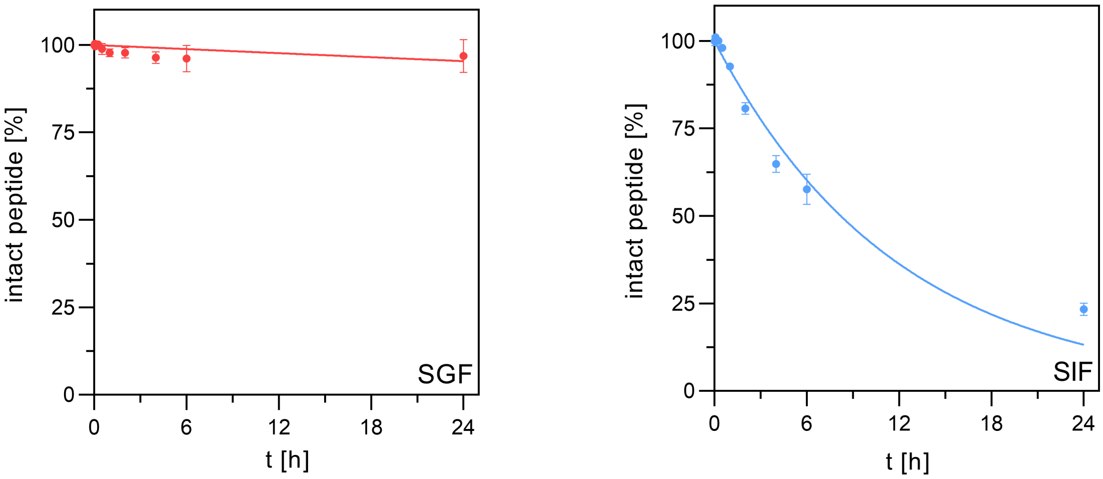

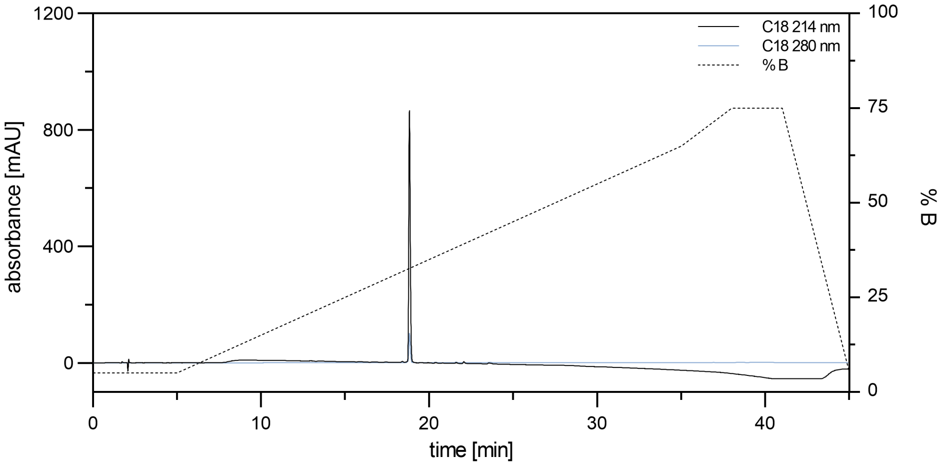

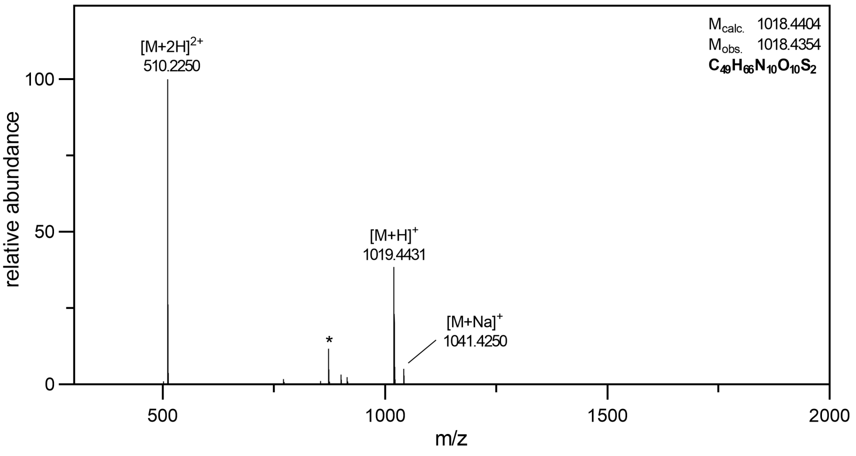

Note: *m/z 872.3757 depicts a source induced fragmentation ion.

Oxytocin H-CYIQNCPLG-NH_2 (S–S)

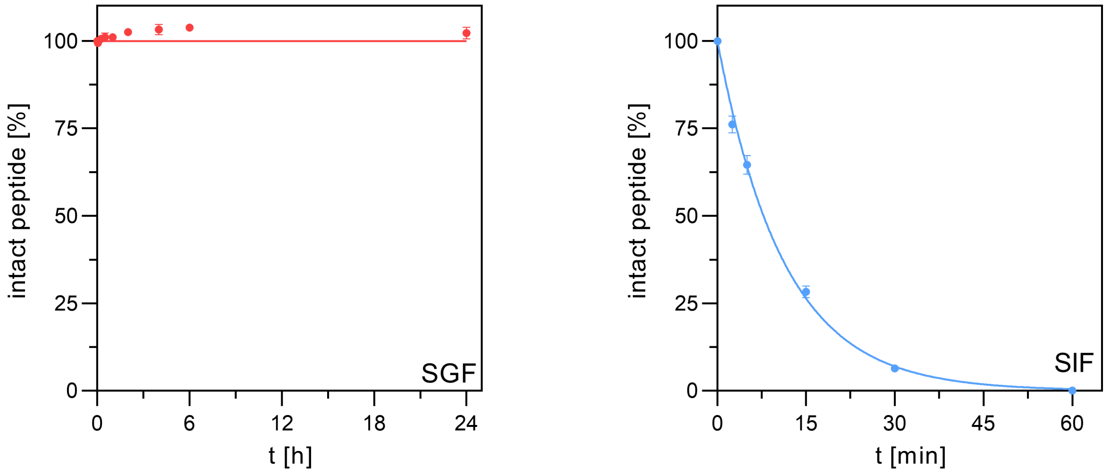

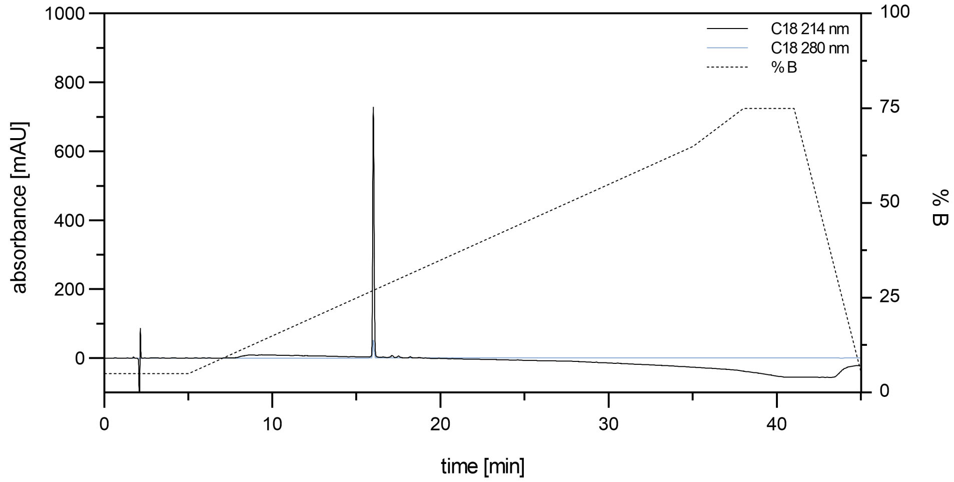

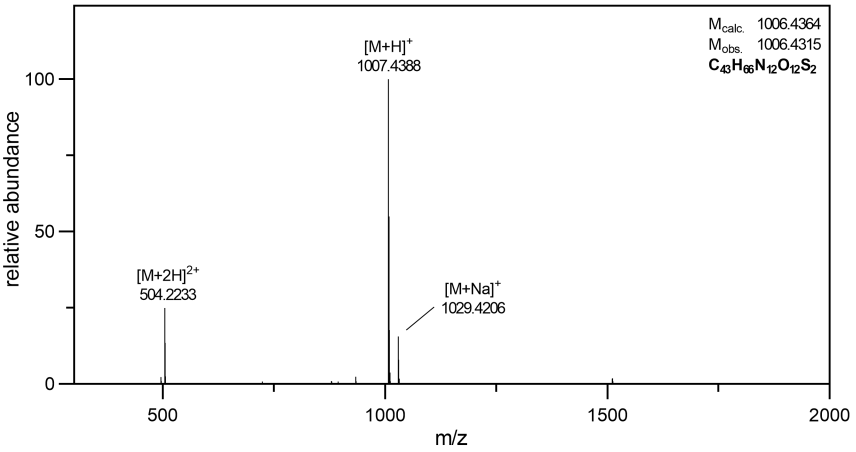

Carbetocin

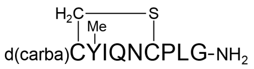

H2C
Me
S
d(carba)CYIQNCPLG-NH2

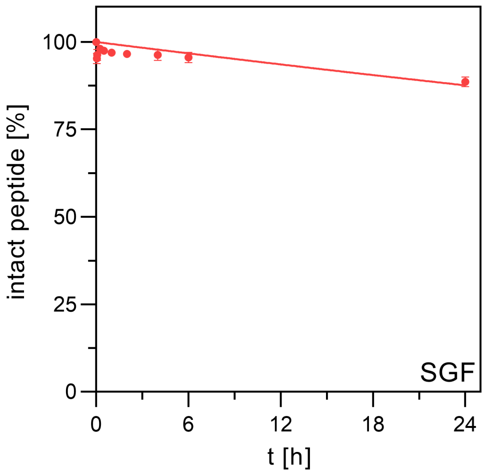

intact peptide [%]
t [h]
SGF

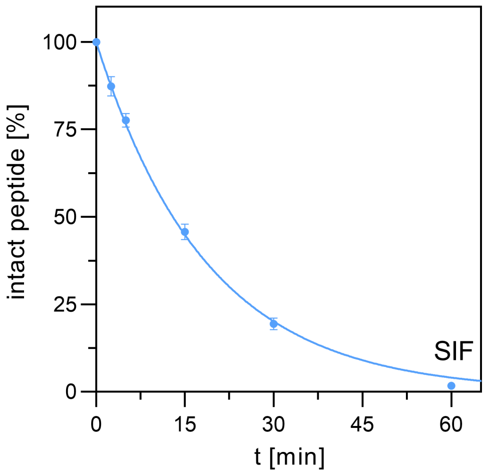

intact peptide [%]
t [min]
SIF

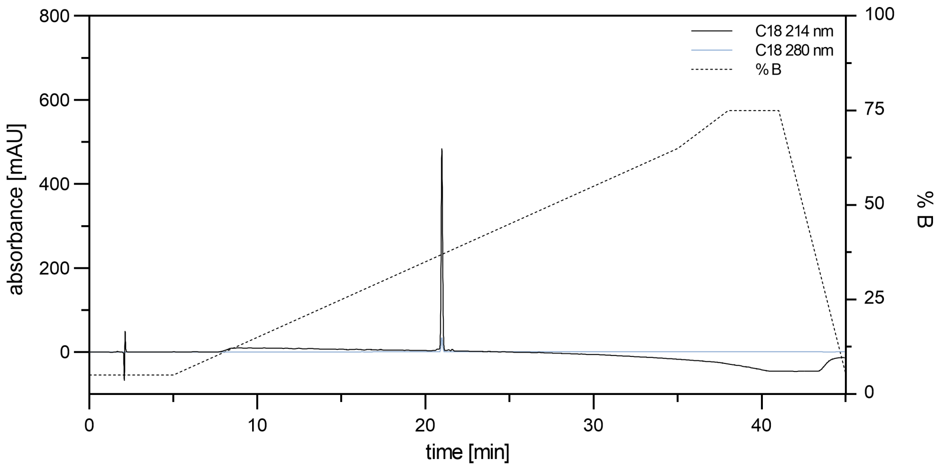

C18 214 nm
C18 280 nm
% B
absorbance [mAU]
time [min]
% B

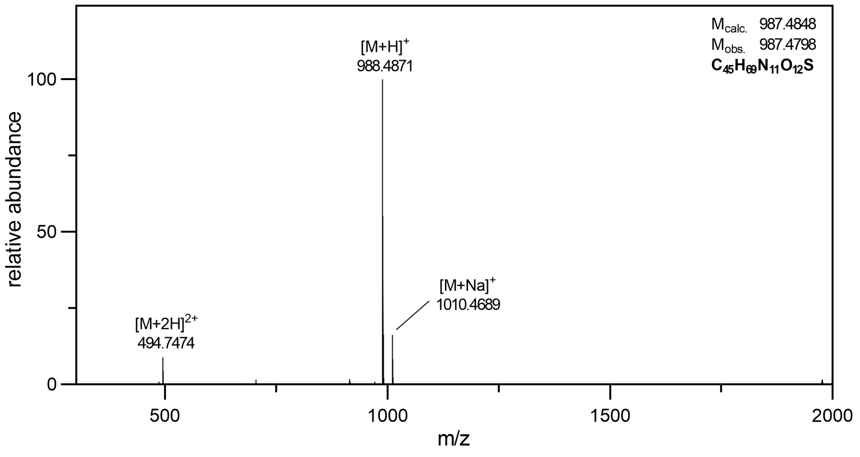

Mcalc. 987.4848
Mobs. 987.4798
C45H69N11O12S
[M+H]+
988.4871
[M+Na]+
1010.4689
[M+2H]2+
494.7474
relative abundance
m/z

Vasopressin

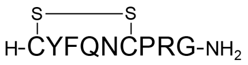

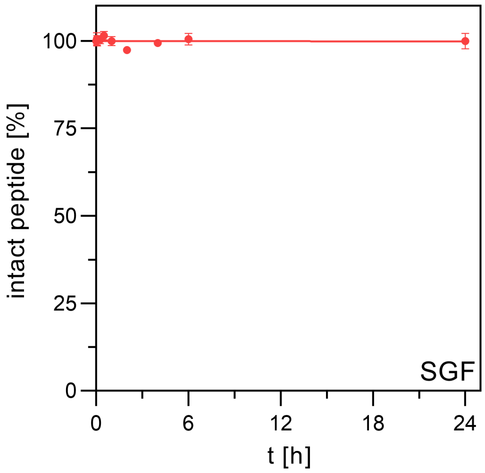

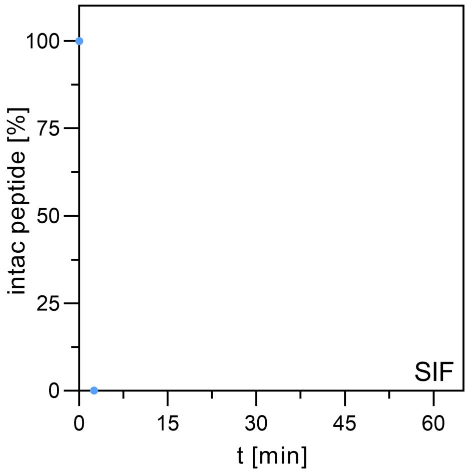

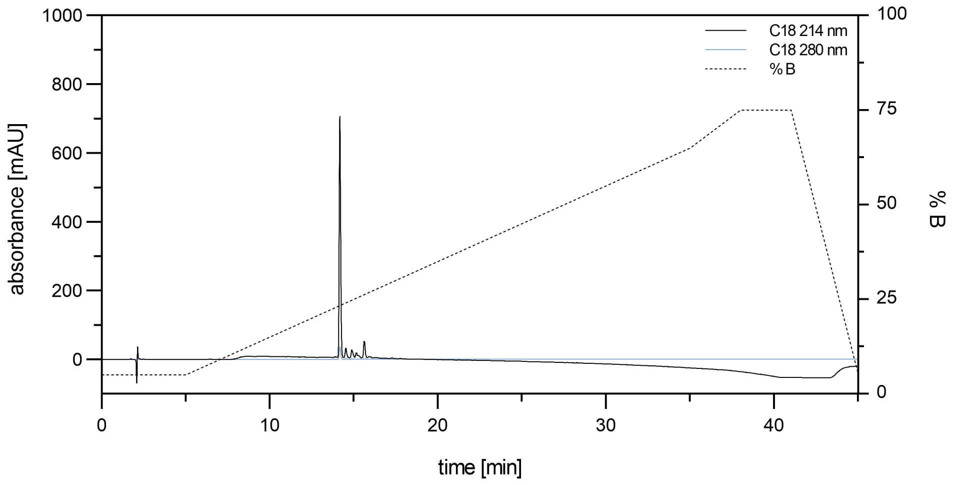

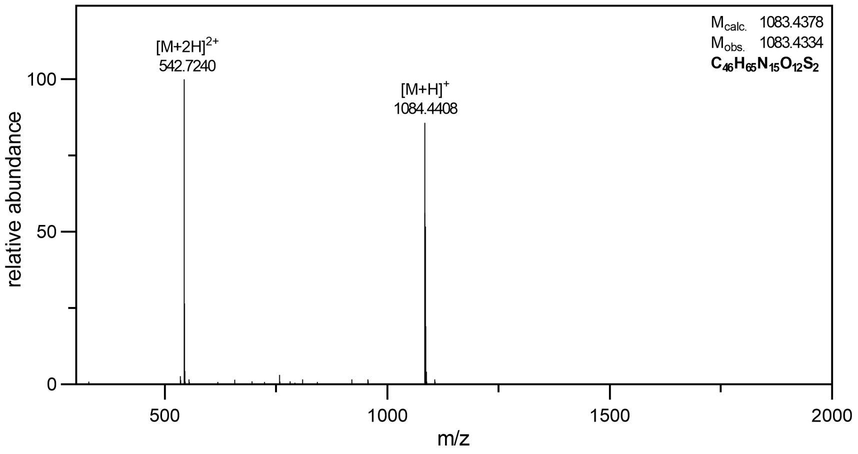

Note: Vasopressin was rapidly degraded in SIF with no intact compound detectable at the first sampling time point (2.5 min). The illustrated graph shows t_0 (100%) and $t_{2.5min}$ (0%) without any degradation curve fit.

Desmopressin dCYFQNCPrG-NH$_2$ (S–S bridge between the two C residues)

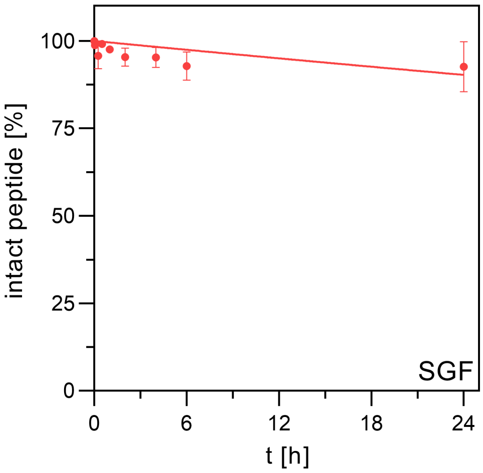

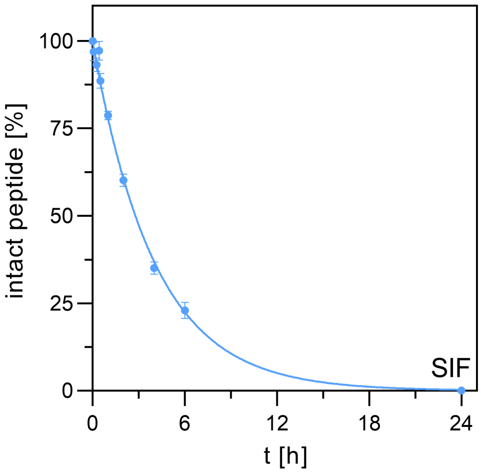

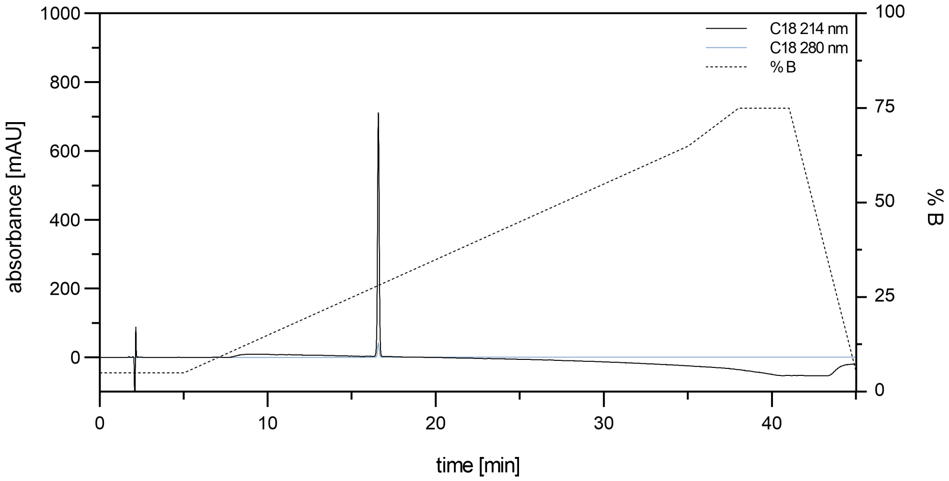

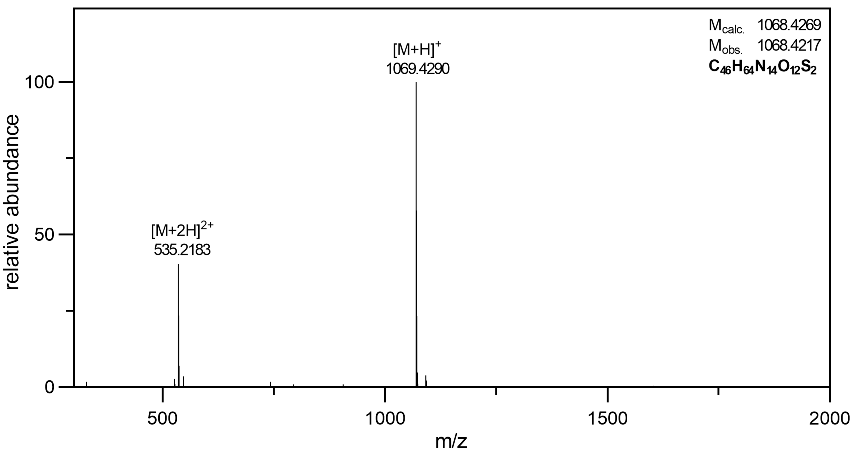

all-D-retroinverse-OT

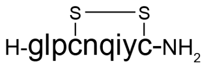

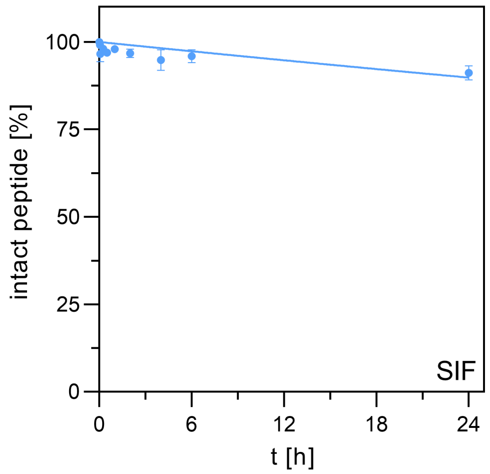

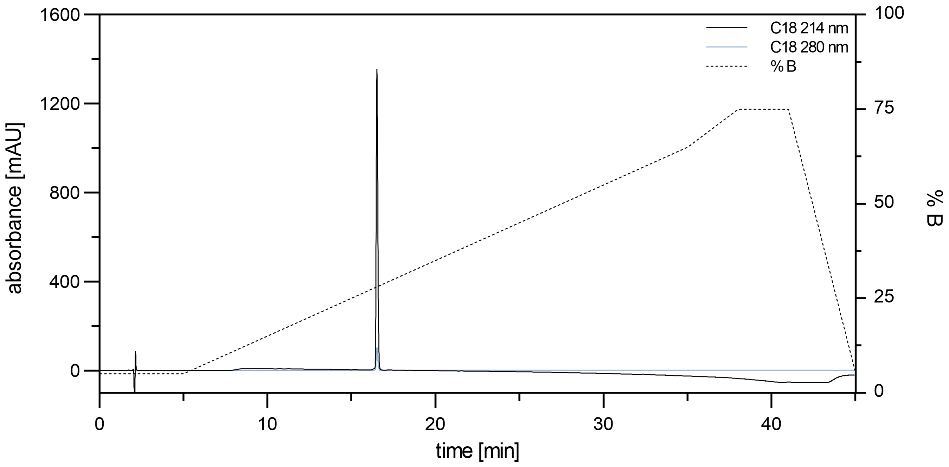

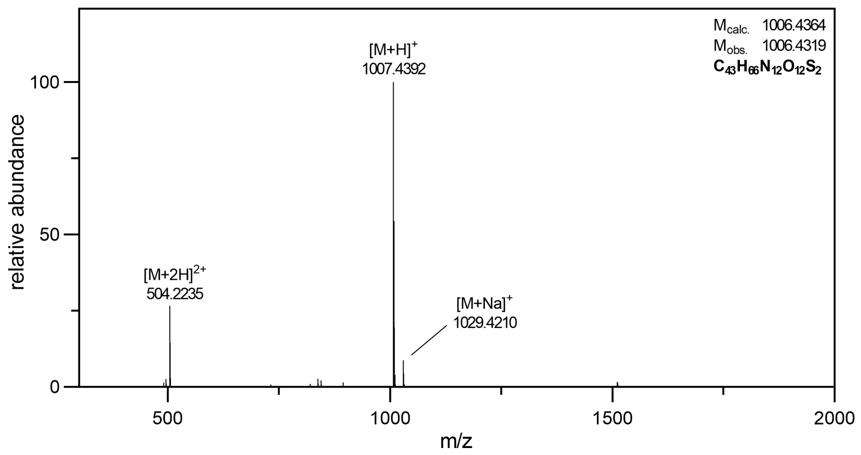

(Cα-Me)L[8]OT

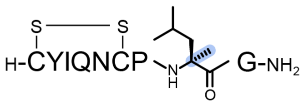
S
S
H-CYIQNCP
N
H
O
G-NH2

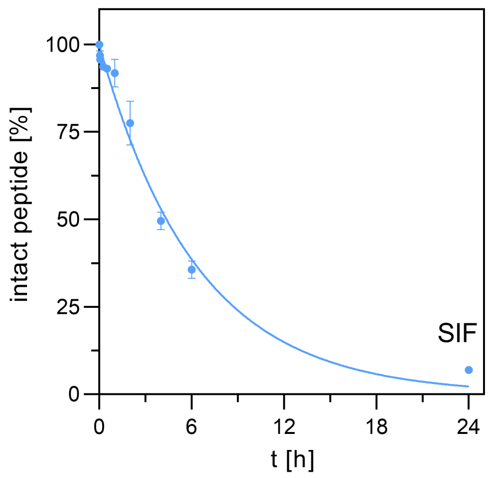
intact peptide [%]
t [h]
SIF
100
75
50
25
0
0
6
12
18
24

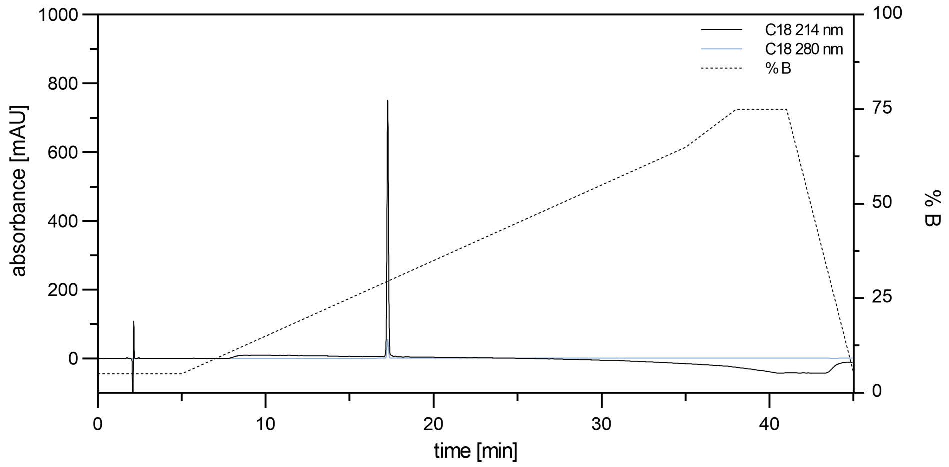
absorbance [mAU]
time [min]
% B
C18 214 nm
C18 280 nm
% B
1000
800
600
400
200
0
0
10
20
30
40
100
75
50
25
0

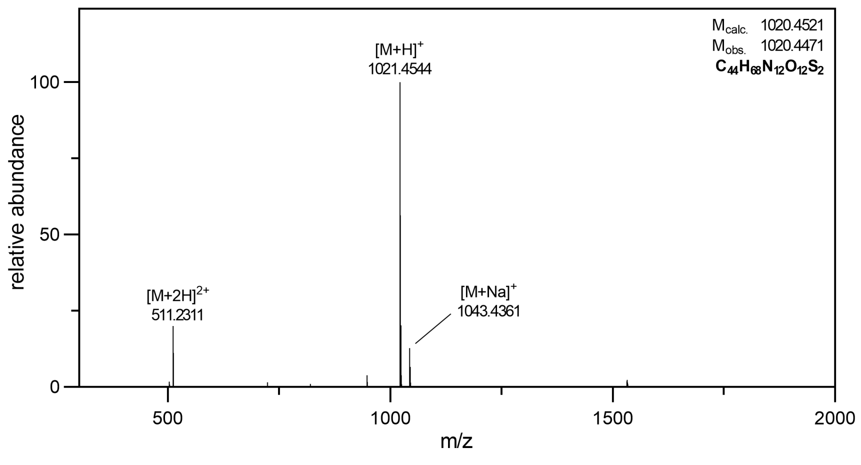
relative abundance
m/z
Mcalc. 1020.4521
Mobs. 1020.4471
C44H68N12O12S2
[M+H]+
1021.4544
[M+2H]2+
511.2311
[M+Na]+
1043.4361
100
50
0
500
1000
1500
2000

(β³-homo)L⁸OT

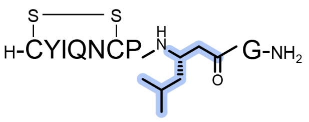

H-CYIQNCP
G-NH2

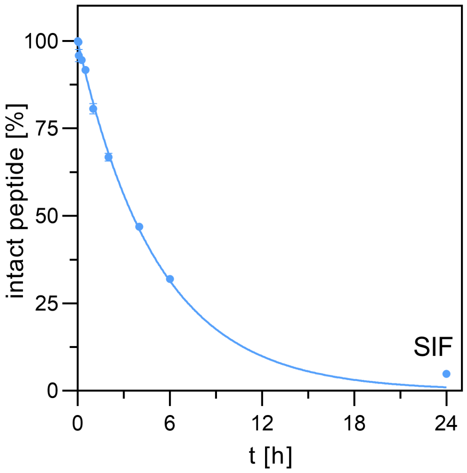

intact peptide [%]
t [h]
SIF

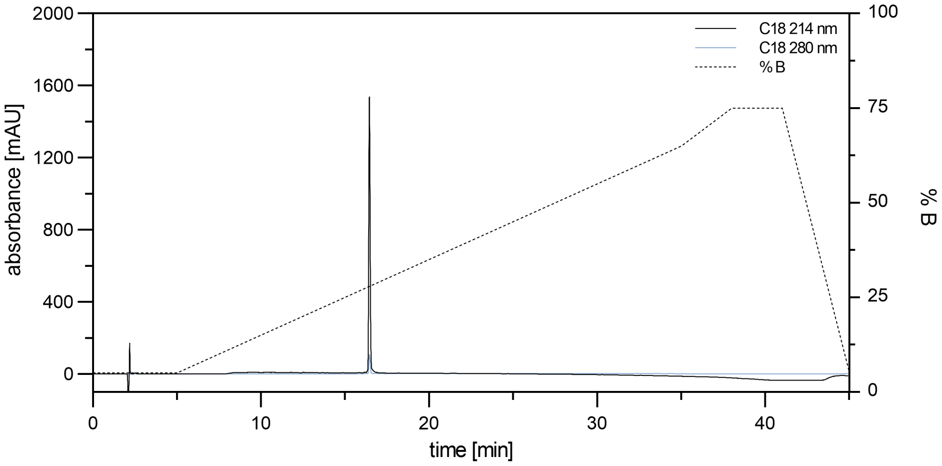

absorbance [mAU]
time [min]
% B
C18 214 nm
C18 280 nm
% B

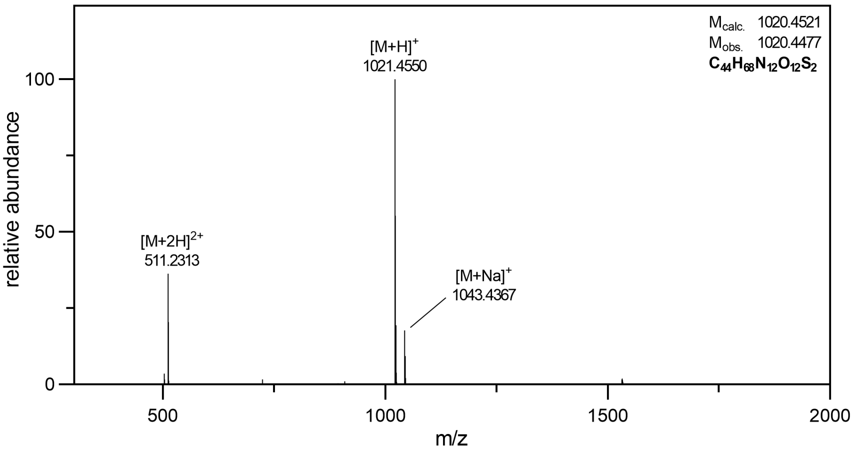

relative abundance
m/z
[M+2H]2+
511.2313
[M+H]+
1021.4550
[M+Na]+
1043.4367
Mcalc. 1020.4521
Mobs. 1020.4477
C44H68N12O12S2

(Nα-Me)G⁹OT

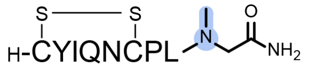

S
S
H-CYIQNCPL
N
O
NH2

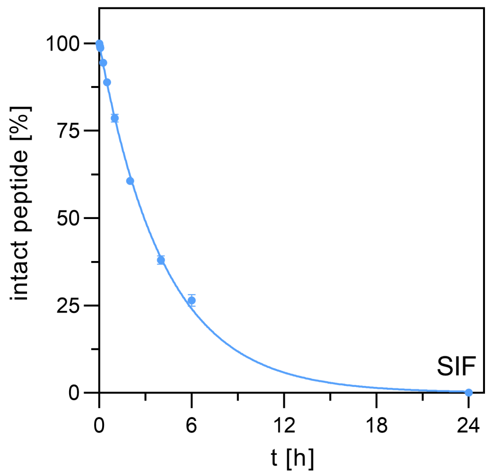

intact peptide [%]
100
75
50
25
0
SIF
0
6
12
18
24
t [h]

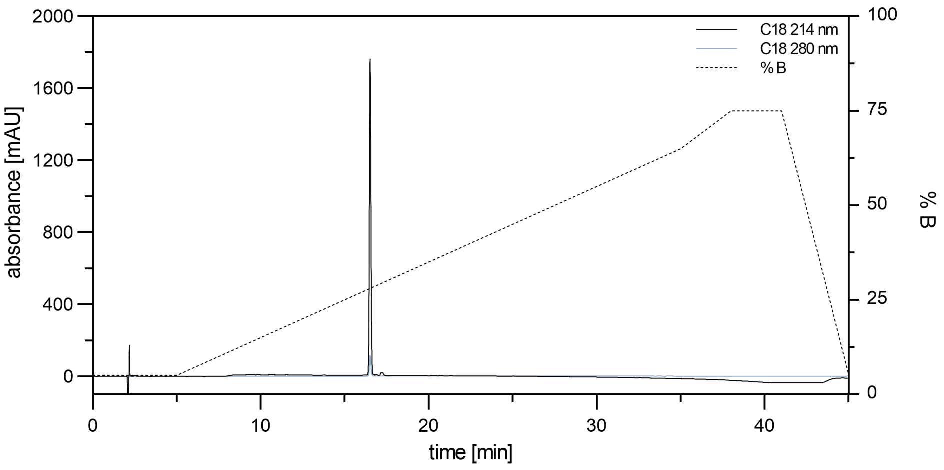

absorbance [mAU]
2000
1600
1200
800
400
0
C18 214 nm
C18 280 nm
% B
100
75
50
25
0
% B
0
10
20
30
40
time [min]

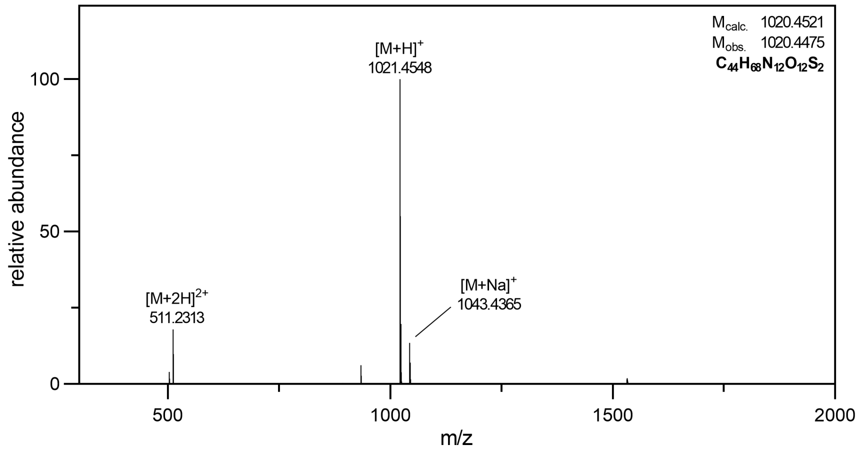

relative abundance
100
50
0
Mcalc. 1020.4521
Mobs. 1020.4475
C44H68N12O12S2
[M+H]+
1021.4548
[M+2H]2+
511.2313
[M+Na]+
1043.4365
500
1000
1500
2000
m/z

G[9](aza)OT

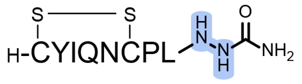
H-CYIQNCPL
S
S
H
N
N
H
O
NH2

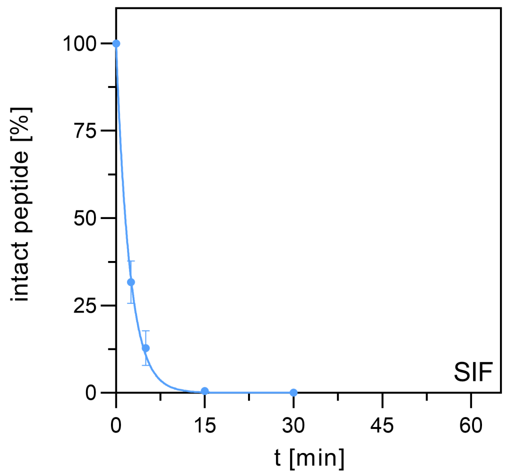
intact peptide [%]
t [min]
SIF
100
75
50
25
0
0
15
30
45
60

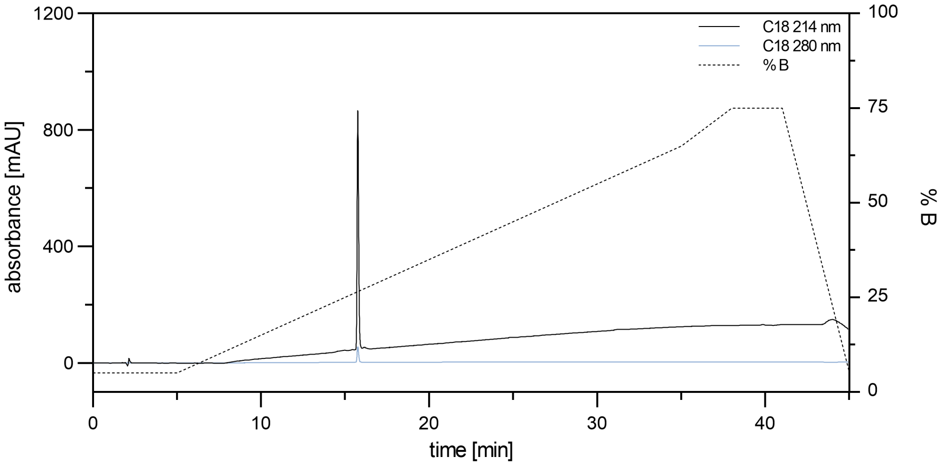
absorbance [mAU]
% B
time [min]
C18 214 nm
C18 280 nm
% B
1200
800
400
0
100
75
50
25
0
0
10
20
30
40

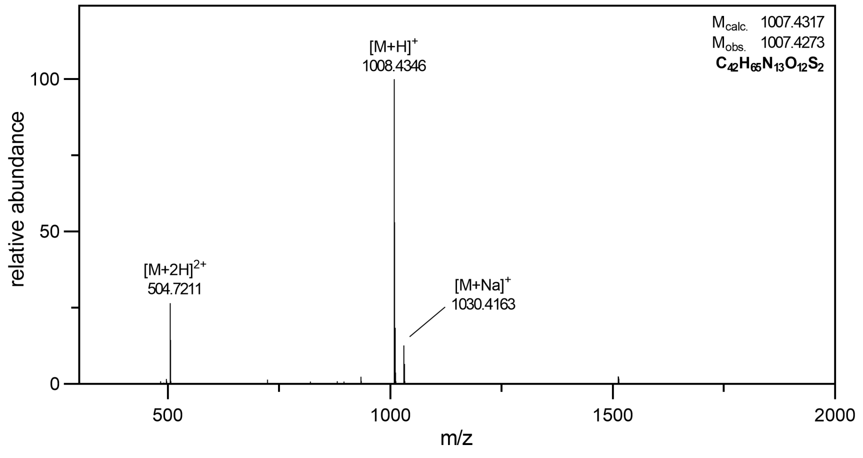
relative abundance
m/z
Mcalc. 1007.4317
Mobs. 1007.4273
C42H65N13O12S2
[M+H]+
1008.4346
[M+2H]2+
504.7211
[M+Na]+
1030.4163
100
50
0
500
1000
1500
2000

c(OT)

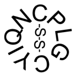

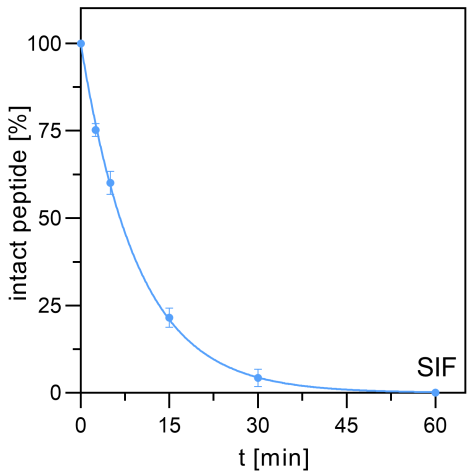

intact peptide [%]
100
75
50
25
0
0
15
30
45
60
t [min]
SIF

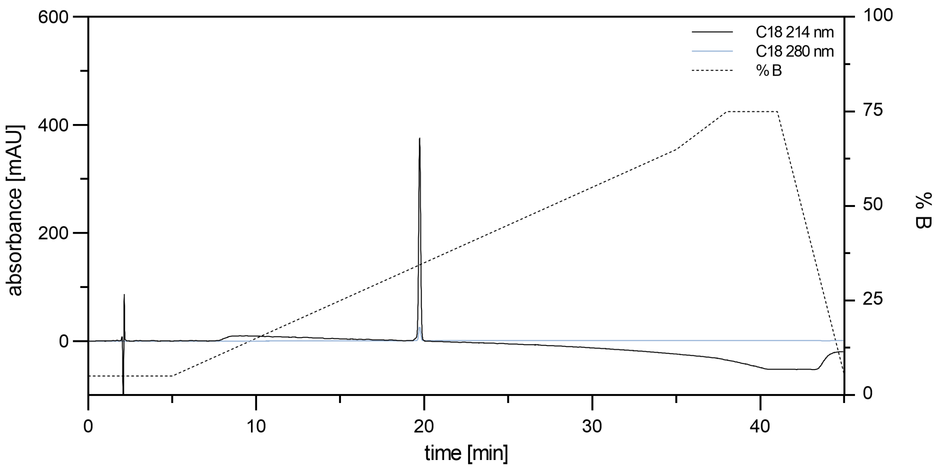

absorbance [mAU]
% B
time [min]
C18 214 nm
C18 280 nm
% B

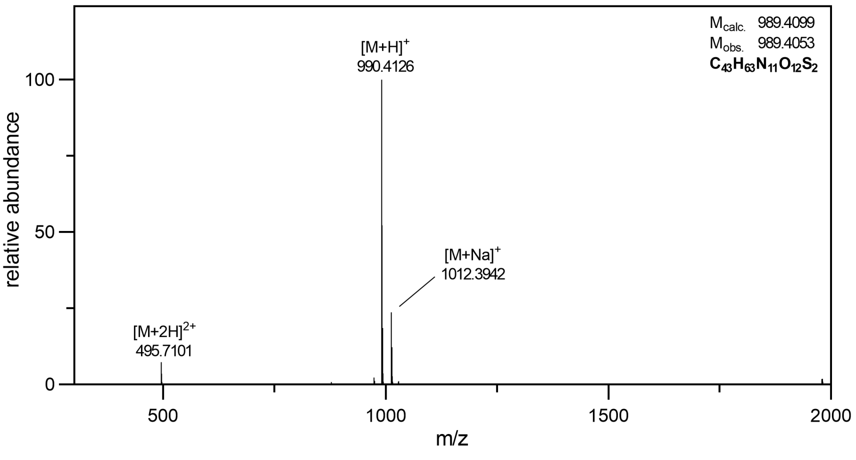

relative abundance
m/z
[M+H]+
990.4126
[M+Na]+
1012.3942
[M+2H]2+
495.7101
Mcalc. 989.4099
Mobs. 989.4053
C43H63N11O12S2

c(linker-OT) 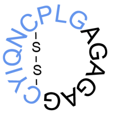
CYIQNCPLGAGAG
S
S

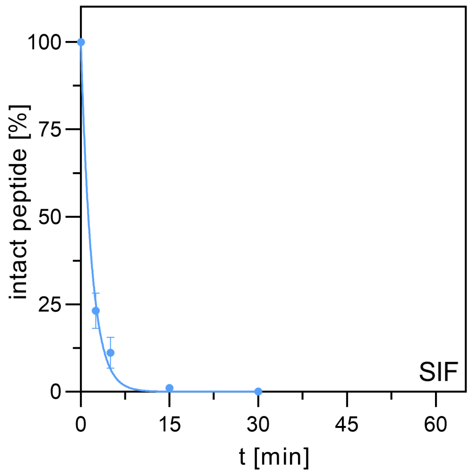
100
75
50
25
0
intact peptide [%]
SIF
0
15
30
45
60
t [min]

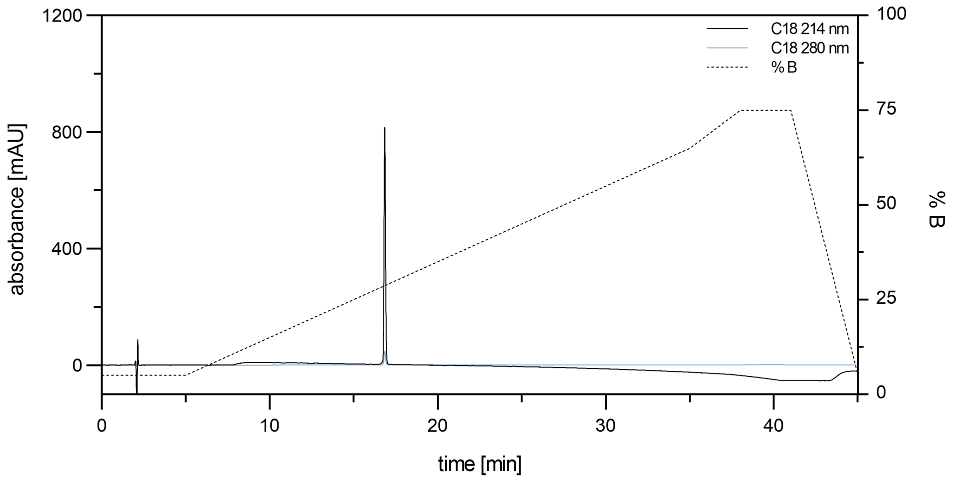
1200
800
400
0
absorbance [mAU]
C18 214 nm
C18 280 nm
% B
100
75
50
25
0
% B
0
10
20
30
40
time [min]

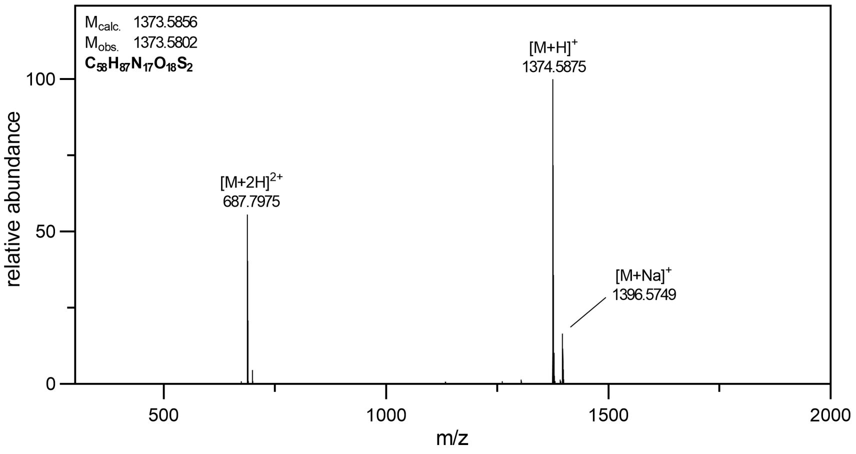
M calc. 1373.5856
M obs. 1373.5802
C58H87N17O18S2
[M+H]+
1374.5875
[M+2H]2+
687.7975
[M+Na]+
1396.5749
100
50
0
relative abundance
500
1000
1500
2000
m/z

c(OT)2

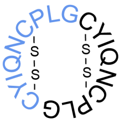

CYIQNCPLG
CYIQNCPLG
S-S
S-S

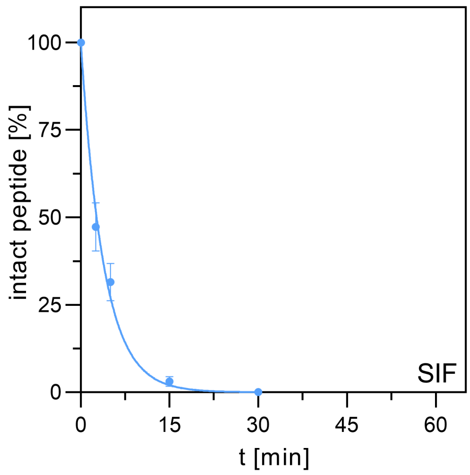

intact peptide [%]
t [min]
SIF
100
75
50
25
0
0
15
30
45
60

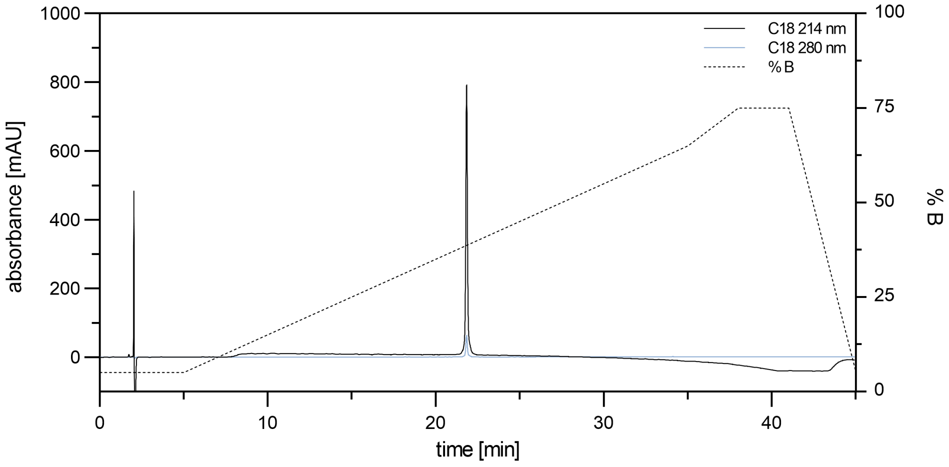

C18 214 nm
C18 280 nm
% B
absorbance [mAU]
time [min]
% B
1000
800
600
400
200
0
0
10
20
30
40
100
75
50
25
0

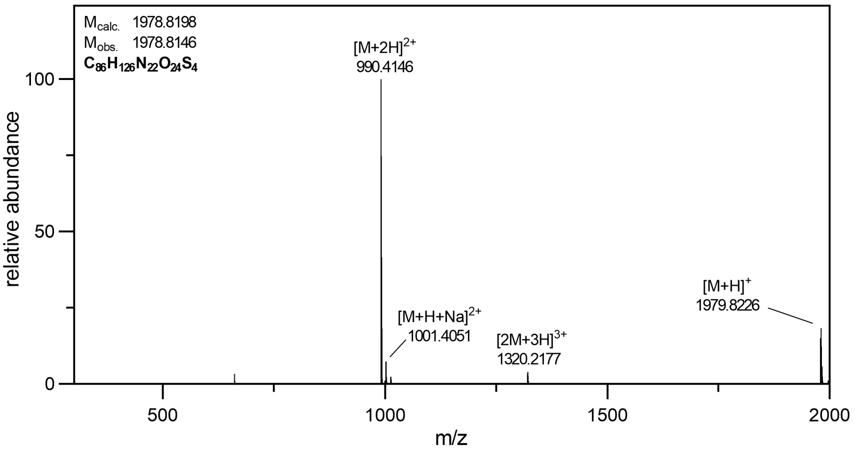

Mcalc. 1978.8198
Mobs. 1978.8146
C86H126N22O24S4
[M+2H]2+
990.4146
[M+H+Na]2+
1001.4051
[2M+3H]3+
1320.2177
[M+H]+
1979.8226
relative abundance
m/z
100
50
0
500
1000
1500
2000

c(OT-VP) 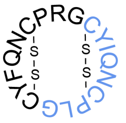

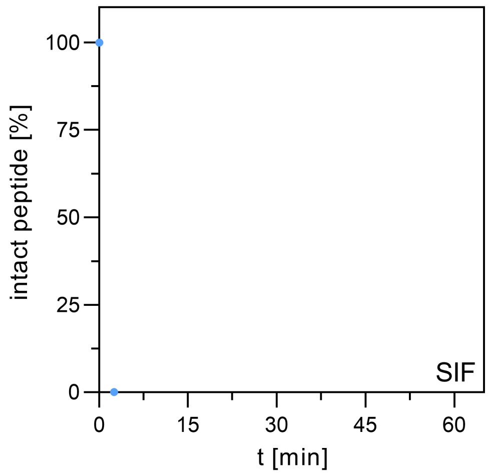

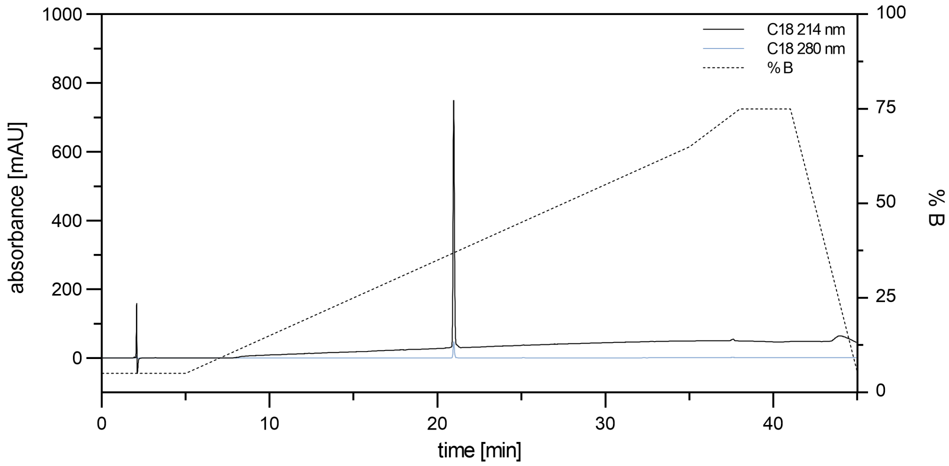

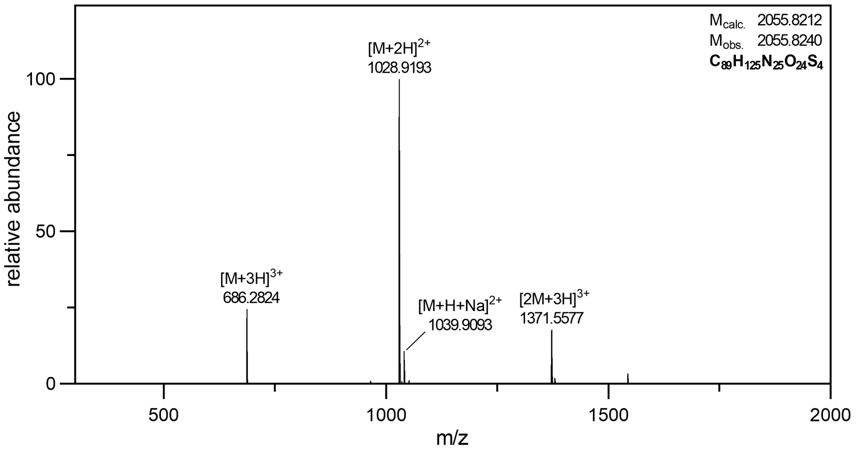

Note: Compound c(OT-VP) was rapidly degraded in SIF with no intact compound detectable at the first sampling time point (2.5 min). The illustrated graph shows t_0 (100%) and $t_{2.5min}$ (0%) without any degradation curve fit.

c(VP)$_2$

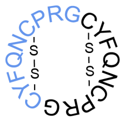

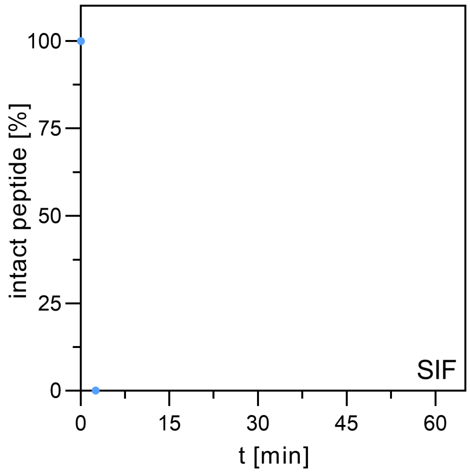

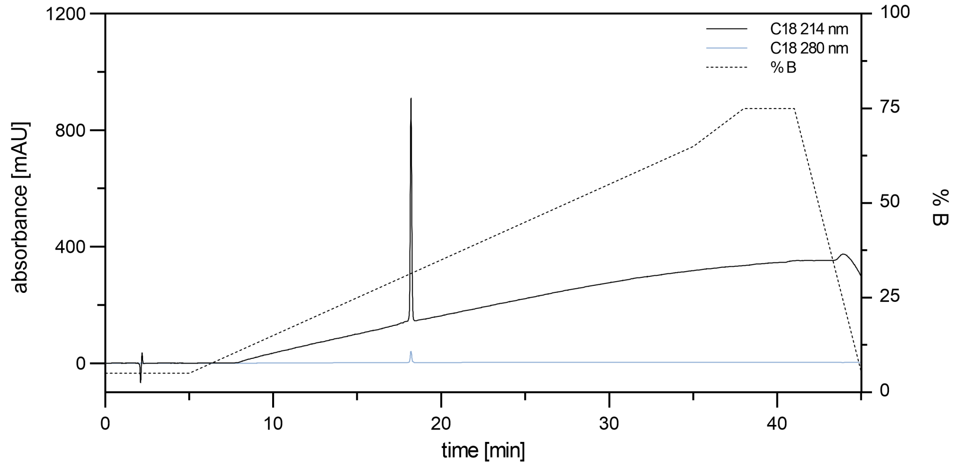

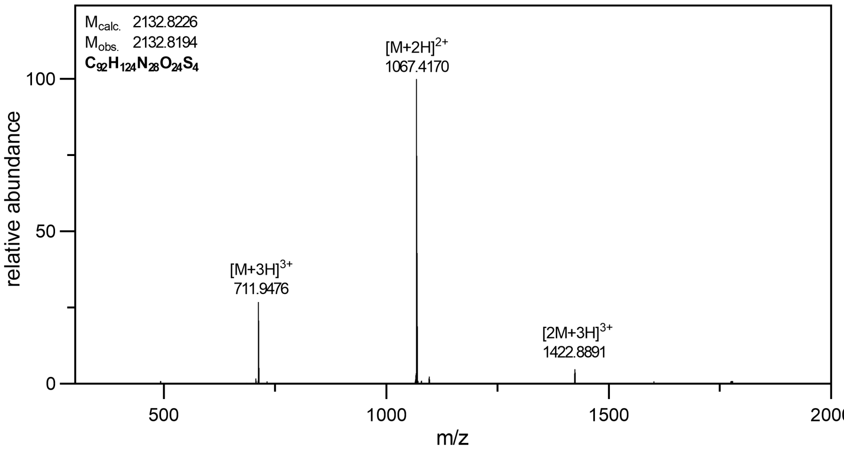

Note: Compound c(VP)$_2$ was rapidly degraded in SIF with no intact compound detectable at the first sampling time point (2.5 min). The illustrated graph shows t_0 (100%) and $t_{2.5min}$ (0%) without any degradation curve fit.

KB1(OT[2-5])

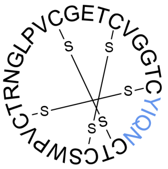

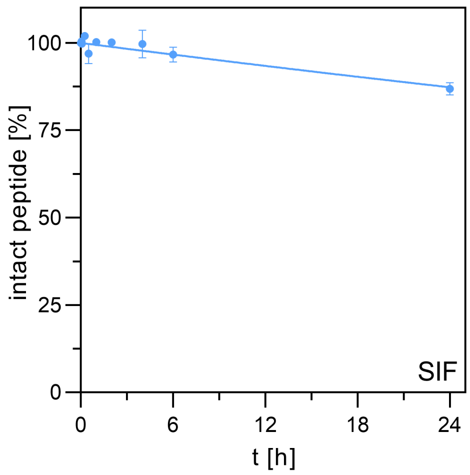

intact peptide [%]
t [h]
SIF

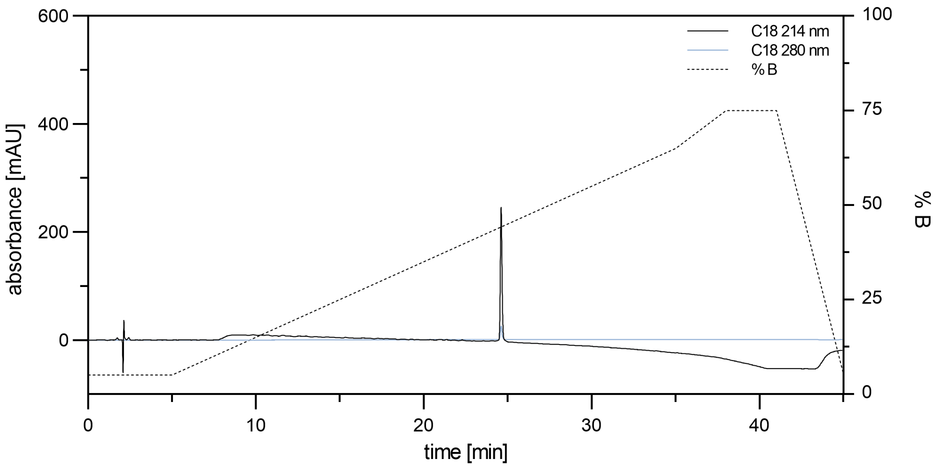

C18 214 nm
C18 280 nm
% B
absorbance [mAU]
time [min]
% B

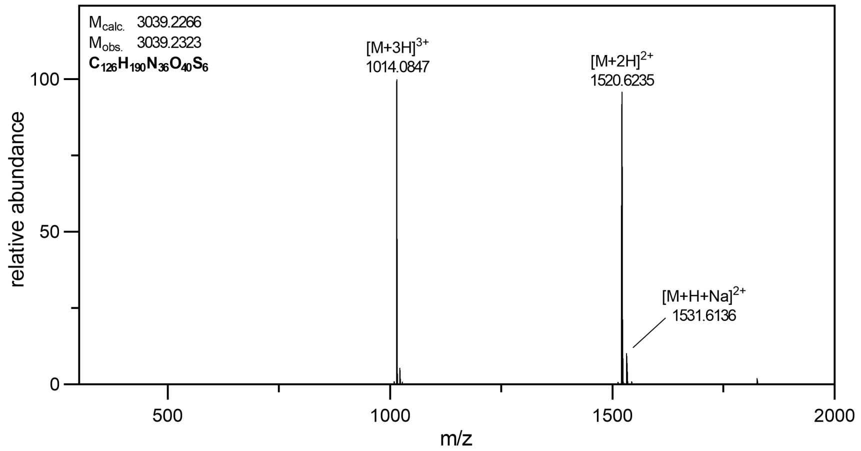

$M_{calc.}$ 3039.2266
$M_{obs.}$ 3039.2323
$C_{126}H_{190}N_{36}O_{40}S_6$
$[M+3H]^{3+}$ 1014.0847
$[M+2H]^{2+}$ 1520.6235
$[M+H+Na]^{2+}$ 1531.6136
relative abundance
m/z

KB1(T[4]G[7]OT[2-5])

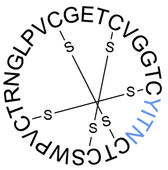

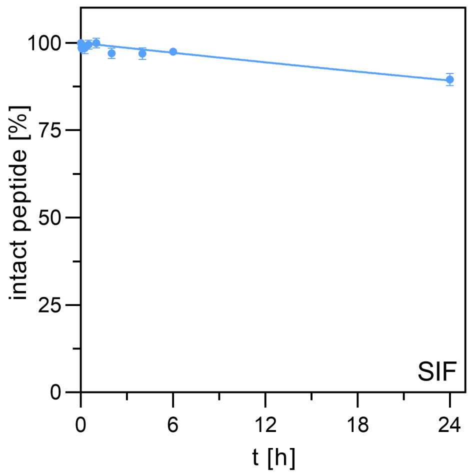

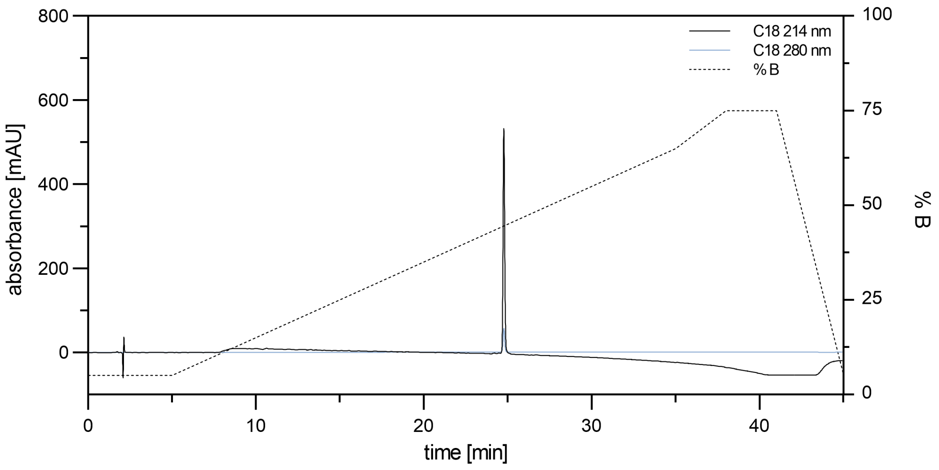

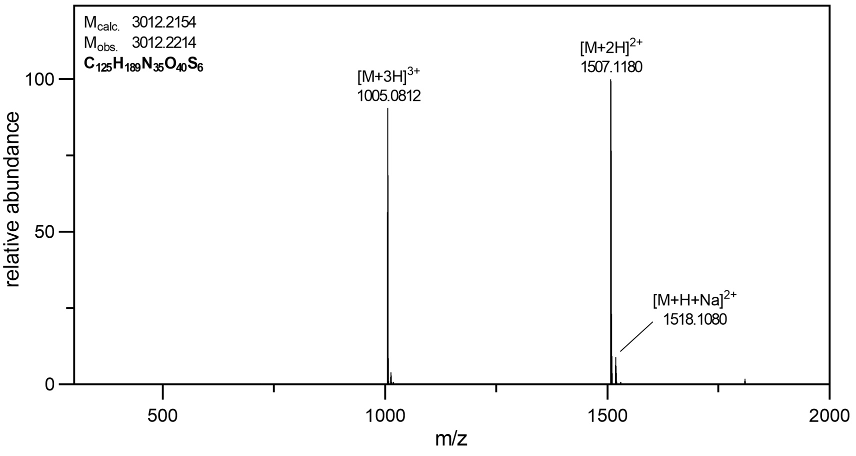

KB7(T^{4}G^{7}OT$^{[2-5]}$)

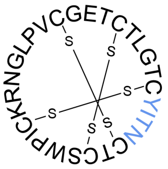

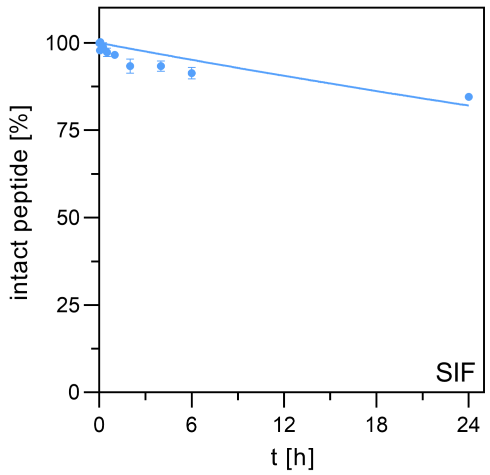

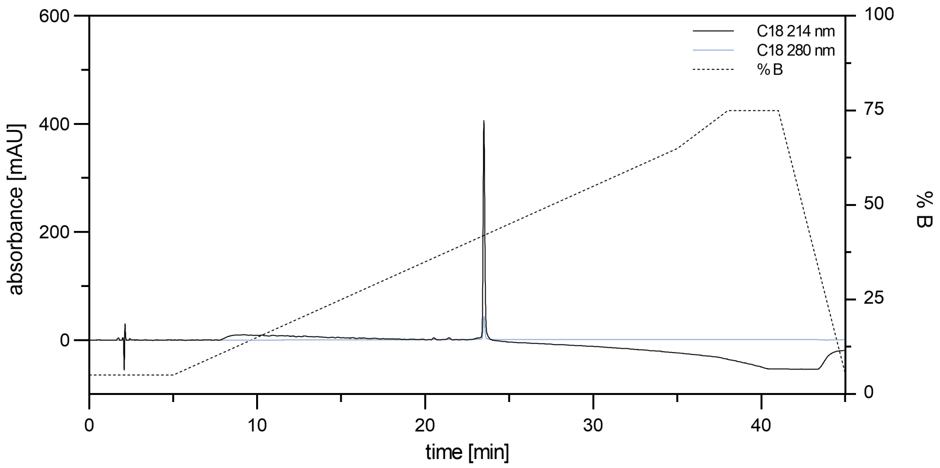

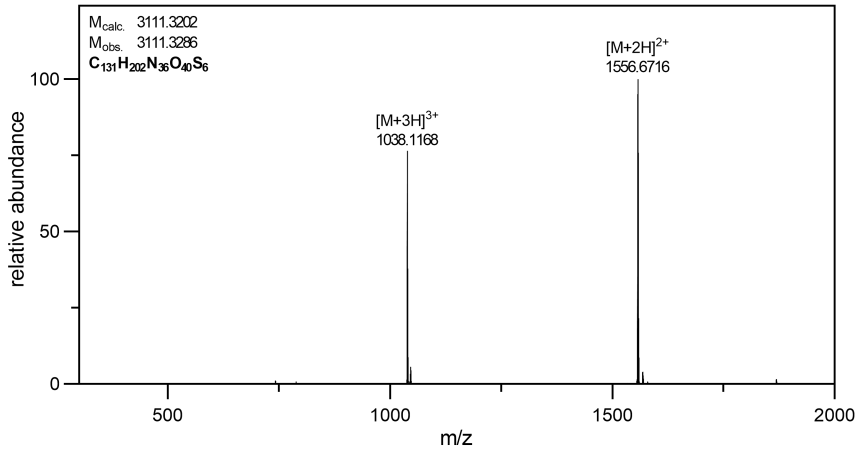

SFTI-1(OT$^{[2-5]}$)

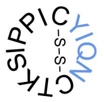

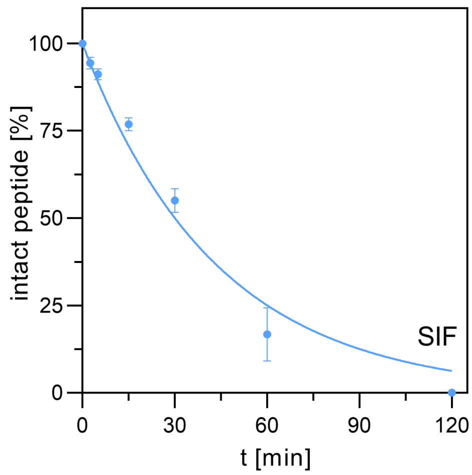
intact peptide [%]
100
75
50
25
0
0
30
60
90
120
t [min]
SIF

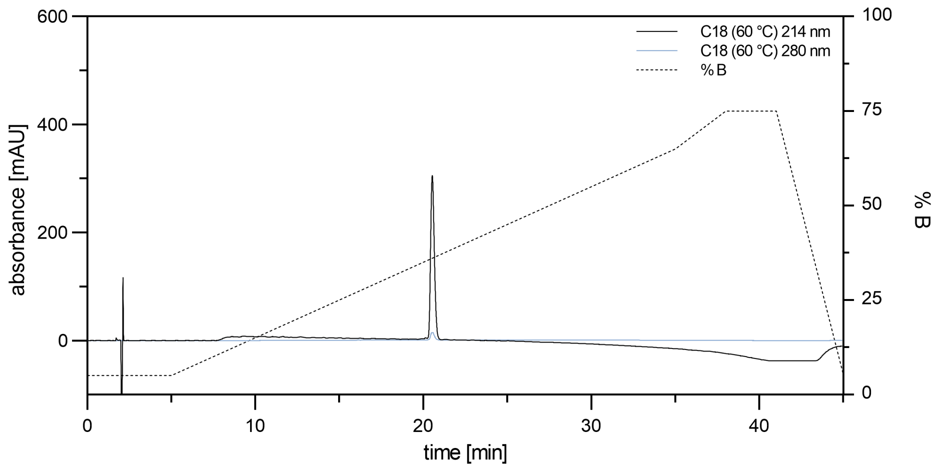
absorbance [mAU]
600
400
200
0
100
75
50
25
0
% B
0
10
20
30
40
time [min]
C18 (60 °C) 214 nm
C18 (60 °C) 280 nm
% B

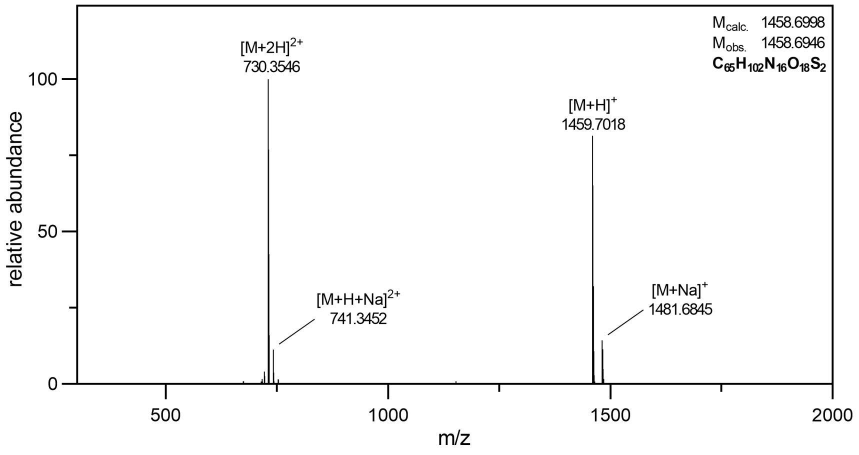
relative abundance
100
50
0
500
1000
1500
2000
m/z
[M+2H]2+
730.3546
[M+H+Na]2+
741.3452
[M+H]+
1459.7018
[M+Na]+
1481.6845
Mcalc. 1458.6998
Mobs. 1458.6946
C65H102N16O18S2

SFTI-1(OT[2-9])

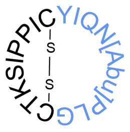

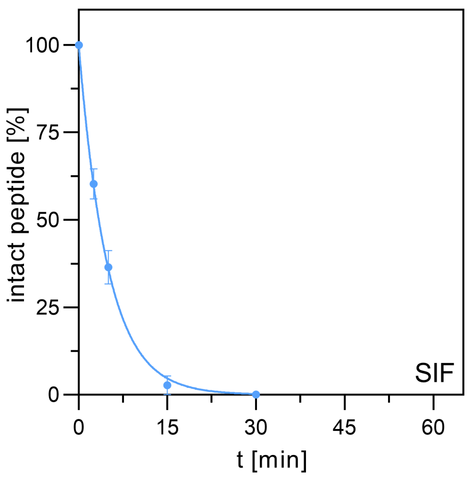

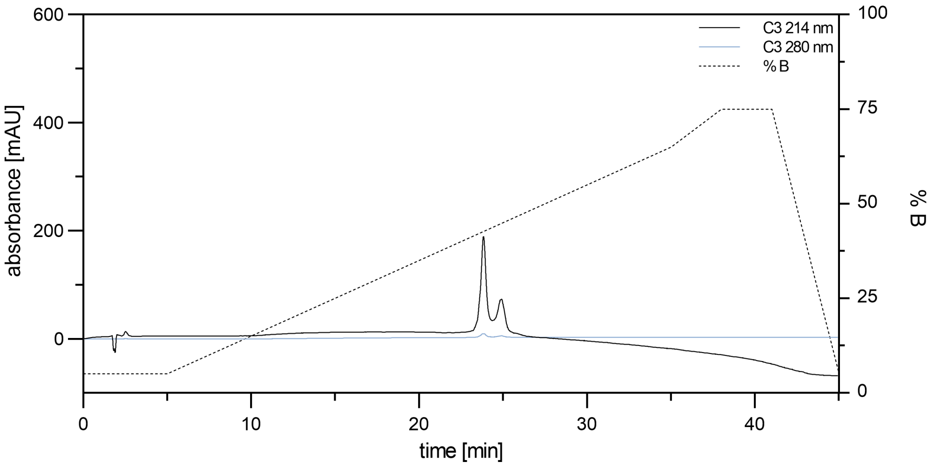

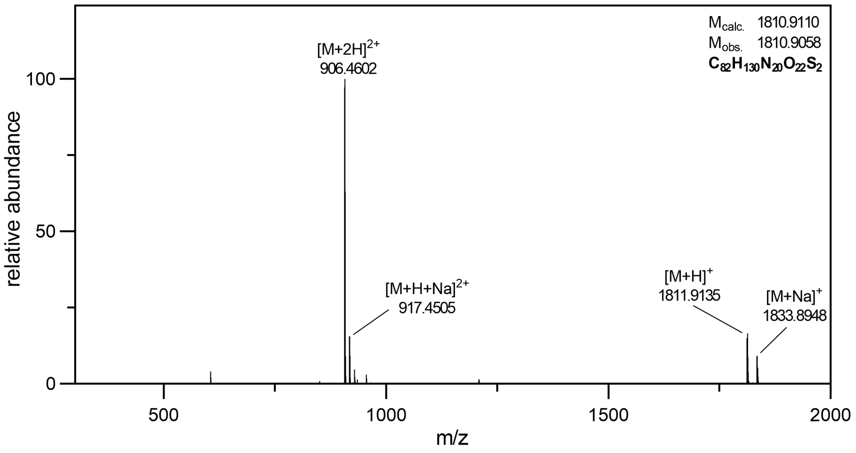

Note: Compound SFTI-1(OT[2-9]) gave a double peak on any tested analytical HPLC column (C_3, C_4 and C_{18}; used here: Agilent Zorbax 300SB-C_3, 2.1x150 mm, 5 µm). Manuel collection of both peaks and re-injection gave the same chromatographic pattern.

SFTI-1(VP[2-5])

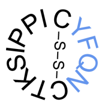

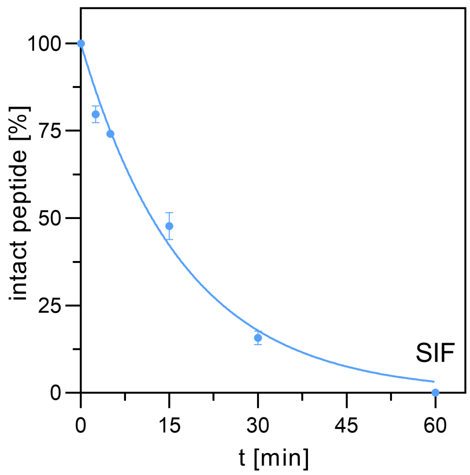
100
75
50
25
0
intact peptide [%]
0
15
30
45
60
t [min]
SIF

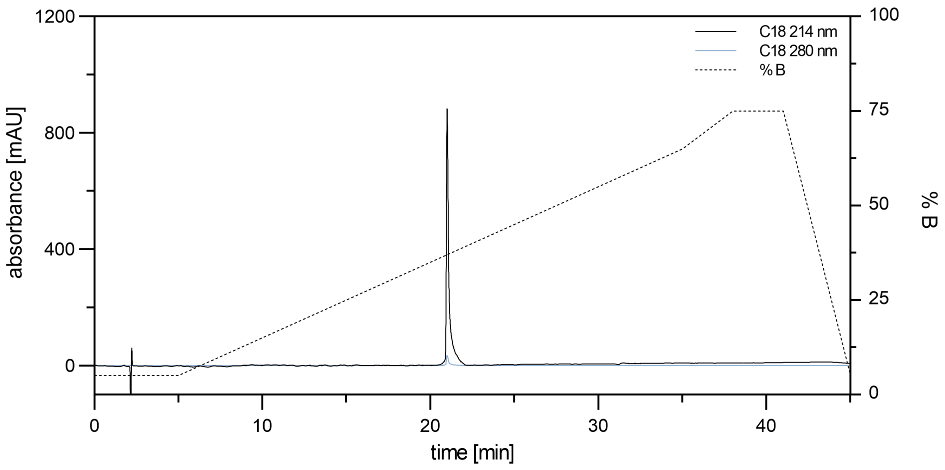
1200
800
400
0
absorbance [mAU]
0
10
20
30
40
time [min]
100
75
50
25
0
% B
C18 214 nm
C18 280 nm
% B

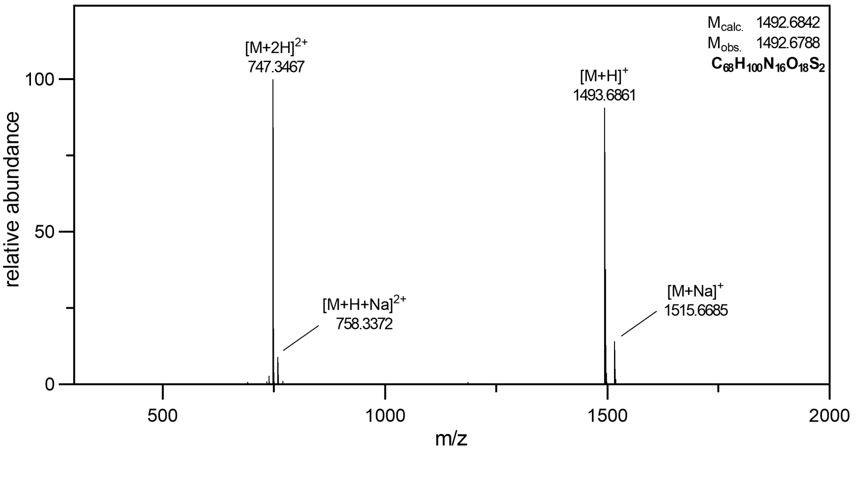
Mcalc. 1492.6842
Mobs. 1492.6788
C68H100N16O18S2
[M+2H]2+
747.3467
[M+H]+
1493.6861
[M+H+Na]2+
758.3372
[M+Na]+
1515.6685
100
50
0
relative abundance
500
1000
1500
2000
m/z

SFTI-1(VP[2-9])

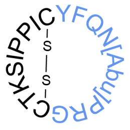

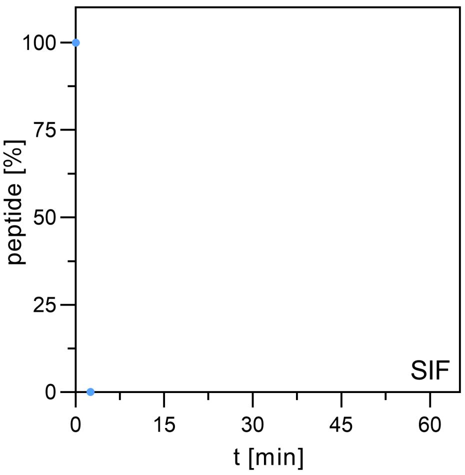

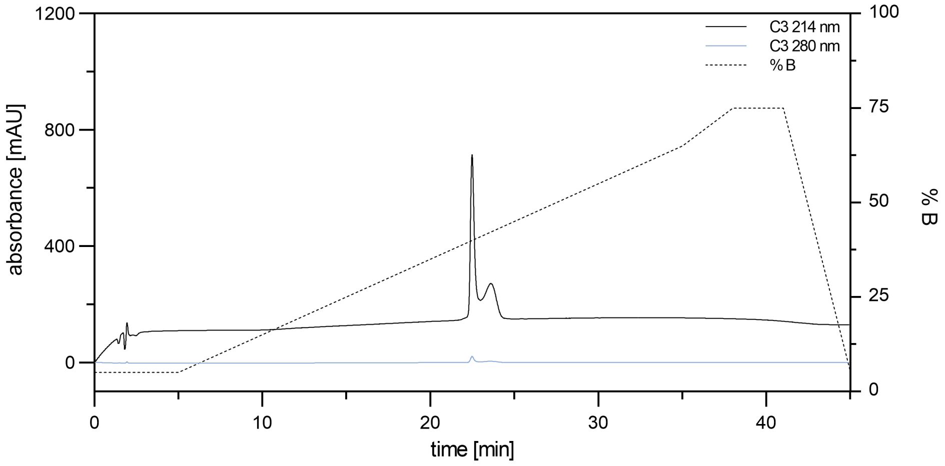

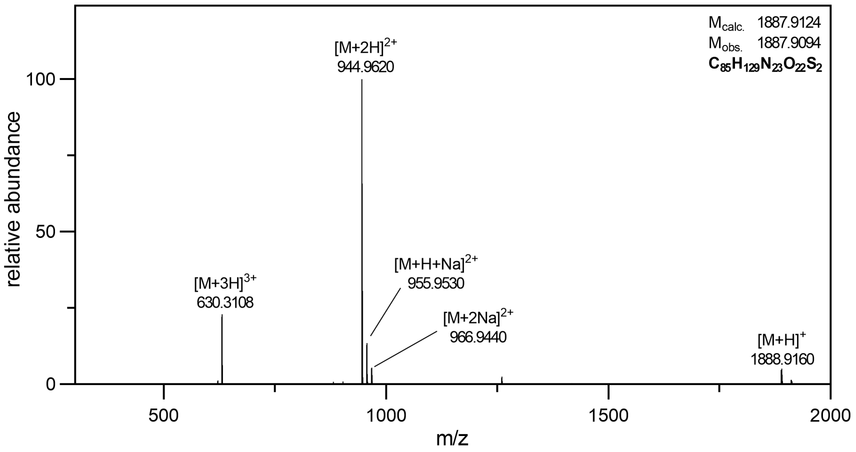

Note: Compound SFTI-1(VP[2-9]) was rapidly degraded in SIF with no intact compound detectable at the first sampling time point (2.5 min). The illustrated graph shows t_0 (100%) and $t_{2.5min}$ (0%) without any degradation curve fit. Compound SFTI-1(VP[2-9]) gave a double peak on any tested analytical HPLC column (C_3, C_4 and C_{18}; used here: Agilent Zorbax 300SB-C_3, 2.1x150 mm, 5 µm). Manuel collection of both peaks and re-injection gave the same chromatographic pattern.

SFTI-1(Anta^{V1aR})

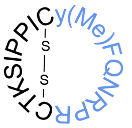

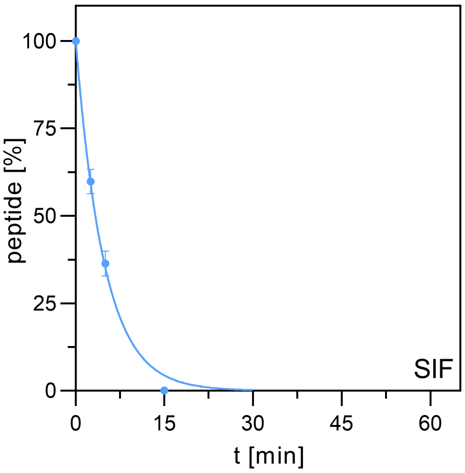

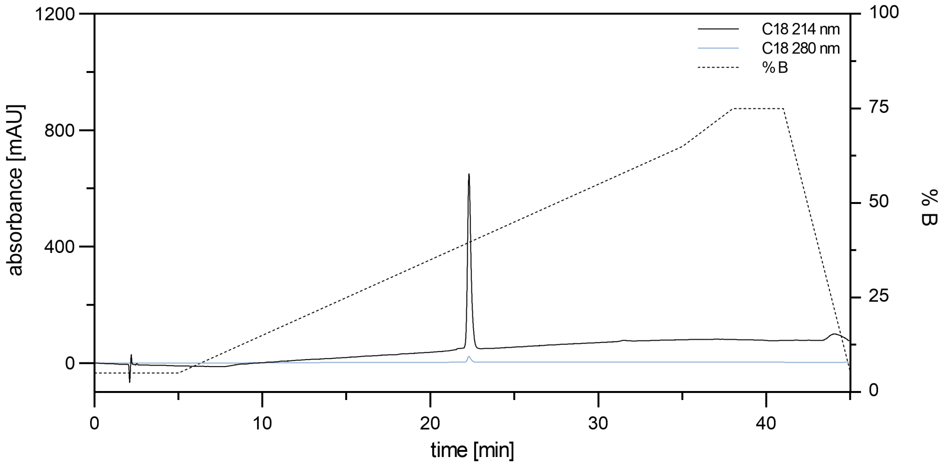

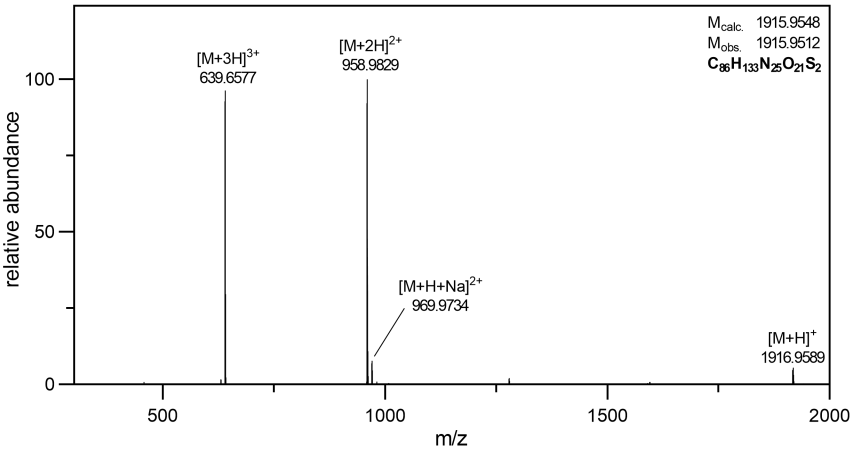

References

1. Kong, X. D.; Moriya, J.; Carle, V.; Pojer, F.; Abriata, L. A.; Deyle, K.; Heinis, C., De novo development of proteolytically resistant therapeutic peptides for oral administration. *Nat. Biomed. Eng.* **2020,** *4* (5), 560-571.

2. United States Pharmacopeia and National Formulary (USP 42 - NF 37); The United States Pharmacopeial Convention: Rockville, Md., 2019

3. Langdon, T. K. In *Pepsin as a case study for method and unit harmonization*, Industry Perspective USP Enzyme Workshop, Rockville, Maryland, Rockville, Maryland, 2009.

4. Gray, V.; Cole, E.; Toma, J.; Ghidorsi, L.; Guo, J.-H.; Han, J.-H.; Han, F.; Hosty, C.; Kochling, J.; Kraemer, J.; Langdon, T.; Leinbach, S.; Martin, G.; Meyerhoffer, S.; Moreton, R.; Raghavan, K.; Shneyvas, E.; Suggett, J.; Tindal, S.; Marques, M., Use of enzymes in the dissolution testing of gelatin capsules and gelatin-coated tablets-revisions to dissolution < 711 > and disintegration and dissolution of dietary supplements < 2040 >. *Dissolut. Technol.* **2014,** *21*, 6-19.

5. Wang, J.; Yadav, V.; Smart, A. L.; Tajiri, S.; Basit, A. W., Toward oral delivery of biopharmaceuticals: An assessment of the gastrointestinal stability of 17 peptide drugs. *Mol. Pharm.* **2015,** *12* (3), 966-973.

6. Piper, D. W.; Fenton, B. H., Ph stability and activity curves of pepsin with special reference to their clinical importance. *Gut* **1965,** *6* (5), 506-508.

7. Fjellestad-Paulsen, A.; Söderberg-Ahlm, C.; Lundin, S., Metabolism of vasopressin, oxytocin, and their analogues in the human gastrointestinal tract. *Peptides* **1995,** *16* (6), 1141-1147.

8. Barth, T.; Pliška, V.; Rychlík, I., Chymotryptic and tryptic cleavage of oxytocin and vasopressin. *Collect. Czech. Chem. Commun.* **1967,** *32* (3), 1058-1063.

9. Tuppy, H., The influence of enzymes on neurohypophysial hormones and similar peptides. In *Neurohypophysial hormones and similar polypeptides*, Berde, B., Ed. Springer Berlin Heidelberg: Berlin, Heidelberg, 1968; pp 67-129.

10. Clark, R. J.; Jensen, J.; Nevin, S. T.; Callaghan, B. P.; Adams, D. J.; Craik, D. J., The engineering of an orally active conotoxin for the treatment of neuropathic pain. *Angew. Chem. Int. Ed.* **2010,** *49* (37), 6545-6548.

11. Clark, R. J.; Craik, D. J., Engineering cyclic peptide toxins. *Meth. Enzymol.* **2012,** *503*, 57-74.

12. Dekan, Z.; Kremsmayr, T.; Keov, P.; Godin, M.; Teakle, N.; Dürrauer, L.; Xiang, H.; Gharib, D.; Bergmayr, C.; Hellinger, R.; Gay, M.; Vilaseca, M.; Kurzbach, D.; Albericio, F.; Alewood, P. F.; Gruber, C. W.; Muttenthaler, M., Nature-inspired dimerization as a strategy to modulate neuropeptide pharmacology exemplified with vasopressin and oxytocin. *Chem. Sci.* **2021,** *12* (11), 4057-4062.

13. Kroeze, W. K.; Sassano, M. F.; Huang, X. P.; Lansu, K.; McCorvy, J. D.; Giguere, P. M.; Sciaky, N.; Roth, B. L., PRESTO-Tango as an open-source resource for interrogation of the druggable human GPCRome. *Nat. Struct. Mol. Biol.* **2015,** *22* (5), 362-369.

14. Muttenthaler, M.; Andersson, A.; de Araujo, A. D.; Dekan, Z.; Lewis, R. J.; Alewood, P. F., Modulating oxytocin activity and plasma stability by disulfide bond engineering. *J. Med. Chem.* **2010,** *53* (24), 8585-8596.

15. Sciabola, S.; Goetz, G. H.; Bai, G.; Rogers, B. N.; Gray, D. L.; Duplantier, A.; Fonseca, K. R.; Vanase-Frawley, M. A.; Kablaoui, N. M., Systematic N-methylation of oxytocin: Impact on pharmacology and intramolecular hydrogen bonding network. *Bioorg. Med. Chem.* **2016,** *24* (16), 3513-3520.
